# Stillbirths at 22- and 28-weeks’ gestation or longer for the states in India: findings from the Global Burden of Disease Study 2023

**DOI:** 10.1016/j.lansea.2026.100819

**Published:** 2026-07-27

**Authors:** India State-Level Disease Burden Initiative Stillbirth Collaborators, Rakhi Dandona, G Anil Kumar, Haley Comfort, Theresa A. McHugh, Bhoomadevi A, Rizwan Suliankatchi Abdulkader, Anirudh Balakrishna Acharya, Gaurav Acharya, Swetha Acharya, Usha Adiga, Mohd Adnan, Dhiraj Motilal Agarwal, Vaishali Aggarwal, Aqeel Ahmad, Danish Ahmad, Naved Ahmad, Saheem Ahmad, Mehrunnisha Sharif Ahmed, Saif Ahmed, Syed Anees Ahmed, Mohd Akbar, Mohammad Jahoor Alam, Irfan Ali, Mohammad Daud Ali, Rafat Ali, Margret Beaula Alocious Sukumar, AH Sruthi Anil Kumar, Prapti Anjana, Kabilan Annadurai, Sadaf Anwar, Razique Anwer, Gururaj Arakeri, Jesu Arockiaraj, Syed Mohammed Basheeruddin Asdaq, Mohammad Saquib Ashraf, Syed Amir Ashraf, Ashish Awasthi, Sheeba B, Prasad Hanmantrao Babar, Giridhara Rathnaiah Babu, Raju Balaji, Senthilkumar Balakrishnan, Altaf Bandy, Bhavik Bansal, Hansi Bansal, Narasimha M. Beeraka, Gulam Saidunnisa Begum, Shahina Begum, Samir Bele, Uzma Iqbal Belgaumi, Om Prakash Prakash Bera, Ajeet Singh Bhadoria, Anuradha Bhardwaj, Nikha Bhardwaj, Pankaj Bhardwaj, Sonu Bhaskar, Shahnawaz Ali Bhat, Arushee Bhatnagar, Priyadarshini Bhattacharjee, Sudip Bhattacharya, Gurjit Kaur Bhatti, Jasvinder Singh Bhatti, Bijit Biswas, Monirujjaman Biswas, Trupti Bodhare, Jyoti Brahmaiah, Sravana Deepthi C, Sanjay C. J, Joshua Chadwick, Rama Mohan Chandika, Baskaran Chandrasekaran, Vijay Kumar Chattu, Anis Ahmad Chaudhary, Sirshendu Chaudhuri, Waseem Chauhan, Akhilanand Chaurasia, Snehal Prakash Chavhan, Saravanan Chinnaiyan, Shivani Chopra, Devasahayam J. Christopher, Mayank Dalakoti, P Vineeth Daniel, Roy Arokiam Daniel, Aso Mohammad Darwesh, Dhruva Dave, Vinoth Gnana Chellaiyan Devanbu, Devananda Devegowda, Sagnik Dey, Arkadeep Dhali, Kuldeep Dhama, Amol S. Dhane, Redolen Rose Dhar, Winniecia Dkhar, S Siva Prasad Dora, Ojas Prakashbhai Doshi, Rajkumar Prakashbhai Doshi, Viola Savy Dsouza, Siddhartha Dutta, Sulagna Dutta, Venkata Ravikanth Eddula, Balisa Mosisa Ejeta, Devaraj Ezhilarasan, Mohammad Fareed, Aisha Farhana, Rohit Ganduboina, Balasankar Ganesan, Balasubramanian Ganesh, Ezhilkugan Ganessane, Shivaprakash Gangachannaiah, Vinod Kumar Garg, Rupesh K. Gautam, Vikram Jeet Singh Gill, Bikash Ranjan Giri, Archit Goel, Rajesh Kumar Goel, Mahaveer Golechha, Sameer Vali Gopalani, Amit Gulati, Sheffali Gulati, Anish Kumar Gupta, Lalit Gupta, Abdul Hafiz, Pritam Halder, Mohd Anul Haq, Shamimul Hasan, Md Saquib Hasnain, Md Imtaiyaz Hassan, Omed Hassan Ahmed, Simon I. Hay, Ramesh Holla, Mehdi Hosseinzadeh, Talib Hussain, Mohammad Tarique Imam, Mohd Imran, Leeberk Raja Inbaraj, Danish Iqbal, C.V. Irshad, Mustafa Alhaji Isa, Jaison Jacob, Vennila Jaganathan, Qazi Mohammad Sajid Jamal, Jerin James, Rajiv Janardhanan, Krishna Mahendrabhai Jasani, Shubha Jayaram, Felix K. Jebasingh, Bijay Mukesh Jeswani, Jobin Jose, Jobinse Jose, Nitin Joseph, Krupal Joshi, Hema Shree K, Vidya Kadashetti, Sanjay Kalra, Mehnaz Kamal, Rajesh Kamath, Suthanthira Kannan S, Subham Kansal, Sunil Kumar Kar, Reddy Praneeth Karnam, Faizan Zaffar Kashoo, Manoj Kumar Kashyap, Ramya Kateel, Avleen Kaur, Harleen Kaur, Sandeep Kaur, Mohd Adnan Kausar, Kanica Kaushal, Abida Khan, Maseer Khan, Mohammad Idreesh Khan, Mohd Faheem Khan, Mohd Wajid Ali Khan, Sumaiya Khan, Wasif Ali Khan, Yusuf Saleem Khan, Parisa Khoshvaght, Samira Khoshvaght, Jagdish Khubchandani, P Ratan Khuman, Sanjay Kini B, Ladli Kishore, Shivakumar KM, Archana Koul, Sindhura Lakshmi Koulmane Laxminarayana, Kewal Krishan, Bindu Krishnan, Nitya Krishnasamy, Ananya Kuanar, Mohammed Kuddus, Anit Kujur, Ashwini Kumar, Chandan Kumar, Dewesh Kumar, Jogender Kumar, Kamal Kumar, Manoj Kumar, Mukesh Kumar, Nithin Kumar, Pawan Kumar, Pradeep Kumar, Radha Kumar, Rakesh Kumar, Sandeep Kumar, Sumit Kumar, Tushar Kumar, Subramanian Kumaran, Senthil Kumaran D, Nisha Kumari, Sajeetha Kumari R, Jibin Kunjavara, Krishna Prasad Kurpad, Pramod Kumar Kushawaha, Pallavi L. C, Chandrakant Lahariya, Anita Lakhani, Dharmesh Kumar Lal, Savita Lasrado, Virendra S. Ligade, Surbala Devi Lourembam, Abhilash Ludhiadch, Abdul Majid, Moutushi Majumder, Bhagabat Malik, Shahid Malik, Tabarak Malik, Lokesh Manjani, Kamaruddeen Mannethodi, Sahana Manoharan, Shaista Manzoor, Vijaya Marakala, J Jenifer Florence Mary, Madhumitha Masilamani, Mansi Mathur, Medha Mathur, Jitendra Meena, Satish Melwani, Godfred Antony Menezes, Ritesh G. Menezes, Giuseppe Minervini, G.K. Mini, Kumar Guru Mishra, Vinaytosh Mishra, Sayan Mitra, Tridip Mitra, Chaitanya Mittal, Dhruti Modi, Taj Mohammad, Omer Mohammed, Syam Mohan, Itismita Mohanty, Siba Sankar Mohanty, Shibani Mohapatra, Afrasim Moin, Ali H. Mokdad, Himel Mondal, Rohith Motappa, Shiv Kumar Mudgal, Reema Mukherjee, Sumoni Mukherjee, B.V. Murlimanju, Satta Muthu Murugan, Sathish Muthu, Raman Muthusamy, Karikalan Nagarajan, Dhayanithi Nagarajan Balachandran, Ganesh R. Naik, Gurudatta Naik, Prasad Nalabothu, Girish Nandakumar, Dhruva Nandi, Vinay Nangia, Sanghamitra Nayak, Mahesh P. A, Jagadish Rao Padubidri, Dimpal Manilal Paija, Debkumar Pal, Vijayakumar Palaniswamy, Sarika Palepu, Sujogya Kumar Panda, Deepshikha Pande Katare, Anuj Kumar Pandey, Romil R. Parikh, Arpit Parmar, Pragnesh Parmar, Bhargav U. Umeshchandra Patel, Kunal Nitinkumar Patel, Mehali Lalitbhai Patel, Mitesh Patel, Neel Navinkumar Patel, Niki Rut Patel, Sangram Kishor Patel, Satyananda Patel, Shivani Patel, Ashlesh Patil, Sandip Shripati Patil, Shankargouda Patil, Sourav S. Patnaik, Mohamed Anas Patni, Apurba Patra, Shrikant Pawar, Arun Mitra Peddireddy, Bala Ganesh Pichamuthu, Ramesh Poluru, Akila Prashant, Pooja Puri, Surabhi Puri, Manas Pustake, Shuby Puthussery, Jagadeesh Puvvula, Shahazad Niwazi Qurashi, Safia Obaidur Rab, Navid Rabiee, Venkatraman Radhakrishnan, Yashpal Singh Raghav, Pracheth Raghuveer, Mohammad Hifz Ur Rahman, Amita Rai, Sunil Kumar Raina, Jeffrey Pradeep Raj, Gunaseelan Rajendran, Judah Rajendran, Pushp Lata Rajpoot, Prashant Rajput, Chitra Ramasamy, Shakthi Kumaran Ramasamy, Balaji Ramraj, Lokesh Rana, Chythra R. Rao, Priyanka Rao, Rajath Rao, Suchetha S. Rao, Shree Rath, Isha Rathi, Lokesh Ravi, Ramya Kundayi Ravi, Renju Ravi, Karthik Rayapureddi, Bhagwan Narayan Rekadwad, Moattar Raza Rizvi, Syed Mohd Danish Rizvi, Hannah Elizabeth Robinson-Oden, Ravi Rohilla, Himanshu Sekhar Rout, Parameswari Royapuram Parthasarathy, Manjula S, Chandan S. N, Mohd Saeed, Rajesh Sagar, Ashok Kumar Sah, Indranil Saha, Amirhossein Sahebkar, Vaibhav Sahni, Ambika Sahoo, Harihar Sahoo, Soumya Swaroop Sahoo, Binaya Krushna Sahu, Kirti Sundar Sahu, Mahesh Chandra Sahu, Sandeep G. Sangle, Rama Krishna Sanjeev, Sathish Sankar, Senthilkumar Sankararaman, Prasanna K. Santhekadur, Sivan Yegnanarayana Iyer Saraswathy, Siddaram Shivaji Sarate, Aswini Saravanan, Sachin C. Sarode, Mukesh Kumar Sathya Narayanan, Maheswar Satpathy, Shakta Mani Satyam, Jennifer Saulam, Ganesh Kumar Saya, Durairaj Sekar, Siddharthan Selvaraj, Vimalraj Selvaraj, Pallav Sengupta, Subramanian Senthilkumaran, Muhammed Shabil, Disha Sanjivkumar Shah, Shipraa Arpan Shah, Sweni Shah, Uzma Shahab, Syed Monowar Alam Shahid, Muhammad Aaqib Shamim, Mohd Shanawaz, Devika Shanmugasundaram, Mohammed Shannawaz, Amit Baran Sharangi, Ankita Sharma, Arkansh Sharma, Avimanu Sharma, Bhoopesh Kumar Sharma, Bunty Sharma, Dolly Sharma, Kamlesh Sharma, Manoj Sharma, Ravi Kumar Sharma, Ujjawal Sharma, Vishal Sharma, Rekha Raghuveer Shenoy, Subuhi Sherwani, Adithi Shetty, B Suresh Kumar Shetty, Mahabalesh Shetty, Premalatha K. Shetty, Shiran Shetty, Suraj S. Shetty, Arun Shirali, Priyanka Arun Shirali, Dharmsheel Shrivastav, Samra Siddiqui, Akanksha Singh, Amit Singh, Baljinder Singh, Brij Pal Singh, Harmanjit Singh, Harpreet Singh, Jasvinder A. Singh, Jawahar Singh, Kalpana Singh, Mitasha Singh, Paramdeep Singh, Poornima Suryanath Singh, Puneetpal Singh, Samer Singh, Satwinder Singh, Shilpy Singh, Siddharth Singh, Surendra Singh, Vanshika Singh, Yadvinder Singh, Abhijeet Prasad Sinha, Mukesh Kumar Sinha, Indra Sivakumar, Farrukh Sobia, Suha Soni, Prashant Sood, Chandrashekhar T. Sreeramareddy, Kannan Sridharan, Vignes Anand Srinivasalu, Shyamkumar Sriram, Vipul Srivastava, Vetriselvan Subramaniyan, Meenakshi Sundari Subramaniyan Natarajan, Alisha Suhag, Thanigaivel Sundaram, Chandan Kumar Swain, Shreya P. Swami, Azharuddin Sajid Syed Khaja, Jabeen Taiba, Jay Tewari, Lakshmi Thangavelu, Kavumpurathu Raman Thankappan, Rekha Thapar, Suresh Thareja, Mahalakshmi Thayumana Sundaram, Jeevarathinam Thirumalai, Muthu Thiruvengadam, Rekha Thiruvengadam, Nihal Thomas, Nikhil Kenny Thomas, Vikas Kumar Tiwari, Marcos Roberto Tovani-Palone, Santhosh Kumar Tumkur Narayanappa, Shadab Uddin, Srikanth Umakanthan, Bhaskaran Unnikrishnan, Era Upadhyay, Dipan Uppal, Rakesh V. R, Asokan Govindaraj Vaithinathan, Sai Mahesh Vajjala, Joe Varghese, Pavani Varma, Sampara Vasishta, Vishnu Priya Veeraraghavan, Anoop Velayudhan, U. Venkatesh, Baskar Venkidasamy, Akshaya Kumar Verma, Poonam Verma, Ramesh Vidavalur, Sharath Chaitanya Vipparthy, Athul Vivek, Hai Nam Vu, Megha Walia, Tanveer A.A. Wani, Dharmveer Yadav, Suresh Yadav, Vikas Yadav, Saba Yahoo, S. Yogeshkumar, Ghazala Yunus, Seema Zargar, Sanjay Zodpey, Christopher J.L. Murray, Lalit Dandona, Austin E. Schumacher, Nicholas J. Kassebaum

**Keywords:** Early gestation, India, Neonatal mortality, State, Stillbirth

## Abstract

**Background:**

The Global Burden of Diseases, Injuries, and Risk Factors Study (GBD), for the first time, estimated stillbirth rate (SBR) by the WHO ICD-11 definition (≥22 weeks' gestation) for 204 countries, highlighting the extent of underestimation in stillbirth burden as compared with using only the late gestation (≥28 weeks’ gestation) SBR estimation to understand the stillbirth burden. Given the size of its population, India accounted for the highest global burden of stillbirths for both gestation period cut-offs. Given the extent of state-level heterogeneity in disease burden in India, state-disaggregated SBR is necessary to enable policymakers and researchers to tailor strategies that are more responsive to local needs and institutional capacities, thereby enhancing the effectiveness of interventions and related resource allocation.

**Methods:**

Data were compiled from 122 sources, including surveys, published studies, and vital and sample registration systems, encompassing 2684 location-year observations. SBRs were estimated for 31 geographic units using spatiotemporal Gaussian process regression to model the ratio of SBR to neonatal mortality rate (NMR), integrating GBD 2023 fertility and all-cause neonatal mortality assessments. Estimates were produced for SBR and number of stillbirths for ≥22 and ≥28 weeks' gestation with 95% uncertainty intervals (UIs) for year 2023. Secondary analyses evaluated the relationship between SBR at the state-level with the Sustainable Development Goal 3 (SDG3; good health and wellbeing) state index score developed by the NITI Aayog and compared GBD results with India's Sample Registration System (SRS) for late gestation SBR and neonatal mortality rate (NMR) for 2023.

**Findings:**

An estimated 565,900 (462,600–702,900) and 347,300 (284,600–430,000) stillbirths occurred in India in 2023, corresponding to SBRs of 25.9 (95% UI 21.3–32.0) and 16.1 (13.2–19.8) at ≥22 weeks' and ≥28 weeks of gestation, respectively. SBR for both definitions varied four-fold between states, from 9.3 (6.8–12.6) in Mizoram to 38.2 (29.0–50.8) in Uttar Pradesh, with the latter and Bihar together accounting for nearly half of India's total stillbirths. A modest inverse correlation was observed between SBR and SDG3 index score (≥22 weeks p = 0.053, r = −0.350 and ≥28 weeks p = 0.034, r = −0.383). Comparison with SRS 2023 revealed substantial under-reporting in SBR, with national late-gestation SBR 2.3 times lower than GBD estimates, though NMR values were consistent across systems.

**Interpretation:**

India's true stillbirth burden is markedly underestimated using the ≥28-week definition. Improved surveillance, tracking stillbirths by the ≥22-week threshold, and integration of stillbirth prevention within broader maternal and newborn health initiatives are essential to address the stillbirth burden in India. Investments are also needed to improve both the availability and quality of data on the magnitude, causes, risk factors, and geographic disparities in stillbirth occurrences for appropriate action to address early gestation stillbirths in India.

**Funding:**

Gates Foundation.

**Research in context**

**Evidence before this study**

Previous analyses of stillbirths in India have relied primarily on national data sources such as the Sample Registration System (SRS), Health Management Information System (HMIS), and National Family Health Surveys (NFHS). These sources capture predominantly late-gestation stillbirths (≥28 weeks' gestation), omitting a substantial proportion of foetal losses that occur earlier in pregnancy. The World Health Organisation's International Classification of Diseases, 11th Revision (ICD-11), defines stillbirth as a foetal death at 22 weeks' gestation or longer; however, few countries, including India, routinely collect or report data below the 28-week threshold due to inconsistencies in surveillance systems and the limited availability of gestational-age–specific data. Consequently, the true magnitude of stillbirths in India remains poorly understood, with previous reports underestimating early foetal deaths and masking regional disparities. To our knowledge, SBR estimates for foetal death at 22 weeks' gestation or longer are not readily available elsewhere for Indian states.

**Added value of this study**

This analysis, conducted as part of the Global Burden of Disease (GBD) 2023 study, provides the first state-level estimates of stillbirths in India at both ≥22 weeks' and ≥28 weeks' gestation using a consistent, model-based framework. Drawing on 122 data sources, including national surveys, published studies, and vital and sample registration systems covering 2684 unique location-year observations, the study employed spatiotemporal Gaussian process regression to model the ratio of stillbirth rate (SBR) to neonatal mortality rate (NMR). Estimates were then harmonised with GBD 2023 fertility and mortality assessments to produce state-disaggregated stillbirth rates and counts for 31 geographic units. Secondary analyses examined associations between SBR and Sustainable Development Goal 3 (SDG3) state index scores, as well as discrepancies between GBD and SRS estimates. By extending analysis to ≥22 weeks’ gestation, this study quantifies the magnitude of underestimation in national stillbirth reporting, highlights substantial state-level heterogeneity, and establishes a comprehensive empirical foundation for policy and programmatic action.

**Implications of all the available evidence**

In 2023, India recorded an estimated 565,900 (95% uncertainty interval [UI] 462,600–702,900) stillbirths at ≥22 weeks' gestation and 347,300 (284,600–430,000) at ≥28 weeks’ gestation, representing a 1.6-fold difference in burden when earlier foetal deaths are included. Stillbirth rates varied four-fold across states, from 9.3 (95% UI 6.8–12.6) per 1000 births in Mizoram to 38.2 (29.0–50.8) in Uttar Pradesh. A modest but significant inverse association between SBR and SDG3 performance scores indicates that stronger maternal and newborn health systems correspond to lower stillbirth rates. These findings demonstrate the urgent need for coordinated multi-sectoral action to strengthen antenatal, intrapartum, and emergency obstetric care, alongside improved data collection and reporting mechanisms. Adopting the ≥22-week reporting threshold, enhancing health information systems, and investing in improving quality and availability of data on causes and risk factors and state-specific interventions will be essential for India to accelerate progress toward stillbirth reduction.

## Introduction

The Every Woman Every Newborn Everywhere (EWENE) partnership calls on countries to achieve a rate of 12 stillbirths or fewer per 1000 total births by 2030.^1^ A stillbirth is a baby born with no signs of life at 22 or more completed weeks' of gestation, as defined by the WHO-ICD 11th Revision.^2^ Stillbirths are further categorised as early gestation stillbirth (at 22 to 27 completed weeks' of gestation) and late gestation stillbirth (at 28 or more completed weeks' of gestation) based on the gestational age. Notably, though the threshold of foetal death at 22 weeks' gestation is recommended for national reporting of stillbirths by the WHO ICD-11, the 28-week gestational threshold (late gestation stillbirths) is used for international comparison due to poor availability and adequacy of data on early gestation stillbirths (between 22 and 28 weeks').^3^ Hence, the true stillbirth burden is underestimated using the 28-weeks’ threshold.

The Global Burden of Diseases, Injuries, and Risk Factors Study (GBD) estimated stillbirth rate (SBR) by the WHO ICD-11 definition for 204 countries, highlighting the extent of underestimation in stillbirth burden as compared with using only the late gestation SBR estimation to understand the stillbirth burden.^4^ India accounted for the highest global burden of the number of stillbirths for both the ≥22 weeks' and ≥28 weeks' gestation period cut-offs.^4^ With India's commitment to a single-digit SBR under the India Newborn Action Plan (INAP),^5^ addressing the burden of stillbirths in India has implications globally for reducing the burden of stillbirths. As the national-level average often obscures critical intra-national disparities, given the extent of state-level heterogeneity in disease burden in India,^6^ state-disaggregated SBR is necessary to enable policymakers and researchers to tailor strategies that are more responsive to local needs and institutional capacities, thereby enhancing the effectiveness of interventions and related resource allocation. The state-level late gestation SBR estimates for India have been reported from the Health Management Information System (HMIS) previously, which are limited both by coverage and quality.^7^, ^8^, ^9^ In this background, we report on the SBR estimates at ≥22 weeks' gestation and ≥28 weeks' gestational threshold for India and its states, modelled for the year 2023 as part of GBD 2023.

## Methods

The analysis of SBR estimation presented in this paper was produced as part of GBD 2023. Comprehensive descriptions of the metrics, data sources, and statistical modelling for SBR and NMR estimates are reported elsewhere.^4^^,^^10^ Below, we provide a broad overview of the methods used to produce SBR estimates, which includes methods to estimate neonatal mortality rate (NMR), deaths in the first 28 days of life. A more detailed description of all methods used is provided in Appendix 1 p3–5. Input data sources and results are available for download from the Global Health Data Exchange at https://ghdx.healthdata.org/record/ihme-data/gbd-2023-demographics-1950-2023. This study complies with the Guidelines for Accurate and Transparent Health Estimates Reporting (GATHER) recommendations.^11^

Data seeking for stillbirths was built on the approach used throughout GBD: namely, to identify, review, and extract all available data sources globally. For stillbirths, this included household surveys, national reports, vital registration, sample registration, and any additional sources listed on the Global Health Data Exchange, supplemented with published studies that were representative of the general population, identified through a systematic literature review through PubMed.^4^ Stillbirth data for India were evaluated from 122 sources, including 6 surveys from the National Family Health Surveys, 29 published studies, 2 vital statistics reports (VR), and 2684 unique location-year combinations from vital and sample registration systems (SRS) for 11 different definitions of stillbirth based on gestational age and/or birthweight. The input sources for the estimation of SBR for India and its states are shown in Appendix 1, pp6–9. Scientific literature input was identified for thirteen states, with the states of Bihar, Maharashtra, Karnataka, and Uttar Pradesh contributing the most (Appendix 1, p10). Adjustments were made for completeness and for survey data reporting period incidence. Data were reviewed and systematically outliered if set criteria were not met. Then, a flexible network meta-regression tool was utilised to standardise all data points to each reference definition (22 and 28 weeks’ gestation).^12^ Lastly, a three-stage model comprised of an ensemble linear mixed-effects model, spatiotemporal smoothing, and Gaussian process regression generated estimates of SBR and stillbirth counts for each reference definition by location and year. NMR was modelled using VR and SRS data as well as complete birth history, summary birth history, and recent household events data from surveys and censuses. Sex and age-specific all-cause mortality rates were estimated for each location using a single statistical model called OneMod that includes parametric and non-parametric components.^10^

The SBR estimates are reported for ≥22 weeks' and for ≥28 weeks' (also referred to as late gestation SBR) for the year 2023 for India and the 31 geographical units of India. To improve the policy uptake of the findings, we assessed the relationship between state-level SBR at ≥22 weeks' of gestation and late gestation SBR with the Sustainable Development Goal 3 (SDG3; good health and wellbeing) state index score developed by the National Institution for Transforming India (NITI Aayog), the apex public policy thinktank of the Government of India, for the year 2023–24.^13^ This score ranges from 0 to 100, indicating the overall progress of the state in achieving the SDG target, with higher scores indicating higher achievement.^13^ Furthermore, we estimated the number of stillbirths for both definitions for India and its states in 2023 based on the respective SBR estimates using GBD 2023's estimated 21.6 million births as the population denominator.^10^ Comparisons of the state-level late gestation SBR estimates in GBD 2023 with the SBR estimates from SRS 2023 are reported to assess the gap between these estimates.^14^ Lastly, because higher under-reporting of stillbirths compared to neonatal deaths has previously been documented in SRS compared to GBD and National Family Health Surveys (NFHS),^15^ we report on the ratio of late gestation SBR to NMR at the state-level in GBD 2023, and compare the SRS 2023 and GBD 2023 NMR estimates with the aim to highlight the continued invisibility of stillbirths as compared to neonatal deaths.

The data from CSV files were copied into MS–Excel to generate the analysis presented, and QGIS was used for generating the India maps.^16^ The 31 geographical units of India included the 28 states; the union territory of Delhi, the two union territories of Jammu & Kashmir and Ladakh combined, and the other small union territories combined (Andaman and Nicobar Islands, Chandigarh, Dadra and Nagar Haveli, Daman and Diu, Lakshadweep, and Puducherry). We also categorised the states as the Empowered Action Group (EAG: Bihar, Chhattisgarh, Jharkhand, Madhya Pradesh, Odisha, Rajasthan, Uttar Pradesh and Uttarakhand), north-east (NE) states, and other states for the analysis to be policy relevant.^17^^,^^18^ All estimates are reported with 95% uncertainty intervals (UIs) where relevant, which were based on 1000 draws for each estimate, with the mean taken as the final estimate and the 2·5th and 97·5th percentiles comprising the 95% UIs.

### Role of funding source

The funder had no role in study design, data collection, data analysis, interpretation, writing of the report.

## Results

**Fig. 1:**
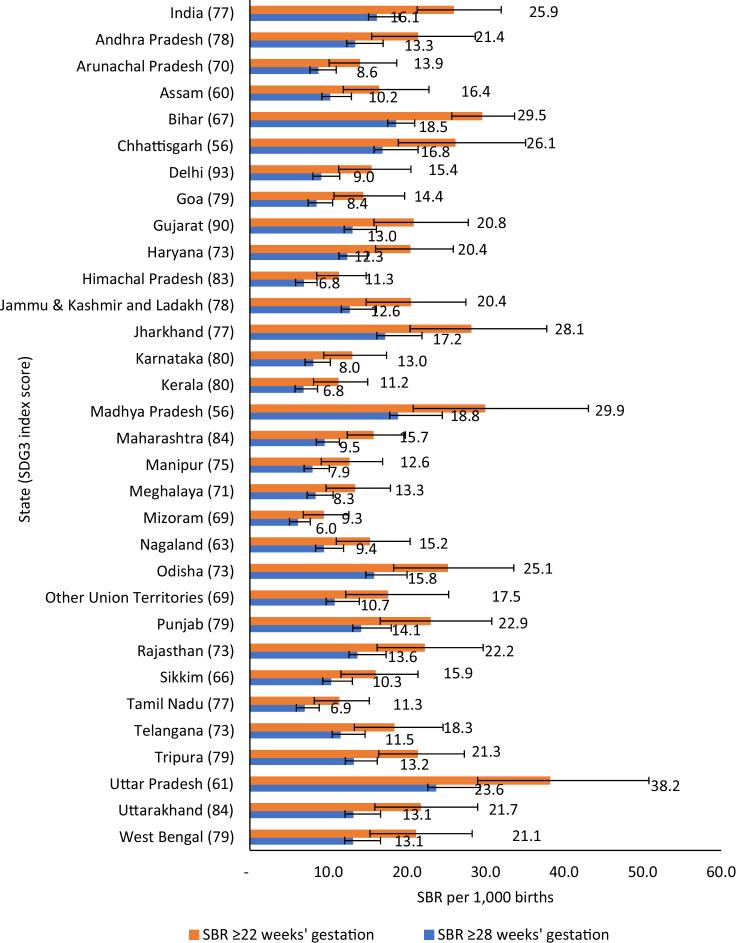
State-level stillbirth rate (SBR) at ≥22 weeks’ and ≥28 weeks’ gestation in 2023. SDG index score refers to the state Sustainable Development Goal 3 index score as computed by NITI Aayog.

**Fig. 2:**
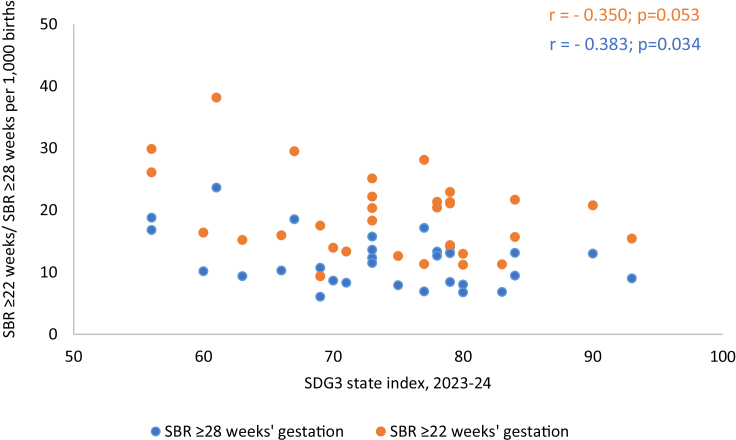
Relationship between state stillbirth rate (SBR) at ≥22 weeks’ and ≥28 weeks’ of gestation with the state Sustainable Development Goal 3 (SDG3) index score in 2023–24. r is Pearson correlation coefficient.

The estimated SBR at ≥22 weeks' for India in 2023 was 25.9 (95% UI 21.3–32.0) and late gestation SBR was estimated at 16.1 (95% UI 13.2–19.8) per 1000 births (Appendix 1, p11). The SBR at ≥22 weeks' varied 4.1 times between the states, ranging from 9.3 (95% UI 6.8–12.6) in Mizoram to 38.2 (95% UI 29.0–50.8) in Uttar Pradesh (Fig. 1, Appendix 1, p11). The late gestation SBR also varied 3.9 times at the state-level, ranging from 6.0 (95% UI 4.4–8.1) in Mizoram to 23.6 (95% UI 18.0–31.5) in Uttar Pradesh (Fig. 1, Appendix 1, p11). Five states, Uttar Pradesh, Bihar, Madhya Pradesh, Jharkhand and Chhattisgarh had SBR for both gestation cut-offs higher than the national average. The state SDG3 index score ranged from 56 in Chhattisgarh to 93 in Delhi (Appendix 1, p11). A modest correlation was seen between the SDG3 state index score and both the SBR estimates at ≥22 weeks' (p = 0.053; r = −0.350) and ≥28 weeks’ gestation (p = 0.034; r = −0.383), with decreasing SBR seen with increasing SDG3 state index score (Fig. 2).

**Fig. 3:**
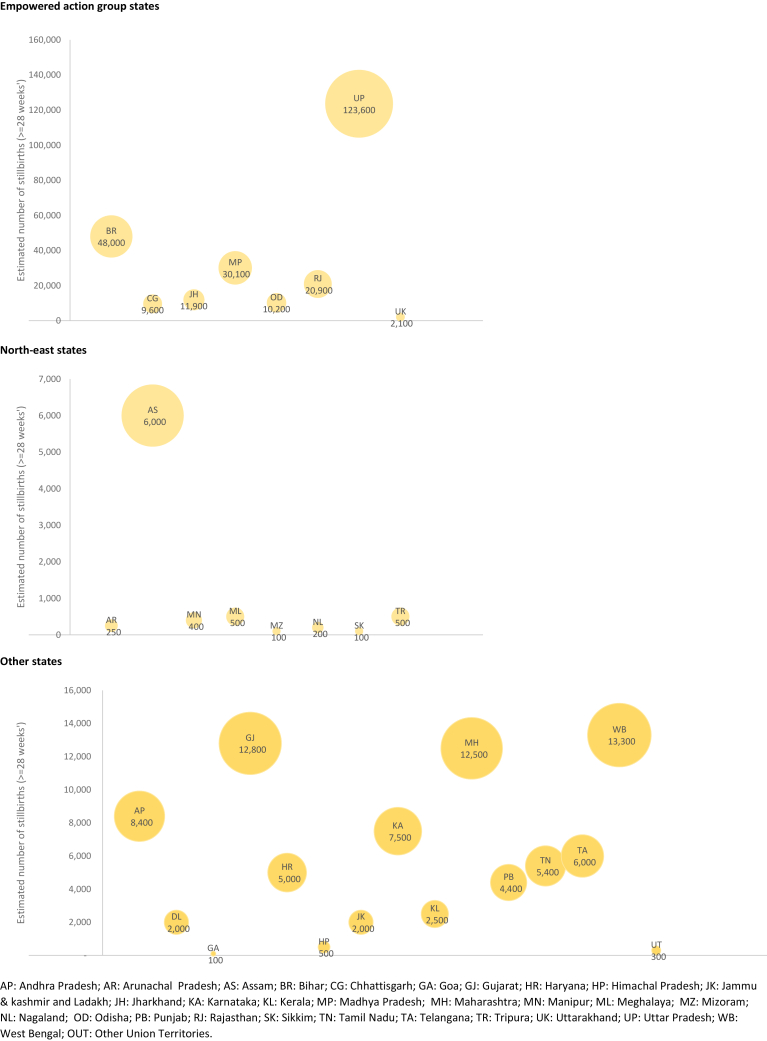
Estimated number of late gestation stillbirths (≥28 weeks’) in Indian states in 2023. The area of the circles is proportional to the estimated number of late gestation stillbirths.

**Fig. 4:**
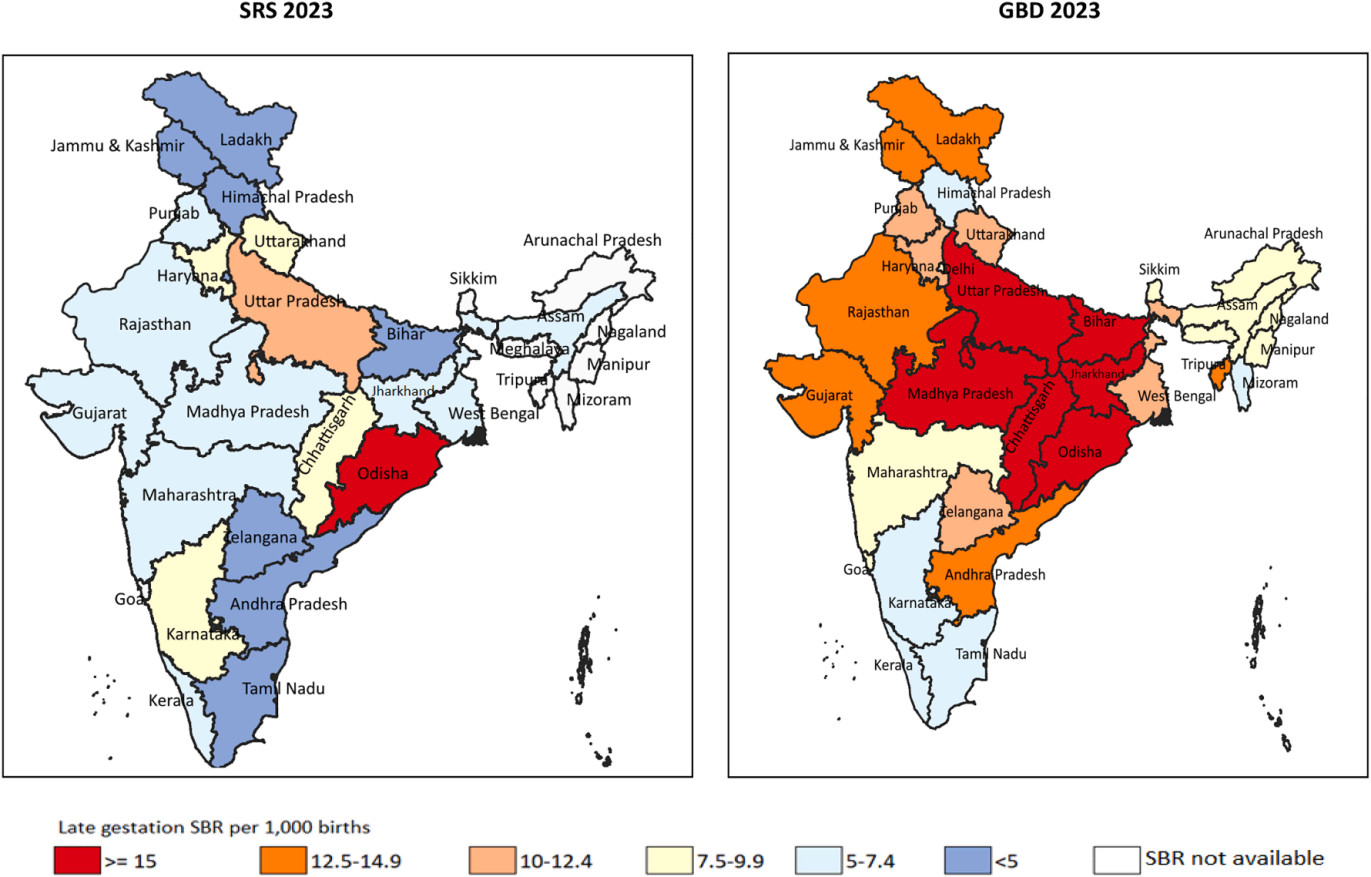
Comparison of state-level late gestation stillbirth rate (SBR) from the Sample Registration System (SRS) and from the Global Burden of Disease (GBD) study for the year 2023.

Using the SBR at ≥22 weeks' and late gestation SBR estimates, India was estimated to have 565,900 (95% UI 462,600–702,900) stillbirths at ≥22 weeks' gestation and 347,300 (95% UI 284,600–430,000) late gestation stillbirths in 2023 (Appendix 1, p12). The largest numbers of estimated stillbirths were in Uttar Pradesh at 202,500 (95% UI 152,300–272,700) and 123,600 (95% UI 93,400–165,700) stillbirths at ≥22 weeks' and ≥28 weeks' gestation, respectively, accounting for 35.6% of the national burden of stillbirths (Fig. 3; Appendix 1, p12). In addition to Uttar Pradesh, Bihar also had a significant number of stillbirths (13.8% of the national burden), followed by Madhya Pradesh (8.7% of the national burden) among the EAG states. Assam accounted for most of the estimated number of late gestation stillbirths across the north-eastern states (Fig. 4). Among the other major states, West Bengal (13,300; 95% UI 9600–18,000), Gujarat (12,800, 95% UI 9700–17,300) and Maharashtra (12,500; 95% UI 9900–15,900) together contributed to nearly 11.2% of India's total late gestation stillbirths count (Fig. 3; Appendix 1, p12).

The SBR to NMR estimates ratio for India and the states as estimated by GBD 2023 are shown in Appendix 1, p13. The ratio of late gestation SBR to NMR in GBD 2023 varied widely across the states, with 13 states having a late gestation SBR to NMR ratio above the national average of 0.84. This indicates similar estimates for NMR and late gestation SBR, except for Kerala, which had a ratio of 1.55. In all, 9 states had a late gestation SBR to NMR ratio of less than 0.75, indicating lower estimates for late gestation SBR than NMR.

The state-level comparison of late gestation SBR between SRS 2023 and GBD 2023 shows under-reporting in the former (Fig. 4 and Appendix 1, p14). The India late gestation SBR in SRS was 2.3 times lower, and under-reporting was particularly high in Andhra Pradesh (6.7), Bihar (4.6), Telangana (3.8), and Jammu & Kashmir and Ladakh (3.2). Only Karnataka and Odisha states had similar SBR estimates between SRS 2023 and GBD 2023. On the other hand, the comparison of NMR between GBD 2023 and SRS 2023 indicated a relatively better alignment than SBR, with a national GBD to SRS ratio of 1 (Appendix 1, p14). This ratio for most states was also close to 1.0, with some discrepancies seen in Assam (ratio: 0.8), Haryana (0.8) and Madhya Pradesh (0.8) with lower GBD estimates than the SRS, and with higher GBD than SRS estimates in Punjab (ratio: 1.2), Delhi (1.2), Bihar (1.2) and Jammu & Kashmir and Ladakh (1.5).

## Discussion

The burden of stillbirths is unevenly distributed across India. At the threshold recommended for national reporting of stillbirths, almost 566,000 babies were stillborn in India at or beyond 22 weeks' of gestation in 2023, corresponding to an estimated one stillbirth every minute. The SBR estimate for India >22 weeks' of gestation was 1.6 times higher than at >28 weeks’ of gestation, the definition most used for international comparison. The difference in the state-disaggregated SBR estimates with both thresholds was four times, highlighting the need to respond to the local context, identify specific challenges, and design targeted interventions to reduce stillbirths locally.

Notably, the threshold recommended for national reporting of stillbirths is ≥22 weeks' gestation.^2^ However, the estimation of early gestation stillbirths from national data remains challenging due to variability in the capture of stillbirth data around the thresholds of viability and differences in the recording of late termination of pregnancies across countries, even in high-income settings.^19^^,^^20^ This is also an important consideration for interpretation of SBR at >22 weeks' gestation for India due to very limited data sources capturing >22 weeks' gestation as compared with many sources available for ≥28 weeks' gestation. As a result, the estimates for ≥22 weeks' stillbirths have relied heavily on statistical modelling and extrapolation from later gestational ages and covariates, leading to wider uncertainty intervals. These estimates should therefore be interpreted cautiously, recognising that the true burden may differ from the modelled values. While the inclusion of ≥22 weeks’ stillbirths in the GBD has allowed gauging the magnitude of the problem comprehensively, it has also highlighted that very limited data availability and quality continue to be constraints to make precise estimates for many locations, including for India, as one in three countries globally do not produce quality data on stillbirths.^19^ The major data sources for these modelled estimates in GBD for India were the various rounds of the NFHS across all Indian states, which were supplemented with scientific literature from select states based on availability and quality. HMIS was also not utilised for modelling in the reported estimates due to the known issues of coverage and quality^21^, ^22^, ^23^, ^24^, ^25^; in particular with the two states with the highest burden of stillbirths in India, Uttar Pradesh and Bihar, reported to have abnormally low prevalence of stillbirths in HMIS.^9^^,^^26^^,^^27^ The SRS data were considered but not used in the modelling as these were outliered by the model; methodology issues that result in under-reporting of stillbirths in the SRS have been identified previously.^28^ The finding of nearly similar NMR estimates between SRS and GBD and the 2 to 16 times lower reporting of late gestation SBR in SRS than GBD across the states has also been reported previously between NFHS and SRS.^15^ This is in line with a review of policies from 155 countries that highlighted that the current policy environment in many countries is not supportive for identifying stillbirths and recording causes of death, as compared with that for neonatal and under-5 deaths, thereby, contributing to continued slow progress in stillbirth reduction.^29^ Furthermore, a recent survey capturing stillbirths from nine Indian states reported only 1% of stillbirths to be registered despite it being mandatory under the Birth and Death Registration Act of India to register stillbirths.^30^^,^^31^ The undercount documented in SRS, HMIS, and stillbirth registration underscores the continued invisibility of stillbirths in the system and also signifies the urgency in improving primary data availability and quality on stillbirths.^32^ Reporting gaps for stillbirths in India could be addressed through a combination of measurement, system, and digital reforms. First, routine audit of gestational age—using standardised clinical criteria and improved documentation at the point of care—would reduce misclassification between miscarriage and stillbirth, thereby, improving reporting of early gestation stillbirths. Second, greater harmonisation of stillbirth definitions and gestational age thresholds across national data systems, including the HMIS, SRS, and NFHS, would improve internal consistency and comparability of estimates. Third, stillbirth registration can be improved by ensuring that stillbirth notification is provided by the facility to the vital registration system, as is the process with neonatal death.^31^ Finally, integration of facility- and survey-based reporting frameworks with the digital architecture of the Ayushman Bharat Digital Mission offers an opportunity to strengthen real-time capture, linkage, and validation of pregnancy outcomes, particularly for losses occurring at earlier gestational ages.^33^

Our analysis demonstrated a modest inverse relationship between the SDG3 index score and SBR across India's states, suggesting that better performance on health-related SDG indicators may correlate with improved perinatal outcomes. While higher SDG3 state index scores appears to be associated with lower stillbirth rates, most states have a relatively high SDG3 state index score, limiting the discriminatory power of this measure; therefore, caution is warranted in overinterpreting this inverse relationship as the index may not fully capture nuanced differences in maternal and reproductive health performance across the states. The nearly similar ratio of late-gestation SBR to NMR across some states suggests that the relative contribution of stillbirths and neonatal deaths to perinatal mortality is comparable, even if absolute rates differ. States with higher absolute rates but similar ratios may require strengthened health system interventions across the continuum of care to reduce both stillbirths and neonatal deaths. On the other hand, ratio <0.8 suggest neonatal deaths exceed stillbirths, indicating gaps in newborn care aimed at improving neonatal survival. Another perspective to be considered is that comparing state performance with the national average can help in understanding where the care is falling short. For instance, Uttar Pradesh has both NMR and SBR higher than the national average, pointing to challenges across the entire care pathway from antenatal and intra-natal to neonatal services. In contrast, Jharkhand exhibits a higher SBR but a lower NMR, indicating that problems are more prevalent during pregnancy rather than after birth. These patterns can help the policymakers evaluate where care needs strengthening and where resources can have the greatest impact.

One-third of the stillbirths in India are estimated to be in the state of Uttar Pradesh, and half of all stillbirths are accounted for by Uttar Pradesh and Bihar. A variety of risk factors for stillbirths in these two and other Indian states have been previously documented, including deferred and referred deliveries, preeclampsia or eclampsia, severe postpartum and antepartum haemorrhage, multiple births, previous history of stillbirth and preterm birth, and mother's age at birth.^28^^,^^30^^,^^34^, ^35^, ^36^, ^37^, ^38^, ^39^, ^40^, ^41^, ^42^ These identified risk factors, which are across the antenatal, labour, and delivery period, indicate that further meaningful reduction in stillbirths can be achieved by focussing on the risk factors across the continuum of maternal care services. Though the essential preventive and maternal health services are largely the same for both early and late gestation stillbirths, post-delivery clinical management and viability-related interventions differ. Considerable investment and significant advances have been made in India to improve labour and delivery care services,^43^^,^^44^ and similar attention is urgently needed to improve the quality of antenatal care services to contribute to addressing all types of pregnancy losses, including early gestation stillbirths.^45^, ^46^, ^47^, ^48^ Of note is also action needed on the varied gendered determinants across the states—including limited female education, restricted decision-making autonomy, and low health literacy—which delay recognition of pregnancy complications and timely utilisation of antenatal and intrapartum care, thereby, increasing the likelihood of stillbirths.^9^ Notably, much of the understanding on the causes and risk factors for stillbirths in India comes from the mother's self-report in surveys and little from clinical data across the continuum of care for pregnant women.^15^^,^^34^^,^^49^ A recent effort has been undertaken by the Indian Council of Medical Research (ICMR) stillbirth pooled India cohort to identify and quantify risk factors and develop a predictive risk stratification model for evidence-based clinical decision-making in high-risk pregnancies.^42^ Policymakers need these data to treat and reduce the causes and risk factors linked to stillbirth and to ensure survival of all babies. India has established a sentinel and facility-based stillbirth surveillance system to systematically collect data on stillbirth rates, associated maternal and foetal risk factors, and causes of stillbirth.^50^ However, perinatal death audits are not always carried out following a stillbirth in many settings, including in India, and the cause of stillbirth is commonly reported as ‘unknown’ or ‘unspecified’.^19^^,^^51^, ^52^, ^53^ Specific focus to improve clinical documentation for every pregnant woman under the Ayushman Bharat Digital Mission, which aims to improve health outcomes through a robust digital health infrastructure for India, can be considered to address the gaps in data on causes and risk factors for both early and late gestation stillbirths.^33^

Each of the 566,000 babies stillborn in India at or beyond 22 weeks' of gestation in 2023 reflect the magnitude of parents and families who are devasted by this loss. The far-reaching impact of these deaths on parents’ physical and emotional well-being is not fully considered in many settings despite the significant economic and psychosocial consequences of stillbirths.^54^ Developing structured bereavement support services for parents and families experiencing stillbirth is essential to address the profound psychological, emotional, and social impact of such loss. ICMR is investing in developing such services as relevant for India to promote respectful bereavement support services for those impacted.^55^

Several limitations to this analysis should be considered. First, GBD framework, on which this analysis depends on, assumes that SBR completeness tracks with NMR, which might not always be the case. Second, the precision of our modelled estimates in particular for ≥22 weeks gestation stillbirths is hindered by a sparsity of primary data on early gestation stillbirths. Third, extensive efforts were undertaken to correct for biases and standardise data, which are limited because not all data sources provide documentation on the definitions used, and our statistical approaches do not account for potential measurement error in gestational ages or weights used to inform adjustments. Household surveys remain an important source of stillbirth data in India, and classifying an adverse pregnancy outcome like stillbirth requires accurate reporting of vital status at birth, gestational age, or birthweight for every pregnancy by participating women in these surveys. Addressing the issues identified in misclassification and misreporting of these parameters is a limitation, which is beyond the scope of the present analysis.^15^^,^^34^^,^^56^ Fourth, we did not attempt to add estimates for underlying cause, timing (ie, intrapartum *vs* antepartum), or preventable versus non-preventable stillbirths, which are needed in informing local policy, research, education, and clinical practice.

In conclusion, stillbirths impose a significant burden across Indian states, reflecting deep inequities in maternal and newborn care. While inclusion of stillbirths from 22 weeks and beyond in this analysis allowed us for a more complete estimation of the magnitude of stillbirths in India, the findings remain constrained due to sparse data availability and poor data quality to make precise estimates. In addition to state-specific actions to strengthen timely and high quality antenatal and intrapartum services to prevent foetal loss across the full continuum of gestation, improved surveillance is also needed for gestational threshold monitoring to improve counting and addressing of overall stillbirth burden in India and globally. Such actions would enable comprehensive understanding of the burden of perinatal mortality for health system and families to allow assessment of variations needed in clinical practice, address risk factors, and improve care to end preventable stillbirths in India as part of its maternal and child health agenda.

## Contributors

Please see Appendix 2 pp14–19 for more detailed information about individual author contributions to the research, divided into the following categories: writing the first draft of the manuscript; providing data or critical feedback on data sources; developing methods or computational machinery; providing critical feedback on methods or results or interpretation; drafting the manuscript or revising it critically for important intellectual content; and managing the publication process.

## Data sharing statement

To download the data used in these analyses and corresponding results, please visit the Global Health Data Exchange at https://ghdx.healthdata.org/record/ihme-data/gbd-2023-demographics-1950-2023.

## Editor note

“The Lancet Group takes a neutral position with respect to territorial claims in published maps and institutional affiliations.”

## Declaration of interests

S Bhaskar reports grants or contracts from Japan Society for the Promotion of Science (JSPS), Japanese Ministry of Education, Culture, Sports, Science and Technology (MEXT) for Grant-in-Aid for Scientific Research (KAKENHI) (Grant ID: 23KF0126), and from JSPS and the Australian Academy of Science for JSPS International Fellowship (Grant ID: P23712); Payment or honoraria for lectures, presentations, speakers bureaus, manuscript writing or educational events from Japan Stroke Society, Japan for an invited Keynote Presentation at the 50th Annual Meeting of the Japan Stroke Society, Osaka, and from Semey Medical University, Kazakhstan for an invited Professorship and Teaching Course in Neurocardiovascular Therapy, Semey Medical University, Kazakhstan; Support for attending meetings and/or travel from Japan Stroke Society, Japan for a Travel Award for Invited Keynote Presentation at the 50th Annual Meeting of the Japan Stroke Society, Osaka; Leadership or fiduciary role in other board, society, committee or advocacy group, paid or unpaid, with the Rotary District 9675, Sydney, Australia, Global Health & Migration Hub Community, Global Health Hub Germany, Berlin, Germany, PLOS One, BMC Neurology, Frontiers in Neurology, Frontiers in Stroke, Frontiers in Public Health, Journal of Ageing Research, Neurology International, Diagnostics, & BMC Medical Research Methodology, College of Reviewers, Canadian Institutes of Health Research (CIHR), Government of Canada, World Headache Society, Bengaluru, India, Cariplo Foundation, Milan, Italy, National Cerebral and Cardiovascular Center, Department of Neurology, Division of Cerebrovascular Medicine and Neurology, Suita, Osaka, Japan, Cardiff University Biobank, Cardiff, UK, Rotary Reconciliation Action Plan, and with Japan Connect, Osaka, Japan; all outside the submitted work. R Dandona reports grants or contracts from the Mariwala Health Initiative, Mumbai, India and the Indian Council of Medical, Research, India; Participation on a Data Safety Monitoring Board or Advisory Board with the Independent Programme Oversight Committee for COPE-BP (NIHR 206976) as Chair, with the Funding Committee, Global NIHR Global Health Research Groups Call 5, NIHR, UK as a member, and with Health Metrics Sciences MS Admissions committee, University of Washington, USA; all outside the submitted work. M K Kashyap reports grants or contracts from the Indian Council of Medical Research New Delhi (Grant # 5/13/55/2020/NCD-III); Payment or honoraria for lectures, presentations, speakers bureaus, manuscript writing or educational events from ILBS, New Delhi, India and Kuvempu University, Shimoga, Karnataka, India; Indian patents pending 2023-1100-3940 and 2023-1105-8515; all outside the submitted work. J Khubchandani reports grants or contracts from Merck Sharpe Dohme, Inc, National Science Foundation, USA, and NM-INBRE (NIH *P20GM103451 subgrant)*; Support for attending meetings and/or travel from NATCO: The Organisation for Donation and Transplant Professionals; all outside the submitted work. K Krishan reports non-financial support from the UGC Centre of Advanced Study, CAS II, awarded to the Department of Anthropology, and RUSA 2.0 grant provided by the Ministry of Education to Panjab University, Chandigarh, India, outside the submitted work. S K Panda reports support for their participation in the current manuscript from Siksha ‘O’ Anusandhan (Deemed to be University) through their salary; grants or contracts through File no. 17-59/2023-24/CCRH/Tech./Coll./ICMR-Diabetes/960 and File no. HS/RC/393, both outside the submitted work. V Sharma reports other financial or non-financial support from DFSS (MHA)'s research project (DFSS28(1)2019/EMR/6) at Department of Forensic Science (Formerly Institute of Forensic Science & Criminology), Panjab University, Chandigarh, India, RUSA 2.0 grant to Panjab University and PAIR grant (ANRF/PAIR/2025/000006/PAIR) to Panjab University from ANRF, outside the submitted work. J A Singh reports consulting fees from ROMTech, Atheneum, Clear view healthcare partners, Yale, Hulio, Horizon Pharmaceuticals/DINORA, ANI/Exeltis, USA Inc., Frictionless Solutions, Schipher, Crealta/Horizon, Medisys, Fidia, PK Med, Two labs Inc., Adept Field Solutions, Clinical Care options, Putnam associates, Focus forward, Navigant consulting, Spherix, MedIQ, Jupiter Life Science, UBM LLC, Trio Health, Medscape, WebMD, and Practice Point communications; the National Institutes of Health; and the American College of Rheumatology; Payment or honoraria for lectures, presentations, speakers bureaus, manuscript writing or educational events from the speaker's bureau of Simply Speaking; Support for attending meetings and/or travel as a Past steering committee member of OMERACT; Participation on a Data Safety Monitoring Board or Advisory Board from FDA Arthritis Advisory Committee; Leadership or fiduciary role in other board, society, committee or advocacy group, paid or unpaid as Past steering committee member of the OMERACT, an international organisation that develops measures for clinical trials and receives arm's length funding from 12 pharmaceutical companies; Stock or stock options from Atyr pharmaceuticals, Atai life sciences, Kintara therapeutics, Intelligent Biosolutions, Acumen pharmaceutical, TPT Global Tech, Vaxart pharmaceuticals, Atyu biopharma, Adaptimmune Therapeutics, GeoVax Labs, Pieris Pharmaceuticals, Enzolytics Inc., Seres Therapeutics, Tonix Pharmaceuticals Holding Corp., Aebona Pharmaceuticals, and Charlotte's Web Holdings, Inc, and previously owned stock options in Amarin, Viking and Moderna pharmaceuticals; outside the submitted work. Samer Singh reports grants or contracts from DTRA-Biological Threat Reduction Program, HDTRA1-21-1-0036; Indian Council of Medical Research, VIR/12/2022/ECD-1; and Institute of Eminence (IoE), Banaras Hindu University, which partially support their laboratory, all outside the submitted work. Satwinder Singh reports support for their participation in the current manuscript from the Anusandhan National Research Foundation (ANRF) under the Partnerships for Accelerated Innovation and Research (PAIR) programme under sanction order No. ANRF/PAIR/2025/000029/PAIR dated 20 September 2025 to Central University of Punjab. E Upadhyay reports patents issued for a system and method of reusable filters for anti-pollution mask, a system and method for electricity generation through crop stubble by using microbial fuel cells, a system for disposed personal protection equipment (PPE) into biofuel through pyrolysis and method, a novel herbal pharmaceutical aid for formulation of gel and method thereof, Herbal drug formulation for treating lung tissue degenerated by particulate matter exposure, and a method to transform cow dung into the wall paint by using natural materials and composition thereof, and patents filed for biodegradable packaging composition and method of preparation thereof, eco-friendly bio-shoe polish from banana and turmeric, honey-based polyherbal syrup composition to treat air pollution-induced inflammation and preparation method thereof, process for preparing a caffeine free, antioxidant and nutrient rich beverage, polyherbal oral mist composition for dual relief and method for preparation thereof, and a method for fabricating sustainable waterproof material sheets from LDPE plastic wastes; all outside the submitted work.

## Appendix A Supplementary data

Appendix 2

Collab Authors
