# Supplementary material for: Stillbirths at 22- and 28-weeks’ gestation or longer for the states in India: findings from the Global Burden of Disease Study 2023
Source: Lancet Reg Health Southeast Asia. 2026 Jul 27;52:100819. doi: 10.1016/j.lansea.2026.100819 (PMC13446360; doi:10.1016/j.lansea.2026.100819)
Supplement: Supplementary file 1 [file mmc1.docx]

1 June 2026

**Stillbirths at 22- and 28-weeks’ gestation or longer for the states in India:**

**Findings from the Global Burden of Disease Study 2023**

**Web Appendix 1**

Correspondence to: Prof. Rakhi Dandona

[rakhi.dandona@phfi.org](mailto:rakhi.dandona@phfi.org)

Table of Contents

[1. Methods	3](#_Toc223755605)

[2. Data inputs from vital statistics and surveys for stillbirth estimates for India, GBD 2023. All sources indicated below provided data for >28 weeks gestation. Bolded text in the data source indicates the geographic location.	6](#_Toc223755606)

[3.	Data inputs from scientific literature for stillbirth estimates for India, GBD 2023. All sources indicated below provided data for >28 weeks gestation and two sources in bold provided data for >22 weeks gestation as well.	7](#_Toc223755607)

[4. Geographic distribution of scientific literature utilised in the Global Burden of Disease Study 2023.	10](#_Toc223755608)

[5. Sustainable Development Goal 3 (SDG3) index score (2023-24), stillbirth rate (SBR) at ≥22 weeks’ and ≥28 weeks’ of gestation, and neonatal mortality rate (NMR) for India and its states, 2023. UI denotes uncertainty interval.	11](#_Toc223755609)

[6. Estimated number of stillbirths at ≥22 weeks’ and ≥28 weeks’ of gestation for India and its states, 2023. UI denotes uncertainty interval.	12](#_Toc223755610)

[7. State-level ratio of late gestation stillbirth rate (≥28 weeks’ gestation) to neonatal mortality rate (NMR) in the Global Burden of Disease study 2023.	13](#_Toc223755611)

[8. Point estimates for late gestation stillbirth rate (SBR) and neonatal mortality rate (NMR) from Global Burden of Disease (GBD) study and Sample Registration System (SRS) report, and the SBR ratio and NMR ratio of GBD to SRS for India and states for year 2023. NA denotes not available.	14](#_Toc223755612)

# 1. Methods

**Stillbirth rate (SBR)**

While the GBD 2021 Stillbirth analysis provides a detailed description of all analytical procedures, below is a comprehensive summary of the steps used to produce the GBD 2023 stillbirth estimates.^1^

This analysis produced estimates for three gestational age thresholds of stillbirth, where 20 weeks’ or longer was the primary model because it accounts for the full enumeration of fetal loss. The other gestational age thresholds estimated were 22 weeks’ or longer (the ICD-11 threshold) and 28 weeks’ or longer (the WHO benchmarking threshold).^2^

Data seeking for this analysis utilised the Global Health Data Exchange (GHDx) as well as PubMed for recently published scientific literature articles. During the GHDx cataloguing process, data sources are assigned relevant keywords. We began by compiling a filtered list of all data sources on the GHDx tagged with the “stillbirth” keyword. We also included all sources from the GBD 2016 dataset in our review. Each data source was thoroughly reviewed, and extraction occurred if the source reported the necessary stillbirth variables. Whenever possible, we preferred to extract stillbirth counts along with corresponding live birth counts or total birth counts, so we could manually calculate stillbirth rate as # stillbirths / # total births. In cases where live births or total births were not reported, we calculated the missing variable using the equation stillbirths + live births = total births. Otherwise, we extracted stillbirth rates directly from the report.

Annual data corresponding to any of the following categorisations of fetal death was extracted: completed gestation of 20 weeks’ or longer, 22 weeks’ or longer, 24 weeks’ or longer, 26 weeks’ or longer, or 28 weeks’ or longer; and birthweight of 500 g or greater and 1000 g or greater. Data with a combination of criteria (≥22 weeks’ or ≥500 g, ≥28 weeks’ or ≥1000 g, ≥22 weeks’ and ≥500 g, and ≥28 weeks’ and ≥1000 g) were also included. If multiple definitions were available, each was extracted as a separate observation. For about 20% of reported observations, no case definition could be found, so these observations were manually assigned a definition (generally 28 weeks’ or longer or 22 weeks’ or longer) based on comparisons to their closest neighbours in time and space. In total, for the GBD 2023 stillbirth analysis, stillbirth data was extracted from 12 143 (11 412 in GBD 2021) sources, including 249 surveys, 251 published studies, 1667 vital statistics reports, and for 10 381 unique location-year combinations of vital registration. For India specifically, we used 122 sources, comprised of surveys, scientific literature, vital statistics reports, and sample registration reports.

The details of data seeking, usage considerations, and potential biases for vital registration data, household surveys, and published scientific literature are described in detail elsewhere.^1^

Adjustments were made to surveys reporting only period incidence of stillbirths by applying the ratio of the number of women reporting a birth in the previous 5 years over the total number of births observed among this group to the reported SBR, and all SBR data was adjusted for completeness using completeness estimates specific to source type, location, and year from the GBD 2023 mortality analysis.^3^ To calculate the ratio of SBR to NMR, each observation of SBR was matched to a GBD 2023 NMR estimate from the same location-year. We applied a specific set of outliering criteria to exclude observations considered implausible: 1) SBR less than 1 (per 1000 births), 2) SBR greater than 200 (per 1000 births), 3) SBR greater than 50 (per 1000 births) for the high-income GBD super-region, 4) observations with a major mortality shock in the location-year (i.e. if the with-shock death rate among all ages of the population was more than 500 per 100 000 people), and 5) an SBR to NMR ratio less than 0·5.

Then, using statistical crosswalk models, data was standardised to each gestational age threshold (>22 or ≥28 weeks’), so the implied value of a data point if it were to meet the specific threshold was imputed, thereby making it comparable to all other data points. Another round of systematic outliering was performed on the new dataset with imputed values using the same criteria listed above, and deduplication was performed to 1) remove sources where multiple different definitions were extracted and 2) ensure vital registration was used to inform the model over vital statistics reports for location-years with data extracted from both.

SBR to NMR ratios were estimated using a three-stage modelling process comprised of an ensemble linear mixed-effects model (stage 1), spatiotemporal smoothing (ST; stage 2), and Gaussian process regression (GPR; stage 3). For the stage 1 model, we ran models using every combination of 10 specified covariates from the GBD 2023 cause-specific mortality models of neonatal and maternal disorders: (1) the proportion of births with 1+ antenatal care visits, 2) the proportion of births with 4+ antenatal care visits, 3) in-facility delivery, 4) skilled birth attendance, 5) maternal care and immunisation (composite metric of vaccine and maternal care coverage), 6) Healthcare Access and Quality (HAQ) Index, 7) Socio-demographic Index (SDI; composite metric of total fertility rate among those aged <25 years, mean years of education for those aged ≥15 years, and per-capita income), 8) maternal education (years per capita), 9) population with at least 12 years of education (among women of reproductive age), and 10) education relative inequality (Gini coefficient)), and then the top 50 highest-ranking ones based on out-of-sample predictive validity with statistically significant beta coefficients in the specified direction were combined into an ensemble. The stage 2 model, ST, further informed the estimates using hyperparameters for time and space that were generated based on data density. The stage 3 model, GPR, incorporated the data and its variance, scale and amplitude parameters, and a prior to generate final estimates of the SBR to NMR ratio for each location-year. Uncertainty was derived by generating 1000 draws of the SBR to NMR ratio for each location. The means of these draw-level estimates were used as the final estimates, and 95% uncertainty intervals (UIs) were assigned as the 2.5^th^ and 97.5^th^ percentiles. Lastly, the GBD 2023 NMR estimates were applied to calculate SBR, and stillbirth counts were also produced. A complete set of results can be found at <https://ghdx.healthdata.org/record/ihme-data/gbd-2023-demographics-1950-2023>.

**Neonatal mortality rate (NMR)**

The GBD 2023 all-cause mortality analysis provides a detailed description of all analytical procedures.^3^ Below, we provide a summary of the steps used to produce the GBD 2023 NMR (neonatal [<28 days] deaths per 1000 livebirths) estimates, but sex-specific and all sexes combined estimates are also presented elsewhere for 25 age groups from early neonatal (0–6 days) to 95 years and older.

All-cause mortality was informed by 24 025 data sources which can be viewed online in the Global Health Data Exchange’s GBD 2023 Sources Tool (https://ghdx.healthdata.org/gbd-2023/sources). Unlike GBD 2021, all data was processed in age-specific mortality rate space. These rates came from complete birth histories (CBHs) for age groups under 15 years of age, summary birth histories (SBHs) using a new age-sex splitting algorithm, sibling survival histories for ages 15-19 years to 45-49 years, household deaths reported in the last 12 months, vital registration and sample registration (also using the new age-sex splitting algorithm for aggregate age groups, when present), and 38 International Demographic Surveillance System (HDSS) sites in 15 countries.

The new mortality estimation pipeline used a novel statistical modeling tool, OneMod, to produce estimates of all-cause mortality across location, age, sex, and time using a single, stagewise model with both parametric and non-parametric components linked within an overarching statistical framework. It contained two main components: 1) SpXMod - a regression analysis with a generalised linear modelling tool that accounts for age-specific covariate effects and 2) KReg - a Kronecker-factored multivariate kernel regression model that smoothed across time and space due to its non-parametric modelling of residuals. Five covariates, known to be predictive of all-cause mortality, helped inform the model: 1) Socio-demographic Index (SDI), 2) HIV mortality rate, 3) age-standardised COVID-19 mortality rate, 4) a binary covariate indicating whether each location is an island, and 5) an all-risk-factor summary covariate calculated as the total population attributable fraction (PAF) for all risk factors combined for age-standardised all-cause mortality, excluding deaths due to HIV/AIDS and deaths due to fatal discontinuities.

Using SpXMod, models were fit using datasets from 1950 onwards and 1990 onwards. Stage 1 of SpXMod fit a global age-specific covariate model with intercepts for age and location, and Stage 2 used the results as a constant offset for a global time model and then fit super-region-specific and age-specific trends and intercepts. Stage 3 then add country-specific and age-specific intercepts. For countries with subnational locations, like India, the model had a Stage 4 where subnational models were fit using the results from the national model as a constant offset. Lastly, a custom splicing step combined the estimates from the 1950 onwards and 1990 onwards models, and additional smoothing was performed. Uncertainty was obtained via a model-based asymptote and was scaled based on geographical granularity.

Life tables with single-year age groups (and additional granularity for ages under 1) were generated using the age-specific mortality rates estimated above, and fatal discontinuities were applied to generate the final life tables. Abridged life tables were then calculated to generate aggregate age groups such as the neonatal age group. These estimates constituted the final NMR estimates used in the stillbirth analysis.

**References**

1. GBD 2021 Global Stillbirths Collaborators. Global, regional, and national stillbirths at 20 weeks' gestation or longer in 204 countries and territories, 1990-2021: findings from the Global Burden of Disease Study 2021. *Lancet* 2024; **404**(10466): 1955-88.
2. World Health Organization. International Classification of Diseases, Eleventh Revision (ICD-11). 2025. https://icd.who.int/browse/2025-01/mms/en#/http%3a%2f%2fid.who.int%2ficd%2fentity%2f505744734 (accessed 25 July 2025).
3. GBD 2023 Demographics Collaborators. Global age-sex-specific all-cause mortality and life expectancy estimates for 204 countries and territories and 660 subnational locations, 1950–2023: a demographic analysis for the Global Burden of Disease Study 2023. *Lancet (London, England)* 2025; 406 (10513): 1731-810.

# **2. Data inputs from vital statistics and surveys for stillbirth estimates for India, GBD 2023. All sources indicated below provided data for >28 weeks gestation. Bolded text in the data source indicates the geographic location**.

| **Type of data source** | **Data source** | **Year(s)** |
| --- | --- | --- |
| Vital statistics | Office of the Registrar General & Census Commissioner, Ministry of Home Affairs, Government of India. **India** SRS Statistical Report. New Delhi, India: Office of the Registrar General & Census Commissioner. | 1995-2020 |
| Vital statistics | Office of the Registrar General & Census Commissioner, Ministry of Home Affairs, Government of India. **India** Vital Statistics. New Delhi, India: Office of the Registrar General and Census Commissioner. | 2010-2020 |
| Vital statistics | Office of the Registrar General & Census Commissioner, Ministry of Home Affairs, Government of India. **India** SRS Compendium of Fertility and Mortality Indicators. New Delhi, India: Office of the Registrar General & Census Commissioner. | 1971-2007 |
| Vital statistics | Directorate of Economics and Statistics and Office of Chief Registrar Births and Deaths (Delhi Territory, India). India - Delhi Registration of Births and Deaths Report. **Delhi**, India: Directorate of Economics and Statistics and Office of Chief Registrar Births and Deaths (Delhi Territory, India) | 2001, 2013-2023 |
| Vital statistics | Department of Health and Family Welfare, Government of Himachal Pradesh, Office of the Chief Registrar of Births and Deaths, Government of **Himachal Pradesh**. India - Himachal Pradesh Vital Statistics Annual Report. | 2011-2016, 2022-2023 |
| Vital statistics | Directorate of Planning, Statistics & Evaluation, Office of the Chief Registrar of Births and Deaths, Government of **Goa**. India - Annual Report on Registration of Births and Deaths. | 2022-2023 |
| Vital statistics | Office of the Registrar of Birth & Deaths and Directorate of Economics & Statistics, Karnataka, Bangalore. Annual Report on the Registration of Births and Deaths Act, 1969. **Karnataka**, India. | 2020-2022 |
| Vital statistics | Directorate of Economics and Statistics, Government of Chhattisgarh, Office of the Chief Registrar of Births and Deaths, Government of Chhattisgarh. India - Chhattisgarh Annual Vital Statistics. Raipur, India: Directorate of Economics and Statistics, Government of **Chhattisgarh**. | 2012 |
| Survey | Office of the Registrar General & Census Commissioner, Ministry of Home Affairs, Government of India. **India** Annual Health Survey Data. New Delhi, India: Office of the Registrar General & Census Commissioner | 2010-2013 |
| Survey | ICF International, International Institute for Population Sciences (India), Ministry of Health and Family Welfare (**India**). National Family Health Survey-5. | 2019-2020 |
| Survey | ICF International, International Institute for Population Sciences (India), Ministry of Health and Family Welfare (**India**). National Family Health Survey-4. | 2015-2016 |
| Survey | International Institute for Population Sciences (**India**), Macro International, Inc. National Family Health Survey-3. | 2005-2006 |
| Survey | Government of Maharashtra. India - **Maharashtra** Survey of Causes of Deaths 2014. | 2014 |

# 3. Data inputs from scientific literature for stillbirth estimates for India, GBD 2023. All sources indicated below provided data for ≥28 weeks gestation and two sources in bold provided data for ≥22 weeks gestation as well.

| **Data source** | **Year(s)** |
| --- | --- |
| Torre Monmany N, Astete JA, Ramaiah D, Suchitra J, Krauel X, Fillol M, Balasubbaiah Y, Alarcón A, Bassat Q. Extended Perinatal Mortality Audit in a Rural Hospital in India. Am J Perinatol. 2023 Mar;40(4):375-386. | 2023 |
| Dandona R, Kumar GA, Akbar M, Dora SSP, Dandona L; ENHANCE 2020 Team; ENHANCE 2020 team. Substantial increase in stillbirth rate during the COVID-19 pandemic: results from a population-based study in the Indian state of Bihar. BMJ Glob Health. 2023;8(7): e013021. PMID: 37491108; PMCID: PMC10373740. | 2020-2021 |
| Dandona R, Kumar GA, Akbar M, Bhattacharya D, Nanda P, Dandona L. Deferred and referred deliveries contribute to stillbirths in the Indian state of Bihar: results from a population-based survey of all births. BMC Med. 2019;17(1):28. PMID: 30728016; PMCID: PMC6366028. | 2019 |
| **Ebenezer ED, Londhe V, Rathore S, Benjamin S, Ross B, Jeyaseelan L, Mathews JE. Peripartum interventions resulting in reduced perinatal mortality rates, and birth asphyxia rates, over 18 years in a tertiary centre in South India: a retrospective study. BJOG. 2019;126 (Suppl 4):21-26. PMID: 31257695.** | 2019 |
| Sharma B, Prasad G, Aggarwal N, Siwatch S, Suri V, Kakkar N. Aetiology and trends of rates of stillbirth in a tertiary care hospital in the north of India over 10 years: a retrospective study. BJOG. 2019;126 (Suppl 4):14-20. PMID: 31257693. | 2019 |
| Das MK, Chaudhary C, Kaushal SK, Khanna R, Chatterji S. Impact of Neonatal Resuscitation Capacity Building of Birth Attendants on Stillbirth Rate at Public Health Facilities in Uttar Pradesh, India. Indian Pediatr. 2019;56 (5):369-373. PMID: 31102378. | 2019 |
| Saha, Samir K et al. Causes and incidence of community-acquired serious infections among young children in south Asia (ANISA): an observational cohort study. The Lancet, 2018; 392 (10142):145 – 159. PMID: 30025808; PMCID: PMC6053599. | 2018 |
| Alliance for Maternal and Newborn Health Improvement (AMANHI) mortality study group. Population-based rates, timing, and causes of maternal deaths, stillbirths, and neonatal deaths in south Asia and sub-Saharan Africa: a multi-country prospective cohort study. Lancet Glob Health. 2018; 6 (12): e1297-e1308. PMID: 30361107; PMCID: PMC6227247. | 2018 |
| Saleem S, Tikmani SS, McClure EM, Moore JL, Azam SI, Dhaded SM, Goudar SS, Garces A, Figueroa L, Marete I, Tenge C, Esamai F, Patel AB, Ali SA, Naqvi F, Mwenchanya M, Chomba E, Carlo WA, Derman RJ, Hibberd PL, Bucher S, Liechty EA, Krebs N, Michael Hambidge K, Wallace DD, Koso-Thomas M, Miodovnik M, Goldenberg RL. Trends and determinants of stillbirth in developing countries: results from the Global Network's Population-Based Birth Registry. Reprod Health. 2018;15 (Suppl 1):100. PMID: 29945647; PMCID: PMC6019981. | 2018 |
| McClure EM, Garces A, Saleem S, Moore JL, Bose CL, Esamai F, Goudar SS, Chomba E, Mwenechanya M, Pasha O, Tshefu A, Patel A, Dhaded SM, Tenge C, Marete I, Bauserman M, Sunder S, Kodkany BS, Carlo WA, Derman RJ, Hibberd PL, Liechty EA, Hambidge KM, Krebs NF, Koso-Thomas M, Miodovnik M, Wallace DD, Goldenberg RL. Global Network for Women's and Children's Health Research: probable causes of stillbirth in low- and middle-income countries using a prospectively defined classification system. BJOG. 2018, 25 (2):131-138. PMID: 28139875; PMCID: PMC5537043. | 2018 |
| Dandona R, Kumar GA, Kumar A, Singh P, George S, Akbar M, Dandona L. Identification of factors associated with stillbirth in the Indian state of Bihar using verbal autopsy: A population-based study. PLoS Med. 2017;14 (8): e1002363. PMID: 28763449; PMCID: PMC5538635. | 2017 |
| Sharma S, Sidhu H, Kaur S. Analytical study of intrauterine fetal death cases and associated maternal conditions. Int J Appl Basic Med Res. 2016; 6 (1):11-3. PMID: 26958515; PMCID: PMC4765266. | 2016 |
| Mony PK, Varghese B, Thomas T. Estimation of perinatal mortality rate for institutional births in Rajasthan state, India, using capture-recapture technique. BMJ Open. 2015;5(3):e005966. PMID: 25783418; PMCID: PMC4369003. | 2015 |
| Baruah J, Kusre G, Bora R. Pattern of Gross Congenital Malformations in a Tertiary Referral Hospital in Northeast India. Indian J Pediatr. 2015;82(10):917-22. PMID: 25633326. | 2015 |
| **Kodkany BS, Derman RJ, Honnungar NV, Tyagi NK, Goudar SS, Mastiholi SC, Moore JL, McClure EM, Sloan N, Goldenberg RL. Establishment of a Maternal Newborn Health Registry in the Belgaum District of Karnataka, India. Reprod Health. 2015;12 (Suppl 2):S3. PMID: 26062791; PMCID: PMC4464217.** | 2015 |
| Goudar SS, Goco N, Somannavar MS, Vernekar SS, Mallapur AA, Moore JL, Wallace DD, Sloan NL, Patel A, Hibberd PL, Koso-Thomas M, McClure EM, Goldenberg RL. Institutional deliveries and perinatal and neonatal mortality in Southern and Central India. Reprod Health. 2015;12 (Suppl 2): S13. PMID: 26063586; PMCID: PMC4464025. | 2015 |
| Vogel JP, Souza JP, Mori R, Morisaki N, Lumbiganon P, Laopaiboon M, Ortiz-Panozo E, Hernandez B, Pérez-Cuevas R, Roy M, Mittal S, Cecatti JG, Tunçalp Ö, Gülmezoglu AM; WHO Multicountry Survey on Maternal and Newborn Health Research Network. Maternal complications and perinatal mortality: findings of the World Health Organization Multicountry Survey on Maternal and Newborn Health. BJOG. 2014;121 (Suppl 1):76-88. PMID: 24641538. | 2014 |
| Kochar PS, Dandona R, Kumar GA, Dandona L. Population-based estimates of still birth, induced abortion and miscarriage in the Indian state of Bihar. BMC Pregnancy Childbirth. 2014; 14:413. PMID: 25514837; PMCID: PMC4300052. | 2014 |
| Saleem S, McClure EM, Goudar SS, Patel A, Esamai F, Garces A, Chomba E, Althabe F, Moore J, Kodkany B, Pasha O, Belizan J, Mayansyan A, Derman RJ, Hibberd PL, Liechty EA, Krebs NF, Hambidge KM, Buekens P, Carlo WA, Wright LL, Koso-Thomas M, Jobe AH, Goldenberg RL; Global Network Maternal Newborn Health Registry Study Investigators. A prospective study of maternal, fetal and neonatal deaths in low- and middle-income countries. Bull World Health Organ. 2014;92(8):605-12. PMID: 25177075; PMCID: PMC4147405. | 2014 |
| Bhandari N, Mazumder S, Taneja S, Sommerfelt H, Strand TA; IMNCI Evaluation Study Group. Effect of implementation of Integrated Management of Neonatal and Childhood Illness (IMNCI) programme on neonatal and infant mortality: cluster randomised controlled trial. BMJ. 2012;344: e1634. PMCID: PMC3309879. | 2012 |
| Spector JM, Agrawal P, Kodkany B, Lipsitz S, Lashoher A, Dziekan G, Bahl R, Merialdi M, Mathai M, Lemer C, Gawande A. Improving quality of care for maternal and newborn health: prospective pilot study of the WHO safe childbirth checklist program. PLoS One. 2012;7 (5): e35151. PMID: 22615733; PMCID: PMC3353951. | 2012 |
| McClure EM, Pasha O, Goudar SS, Chomba E, Garces A, Tshefu A, Althabe F, Esamai F, Patel A, Wright LL, Moore J, Kodkany BS, Belizan JM, Saleem S, Derman RJ, Carlo WA, Hambidge KM, Buekens P, Liechty EA, Bose C, Koso-Thomas M, Jobe AH, Goldenberg RL; Global Network Investigators. Epidemiology of stillbirth in low-middle income countries: a Global Network Study. Acta Obstet Gynecol Scand. 2011;90 (12):1379-85. PMID: 21916854; PMCID: PMC3412613. | 2011 |
| Williams EK, Hossain MB, Sharma RK, Kumar V, Pandey CM, Baqui AH. Birth interval and risk of stillbirth or neonatal death: findings from rural north India. J Trop Pediatr. 2008;54 (5):321-7. PMID: 18443009. | 2008 |
| Khandait DW, Ambadekar NN, Zodpey SP, Vasudeo ND. Maternal age as a risk factor for stillbirth. Indian J Public Health. 2000;44 (1):28-30. PMID: 11439856. | 2000 |
| Baqui AH, Darmstadt GL, Williams EK, Kumar V, Kiran TU, Panwar D, Srivastava VK, Ahuja R, Black RE, Santosham M. Rates, timing and causes of neonatal deaths in rural India: implications for neonatal health programmes. Bull World Health Organ. 2006;84 (9):706-13. PMID: 17128340; PMCID: PMC2627477. | 2006 |
| Bang AT, Bang RA, Reddy HM, Deshmukh MD, Baitule SB. Reduced incidence of neonatal morbidities: effect of home-based neonatal care in rural Gadchiroli, India. J Perinatol. 2005; 25 (Suppl 1): S51-61. PMID: 15791279. | 2005 |
| Biswas AB, Nandy S, Sinha RN, Das DK, Roy RN, Datta S. Status of maternal and new born care at first referral units in the state of West Bengal. Indian J Public Health. 2004;48(1):21-6. PMID: 15704722. | 2004 |
| Bang AT, Bang RA, Baitule SB, Reddy MH, Deshmukh MD. Effect of home-based neonatal care and management of sepsis on neonatal mortality: field trial in rural India. Lancet. 1999;354(9194):1955-61. PMID: 10622298. | 1999 |
| Bai NS, Mathews E, Nair PM, Sabarinathan K, Harikumar C. Perinatal mortality rate in a south Indian population. J Indian Med Assoc. 1991;89(4):97-8. PMID: 1940405. | 1991 |

# 4. Geographic distribution of scientific literature utilised in the Global Burden of Disease Study 2023.

# 5. Sustainable Development Goal 3 (SDG3) index score (2023-24), stillbirth rate (SBR) at ≥22 weeks’ and ≥28 weeks’ of gestation, and neonatal mortality rate (NMR) for India and its states, 2023. UI denotes uncertainty interval.

|  | **SDG 3 index score** | **SBR ≥22 weeks’ per 1,000 births**  **(95% UI)** | **SBR ≥28 weeks’ per 1,000 births**  **(95% UI)** | **NMR per 1,000 livebirths**  **(95% UI)** |
| --- | --- | --- | --- | --- |
| **India** | 77 | 25.9 (21.3-32.0) | 16.1 (13.2-19.8) | 19.2 (16.5-22.3) |
| ***Empowered action group (EAG) states*** |  |  |  |  |
| Bihar | 67 | 29.5 (25.7-33.7) | 18.5 (16.1-21.2) | 22.4 (18.1-27.7) |
| Chhattisgarh | 56 | 26.1 (18.9-35.1) | 16.8 (12.2-22.6) | 24.9 (20.1-30.6) |
| Jharkhand | 77 | 28.1 (20.4-37.8) | 17.2 (12.4-23.0) | 18.9 (15.2-23.2) |
| Madhya Pradesh | 56 | 29.9 (20.8-43.1) | 18.8 (13.1-27.1) | 22.4 (18.1-27.1) |
| Odisha | 73 | 25.1 (18.3-33.6) | 15.8 (11.5-21.0) | 21.3 (17.4-25.8) |
| Rajasthan | 73 | 22.2 (16.2-29.7) | 13.6 (9.9-18.2) | 19.8 (16.3-24.1) |
| Uttar Pradesh | 61 | 38.2 (29.0-50.8) | 23.6 (18.0-31.5) | 26.1 (22.2-30.7) |
| Uttarakhand | 84 | 21.7 (15.9-29.0) | 13.1 (9.6-17.6) | 13.4 (10.5-16.6) |
| ***North-east states*** |  |  |  |  |
| Arunachal Pradesh | 70 | 13.9 (10.1-18.7) | 8.6 (6.3-11.6) | 9.6 (7.5-12.2) |
| Assam | 60 | 16.4 (11.9-22.8) | 10.2 (7.4-14.1) | 16.4 (13.2-20.2) |
| Manipur | 75 | 12.6 (9.1-16.9) | 7.9 (5.7-10.6) | 9.4 (7.4-11.8) |
| Meghalaya | 71 | 13.3 (9.7-17.9) | 8.3 (6.0-11.1) | 11.9 (9.4-15.0) |
| Mizoram | 69 | 9.3 (6.8-12.6) | 6.0 (4.4-8.1) | 8.4 (6.7-10.5) |
| Nagaland | 63 | 15.2 (11.0-20.4) | 9.4 (6.8-12.6) | 13.0 (10.4-16.3) |
| Sikkim | 66 | 15.9 (11.6-21.4) | 10.3 (7.5-13.8) | 12.1 (9.7-14.9) |
| Tripura | 79 | 21.3 (16.4-27.3) | 13.2 (10.1-16.9) | 15.6 (12.3-19.4) |
| ***Other states*** |  |  |  |  |
| Andhra Pradesh | 78 | 21.4 (15.5-28.7) | 13.3 (9.7-17.9) | 15.3 (12.1-19.1) |
| Delhi | 93 | 15.4 (11.3-20.5) | 9.0 (6.6-12.0) | 11.0 (8.9-13.3) |
| Goa | 79 | 14.4 (10.7-19.7) | 8.4 (6.3-11.5) | 9.6 (7.7-12.0) |
| Gujarat | 90 | 20.8 (15.8-27.8) | 13.0 (9.9-17.4) | 16.1 (13.0-19.6) |
| Haryana | 73 | 20.4 (16.0-25.9) | 12.3 (9.7-15.7) | 16.1 (12.8-19.6) |
| Himachal Pradesh | 83 | 11.3 (8.5-14.8) | 6.8 (5.1-9.0) | 10.7 (8.4-13.2) |
| Jammu & Kashmir and Ladakh | 78 | 20.4 (14.8-27.5) | 12.6 (9.2-17.0) | 14.6 (11.7-17.9) |
| Karnataka | 80 | 13.0 (9.4-17.4) | 8.0 (5.8-10.8) | 10.3 (8.4-12.7) |
| Kerala | 80 | 11.2 (8.1-15.0) | 6.8 (4.9-9.1) | 4.4 (3.5-5.5) |
| Maharashtra | 84 | 15.7 (12.4-19.8) | 9.5 (7.5-12.0) | 12.2 (9.7-15.1) |
| Punjab | 79 | 22.9 (16.6-30.8) | 14.1 (10.2-18.9) | 14.4 (11.5-17.6) |
| Tamil Nadu | 77 | 11.3 (8.2-15.2) | 6.9 (5.0-9.3) | 9.5 (7.4-11.8) |
| Telangana | 73 | 18.3 (13.3-24.6) | 11.5 (8.3-15.4) | 14.3 (11.4-17.9) |
| West Bengal | 79 | 21.1 (15.3-28.3) | 13.1 (9.5-17.5) | 14.1 (11.2-17.3) |
| Other Union Territories | 69 | 17.5 (12.2-25.3) | 10.7 (7.5-15.4) | 12.4 (9.8-15.2) |

# 6. Estimated number of stillbirths at ≥22 weeks’ and ≥28 weeks’ of gestation for India and its states, 2023. UI denotes uncertainty interval.

|  | **Estimated number of stillbirths** | |
| --- | --- | --- |
|  | **≥22 weeks’ gestation**  **(95% UI)** | **≥28 weeks’ gestation**  **(95% UI)** |
| **India** | 565,900 (462,600-702,900) | 347,300 (284,600-430,000) |
| ***EAG states*** |  |  |
| Bihar | 77,300 (67,000-88,800) | 48,000 (41,700-55,100) |
| Chhattisgarh | 15,000 (10,800-20,400) | 9,600 (6,900-12,900) |
| Jharkhand | 19,800 (14,200-26,900) | 11,900 (8,600-16,100) |
| Madhya Pradesh | 48,400 (33,300-70,700) | 30,100 (20,800-43,700) |
| Odisha | 16,400 (11,900-22,100) | 10,200 (7,400-13,700) |
| Rajasthan | 34,400 (24,900-46,400) | 20,900 (15,200-28,000) |
| Uttar Pradesh | 202,500 (152,300-272,700) | 123,600 (93,400-165,700) |
| Uttarakhand | 3,400 (2,500-4,600) | 2,100 (1,500-2,800) |
| ***North-east states*** |  |  |
| Arunachal Pradesh | 400 (300-500) | 250 (200-300) |
| Assam | 9,800 (7,100-13,700) | 6,000 (4,400-8,400) |
| Manipur | 600 (400-800) | 400 (300-500) |
| Meghalaya | 800 (500-1,000) | 500 (300-600) |
| Mizoram | 100 (90-200) | 80 (60-100) |
| Nagaland | 300 (200-400) | 200 (100-300) |
| Sikkim | 150 (100-200) | 90 (60-100) |
| Tripura | 900 (700-1,100) | 500 (400-700) |
| ***Other states*** |  |  |
| Andhra Pradesh | 13,600 (9,800-18,400) | 8,400 (6,100-11,400) |
| Delhi | 3,500 (2,500-4,600) | 2,000 (1,500-2,700) |
| Goa | 120 (90-200) | 70 (50-100) |
| Gujarat | 20,700 (15,600-27,900) | 12,800 (9,700-17,300) |
| Haryana | 8,300 (6,500-10,600) | 5,000 (3,900-6,300) |
| Himachal Pradesh | 800 (6,00-1,100) | 500 (400-700) |
| Jammu & Kashmir and Ladakh | 3,200 (2,300-4,400) | 2,000(1,400-2,700) |
| Karnataka | 12,200 (8,800-16,500) | 7,500 (5,400-10,100) |
| Kerala | 4,200 (3,000-5,700) | 2,500 (1,800-3,400) |
| Maharashtra | 20,900 (16,500-26,600) | 12,500 (9,900-15,900) |
| Punjab | 7,200 (5,100-9,700) | 4,400 (3,100-5,900) |
| Tamil Nadu | 8,900 (6,400-12,000) | 5,400 (3,900-7,300) |
| Telangana | 9,500 (6,900-12,900) | 5,900 (4,300-8,000) |
| West Bengal | 21,700 (15,600-29,400) | 13,300 (9,600-18,000) |
| Other Union Territories | 500 (400-800) | 300 (200-500) |

# 7. State-level ratio of late gestation stillbirth rate (≥28 weeks’ gestation) to neonatal mortality rate (NMR) in the Global Burden of Disease study 2023.

SBR (≥28 weeks’) to NMR ratio

| State (SDG index score) | SBR (≥28 weeks) to NMR ratio |
| --- | --- |
| Assam (60) | 0.62 |
| Himachal Pradesh (83) | 0.64 |
| Chhattisgarh (56) | 0.67 |
| Rajasthan (73) | 0.69 |
| Meghalaya (71) | 0.7 |
| Mizoram (69) | 0.71 |
| Nagaland (63) | 0.72 |
| Tamil Nadu (77) | 0.73 |
| Odisha (73) | 0.74 |
| Haryana (73) | 0.76 |
| Maharashtra (84) | 0.78 |
| Karnataka (80) | 0.78 |
| Telangana (73) | 0.8 |
| Gujarat (90) | 0.81 |
| Delhi (93) | 0.82 |
| Bihar (67) | 0.83 |
| Manipur (75) | 0.84 |
| Madhya Pradesh (56) | 0.84 |
| India (77) | 0.84 |
| Tripura (79) | 0.85 |
| Sikkim (66) | 0.85 |
| Other Union Territories (69) | 0.86 |
| Jammu & Kashmir and Ladakh (78) | 0.86 |
| Andhra Pradesh (78) | 0.87 |
| Goa (79) | 0.88 |
| Arunachal Pradesh (70) | 0.9 |
| Uttar Pradesh (61) | 0.91 |
| Jharkhand (77) | 0.91 |
| West Bengal (79) | 0.93 |
| Uttarakhand (84) | 0.98 |
| Punjab (79) | 0.98 |
| Kerala (80) | 1.55 |

# 8. Point estimates for late gestation stillbirth rate (SBR) and neonatal mortality rate (NMR) from Global Burden of Disease (GBD) study and Sample Registration System (SRS) report, and the SBR ratio and NMR ratio of GBD to SRS for India and states for year 2023. NA denotes not available.

|  | **Late gestation SBR per 1,000 births** | | **GBD to SRS SBR ratio** | **NMR per 1,000 livebirths** | | **GBD to SRS NMR ratio** |
| --- | --- | --- | --- | --- | --- | --- |
|  | **GBD 2023** | **SRS 2023** |  | **GBD 2023** | **SRS 2023** |  |
| **India** | 16.1 | 7 | 2.3 | 19.2 | 19.0 | 1.0 |
| ***EAG states*** |  |  |  |  |  |  |
| Bihar | 18.5 | 4 | 4.6 | 22.4 | 18.0 | 1.2 |
| Chhattisgarh | 16.8 | 9 | 1.9 | 24.9 | 26.0 | 1.0 |
| Jharkhand | 17.2 | 7 | 2.5 | 18.9 | 20.0 | 0.9 |
| Madhya Pradesh | 18.8 | 7 | 2.7 | 22.4 | 27.0 | 0.8 |
| Odisha | 15.8 | 16 | 1.0 | 21.3 | 21.0 | 1.0 |
| Rajasthan | 13.6 | 6 | 2.3 | 19.8 | 21.0 | 0.9 |
| Uttar Pradesh | 23.6 | 12 | 2.0 | 26.1 | 26.0 | 1.0 |
| Uttarakhand | 9.6 | 8 | 1.2 | 13.4 | 14.0 | 1.0 |
| ***North-east states*** |  |  |  |  |  |  |
| Arunachal Pradesh | 8.6 | NA | NA | 9.6 | NA | NA |
| Assam | 10.2 | 6 | 1.7 | 16.4 | 21.0 | 0.8 |
| Manipur | 7.9 | NA | NA | 9.4 | NA | NA |
| Meghalaya | 8.3 | NA | NA | 11.9 | NA | NA |
| Mizoram | 6.0 | NA | NA | 8.4 | NA | NA |
| Nagaland | 9.4 | NA | NA | 13.0 | NA | NA |
| Sikkim | 10.3 | NA | NA | 12.1 | NA | NA |
| Tripura | 13.2 | NA | NA | 15.6 | NA | NA |
| ***Other states*** |  |  |  |  |  |  |
| Andhra Pradesh | 13.3 | 2 | 4.4 | 15.3 | 16.0 | 1.0 |
| Delhi | 9.0 | 4 | 2.3 | 11.0 | 9.0 | 1.2 |
| Goa | 8.4 | NA | NA | 9.6 | NA | NA |
| Gujarat | 13.0 | 5 | 2.6 | 16.1 | 15.0 | 1.1 |
| Haryana | 12.3 | 9 | 1.4 | 16.1 | 19.0 | 0.8 |
| Himachal Pradesh | 6.8 | 4 | 1.7 | 10.7 | 11.0 | 1.0 |
| Jammu & Kashmir and Ladakh | 12.6 | 4 | 3.2 | 14.6 | 10.0 | 1.5 |
| Karnataka | 8.0 | 8 | 1.0 | 10.3 | 11.0 | 0.9 |
| Kerala | 6.8 | 5 | 1.4 | 4.4 | 4.0 | 1.1 |
| Maharashtra | 9.5 | 5 | 1.9 | 12.2 | 11.0 | 1.1 |
| Punjab | 14.1 | 5 | 2.8 | 14.4 | 12.0 | 1.2 |
| Tamil Nadu | 6.9 | 4 | 1.7 | 9.5 | 9.0 | 1.1 |
| Telangana | 11.5 | 3 | 3.8 | 14.3 | 14.0 | 1.0 |
| West Bengal | 13.1 | 6 | 2.2 | 14.1 | 13.0 | 1.1 |
| Other Union Territories | 10.7 | NA | NA | 12.4 | NA | NA |
