# Supplementary material for: Stillbirths at 22- and 28-weeks’ gestation or longer for the states in India: findings from the Global Burden of Disease Study 2023
Source: Lancet Reg Health Southeast Asia. 2026 Jul 27;52:100819. doi: 10.1016/j.lansea.2026.100819 (PMC13446360; doi:10.1016/j.lansea.2026.100819)
Supplement: Supplementary file 2 [file mmc2.docx]

# Appendix 2: Authorship appendix to “Stillbirths at 22- and 28-weeks’ gestation or longer for the states in India: Findings from the Global Burden of Disease Study 2023”

This appendix provides further authorship detail for “Stillbirths at 22- and 28-weeks’ gestation or longer for the states in India: Findings from the Global Burden of Disease Study 2023”

Table of Contents

[India State-Level Disease Burden Initiative Stillbirths Collaborators	2](#_Toc231208436)

[Affiliations	4](#_Toc231208437)

[Authors’ Contributions	14](#_Toc231208438)

[Writing the first draft of the manuscript	14](#_Toc231208439)

[Providing data or critical feedback on data sources	14](#_Toc231208440)

[Developing methods or computational machinery	15](#_Toc231208441)

[Providing critical feedback on methods or results or interpretation	15](#_Toc231208442)

[Drafting the work or revising it critically for important intellectual content	17](#_Toc231208443)

[Managing the publications process	19](#_Toc231208444)

## India State-Level Disease Burden Initiative Stillbirths Collaborators

Rakhi Dandona, G Anil Kumar, Haley Comfort, Theresa A McHugh, Bhoomadevi A, Rizwan Suliankatchi Abdulkader, Anirudh Balakrishna Acharya, Gaurav Acharya, Swetha Acharya, Usha Adiga, Mohd Adnan, Dhiraj Motilal Agarwal, Vaishali Aggarwal, Aqeel Ahmad, Danish Ahmad, Naved Ahmad, Saheem Ahmad, Mehrunnisha Sharif Ahmed, Saif Ahmed, Syed Anees Ahmed, Mohd Akbar, Mohammad Jahoor Alam, Irfan Ali, Mohammad Daud Ali, Rafat Ali, Margret Beaula Alocious Sukumar, AH Sruthi Anil Kumar, Prapti Anjana, Kabilan Annadurai, Sadaf Anwar, Razique Anwer, Gururaj Arakeri, Jesu Arockiaraj, Syed Mohammed Basheeruddin Asdaq, Mohammad Saquib Ashraf, Syed Amir Ashraf, Ashish Awasthi, Sheeba B, Prasad Hanmantrao Babar, Giridhara Rathnaiah Babu, Raju Balaji, Senthilkumar Balakrishnan, Altaf Bandy, Bhavik Bansal, Hansi Bansal, Narasimha M Beeraka, Gulam Saidunnisa Begum, Shahina Begum, Samir Bele, Uzma Iqbal Belgaumi, Om Prakash Prakash Bera, Ajeet Singh Bhadoria, Anuradha Bhardwaj, Nikha Bhardwaj, Pankaj Bhardwaj, Sonu Bhaskar, Shahnawaz Ali Bhat, Arushee Bhatnagar, Priyadarshini Bhattacharjee, Sudip Bhattacharya, Gurjit Kaur Bhatti, Jasvinder Singh Bhatti, Bijit Biswas, Monirujjaman Biswas, Trupti Bodhare, Jyoti Brahmaiah, Sravana Deepthi C, Sanjay C J, Joshua Chadwick, Rama Mohan Chandika, Baskaran Chandrasekaran, Vijay Kumar Chattu, Anis Ahmad Chaudhary, Sirshendu Chaudhuri, Waseem Chauhan, Akhilanand Chaurasia, Snehal Prakash Chavhan, Saravanan Chinnaiyan, Shivani Chopra, Devasahayam J Christopher, Mayank Dalakoti, P Vineeth Daniel, Roy Arokiam Daniel, Aso Mohammad Darwesh, Dhruva Dave, Vinoth Gnana Chellaiyan Devanbu, Devananda Devegowda, Sagnik Dey, Arkadeep Dhali, Kuldeep Dhama, Amol S Dhane, Redolen Rose Dhar, Winniecia Dkhar, S Siva Prasad Dora, Ojas Prakashbhai Doshi, Rajkumar Prakashbhai Doshi, Viola Savy Dsouza, Siddhartha Dutta, Sulagna Dutta, Venkata Ravikanth Eddula, Balisa Mosisa Ejeta, Devaraj Ezhilarasan, Mohammad Fareed, Aisha Farhana, Rohit Ganduboina, Balasankar Ganesan, Balasubramanian Ganesh, Ezhilkugan Ganessane, Shivaprakash Gangachannaiah, Vinod Kumar Garg, Rupesh K Gautam, Vikram Jeet Singh Gill, Bikash Ranjan Giri, Archit Goel, Rajesh Kumar Goel, Mahaveer Golechha, Sameer Vali Gopalani, Amit Gulati, Sheffali Gulati, Anish Kumar Gupta, Lalit Gupta, Abdul Hafiz, Pritam Halder, Mohd Anul Haq, Shamimul Hasan, Md Saquib Hasnain, Md Imtaiyaz Hassan, Omed Hassan Ahmed, Simon I Hay, Ramesh Holla, Mehdi Hosseinzadeh, MD Talib Hussain, Mohammad Tarique Imam, Mohd Imran, Leeberk Raja Inbaraj, Danish Iqbal, CV Irshad, Mustafa Alhaji Isa, Jaison Jacob, Vennila Jaganathan, Qazi Mohammad Sajid Jamal, Jerin James, Rajiv Janardhanan, Krishna Mahendrabhai Jasani, Shubha Jayaram, Felix K Jebasingh, Bijay Mukesh Jeswani, Jobin Jose, Jobinse Jose, Nitin Joseph, Krupal Joshi, Hema Shree K, Vidya Kadashetti, Sanjay Kalra, Mehnaz Kamal, Rajesh Kamath, Suthanthira Kannan S, Subham Kansal, Sunil Kumar Kar, Reddy Praneeth Karnam, Faizan Zaffar Kashoo, Manoj Kumar Kashyap, Ramya Kateel, Avleen Kaur, Harleen Kaur, Sandeep Kaur, Mohd Adnan Kausar, Kanica Kaushal, Abida Khan, Maseer Khan, Mohammad Idreesh Khan, Mohd Faheem Khan, Mohd Wajid Ali Khan, Sumaiya Khan, Wasif Ali Khan, Yusuf Saleem Khan, Parisa Khoshvaght, Samira Khoshvaght, Jagdish Khubchandani, P Ratan Khuman, Sanjay Kini B, Ladli Kishore, Shivakumar KM, Archana Koul, Sindhura Lakshmi Koulmane Laxminarayana, Kewal Krishan, Bindu Krishnan, Nitya Krishnasamy, Ananya Kuanar, Mohammed Kuddus, Anit Kujur, Ashwini Kumar, Chandan Kumar, Dewesh Kumar, Jogender Kumar, Kamal Kumar, Manoj Kumar, Mukesh Kumar, Nithin Kumar, Pawan Kumar, Pradeep Kumar, Radha Kumar, Rakesh Kumar, Sandeep Kumar, Sumit Kumar, Tushar Kumar, Subramanian Kumaran, Senthil Kumaran D, Nisha Kumari, Sajeetha Kumari R, Jibin Kunjavara, Krishna Prasad Kurpad, Pramod Kumar Kushawaha, Pallavi L C, Chandrakant Lahariya, Anita Lakhani, Dharmesh Kumar Lal, Savita Lasrado, Virendra S Ligade, Surbala Devi Lourembam, Abhilash Ludhiadch, Abdul Majid, Moutushi Majumder, Bhagabat Malik, Shahid Malik, Tabarak Malik, Lokesh Manjani, Kamaruddeen Mannethodi, Sahana Manoharan, Shaista Manzoor, Vijaya Marakala, J Jenifer Florence Mary, Madhumitha Masilamani, Mansi Mathur, Medha Mathur, Jitendra Meena, Satish Melwani, Godfred Antony Menezes, Ritesh G Menezes, Giuseppe Minervini, GK Mini, Kumar Guru Mishra, Vinaytosh Mishra, Sayan Mitra, Tridip Mitra, Chaitanya Mittal, Dhruti Modi, Taj Mohammad, Omer Mohammed, Syam Mohan, Itismita Mohanty, Siba Sankar Mohanty, Shibani Mohapatra, Afrasim Moin, Ali H Mokdad, Himel Mondal, Rohith Motappa, Shiv Kumar Mudgal, Reema Mukherjee, Sumoni Mukherjee, BV Murlimanju, Satta Muthu Murugan, Sathish Muthu, Raman Muthusamy, Karikalan Nagarajan, Dhayanithi Nagarajan Balachandran, Ganesh R Naik, Gurudatta Naik, Prasad Nalabothu, Girish Nandakumar, Dhruva Nandi, Vinay Nangia, Sanghamitra Nayak, Mahesh P A, Jagadish Rao Padubidri, Dimpal Manilal Paija, Debkumar Pal, Vijayakumar Palaniswamy, Sarika Palepu, Sujogya Kumar Panda, Deepshikha Pande Katare, Anuj Kumar Pandey, Romil R Parikh, Arpit Parmar, Pragnesh Parmar, Bhargav U Umeshchandra Patel, Kunal Nitinkumar Patel, Mehali Lalitbhai Patel, Mitesh Patel, Neel Navinkumar Patel, Niki Rut Patel, Sangram Kishor Patel, Satyananda Patel, Shivani Patel, Ashlesh Patil, Sandip Shripati Patil, Shankargouda Patil, Sourav S Patnaik, Mohamed Anas Patni, Apurba Patra, Shrikant Pawar, Arun Mitra Peddireddy, Bala Ganesh Pichamuthu, Ramesh Poluru, Akila Prashant, Pooja Puri, Surabhi Puri, Manas Pustake, Shuby Puthussery, Jagadeesh Puvvula, Shahazad Niwazi Qurashi, Safia Obaidur Rab, Navid Rabiee, Venkatraman Radhakrishnan, Yashpal Singh Raghav, Pracheth Raghuveer, Mohammad Hifz Ur Rahman, Amita Rai, Sunil Kumar Raina, Jeffrey Pradeep Raj, Gunaseelan Rajendran, Judah Rajendran, Pushp Lata Rajpoot, Prashant Rajput, Chitra Ramasamy, Shakthi Kumaran Ramasamy, Balaji Ramraj, Lokesh Rana, Chythra R Rao, Priyanka Rao, Rajath Rao, Suchetha S Rao, Shree Rath, Isha Rathi, Lokesh Ravi, Ramya Kundayi Ravi, Renju Ravi, Karthik Rayapureddi, Bhagwan Narayan Rekadwad, Moattar Raza Rizvi, Syed Mohd Danish Rizvi, Hannah Elizabeth Robinson-Oden, Ravi Rohilla, Himanshu Sekhar Rout, Parameswari Royapuram Parthasarathy, Manjula S, Chandan S N, Mohd Saeed, Rajesh Sagar, Ashok Kumar Sah, Indranil Saha, Amirhossein Sahebkar, Vaibhav Sahni, Ambika Sahoo, Harihar Sahoo, Soumya Swaroop Sahoo, Binaya Krushna Sahu, Kirti Sundar Sahu, Mahesh Chandra Sahu, Sandeep G Sangle, Rama Krishna Sanjeev, Sathish Sankar, Senthilkumar Sankararaman, Prasanna K Santhekadur, Sivan Yegnanarayana Iyer Saraswathy, Siddaram Shivaji Sarate, Aswini Saravanan, Sachin C Sarode, Mukesh Kumar Sathya Narayanan, Maheswar Satpathy, Shakta Mani Satyam, Jennifer Saulam, Ganesh Kumar Saya, Durairaj Sekar, Siddharthan Selvaraj, Vimalraj Selvaraj, Pallav Sengupta, Subramanian Senthilkumaran, Muhammed Shabil, Disha Sanjivkumar Shah, Shipraa Arpan Shah, Sweni Shah, Uzma Shahab, Syed Monowar Alam Shahid, Muhammad Aaqib Shamim, Mohd Shanawaz, Devika Shanmugasundaram, Mohammed Shannawaz, Amit Baran Sharangi, Ankita Sharma, Arkansh Sharma, Avimanu Sharma, Bhoopesh Kumar Sharma, Bunty Sharma, Dolly Sharma, Kamlesh Sharma, Manoj Sharma, Ravi Kumar Sharma, Ujjawal Sharma, Vishal Sharma, Rekha Raghuveer Shenoy, Subuhi Sherwani, Adithi Shetty, B Suresh Kumar Shetty, Mahabalesh Shetty, Premalatha K Shetty, Shiran Shetty, Suraj S Shetty, Arun Shirali, Priyanka Arun Shirali, Dharmsheel Shrivastav, Samra Siddiqui, Akanksha Singh, Amit Singh, Baljinder Singh, Brij Pal Singh, Harmanjit Singh, Harpreet Singh, Jasvinder A Singh, Jawahar Singh, Kalpana Singh, Mitasha Singh, Paramdeep Singh, Poornima Suryanath Singh, Puneetpal Singh, Samer Singh, Satwinder Singh, Shilpy Singh, Siddharth Singh, Surendra Singh, Vanshika Singh, Yadvinder Singh, Abhijeet Prasad Sinha, Mukesh Kumar Sinha, Indra Sivakumar, Farrukh Sobia, Suha Soni, Prashant Sood, Chandrashekhar T Sreeramareddy, Kannan Sridharan, Vignes Anand Srinivasalu, Shyamkumar Sriram, Vipul Srivastava, Vetriselvan Subramaniyan, Meenakshi Sundari Subramaniyan Natarajan, Alisha Suhag, Thanigaivel Sundaram, Chandan Kumar Swain, Shreya P Swami, Azharuddin Sajid Syed Khaja, Jabeen Taiba, Jay Tewari, Lakshmi Thangavelu, Kavumpurathu Raman Thankappan, Rekha Thapar, Suresh Thareja, Mahalakshmi Thayumana Sundaram, Jeevarathinam Thirumalai, Muthu Thiruvengadam, Rekha Thiruvengadam, Nihal Thomas, Nikhil Kenny Thomas, Vikas Kumar Tiwari, Marcos Roberto Tovani-Palone, Santhosh Kumar Tumkur Narayanappa, Shadab Uddin, Srikanth Umakanthan, Bhaskaran Unnikrishnan, Era Upadhyay, Dipan Uppal, Rakesh V R, Asokan Govindaraj Vaithinathan, Sai Mahesh Vajjala, Joe Varghese, Pavani Varma, Sampara Vasishta, Vishnu Priya Veeraraghavan, Anoop Velayudhan, U Venkatesh, Baskar Venkidasamy, Akshaya Kumar Verma, Poonam Verma, Ramesh Vidavalur, Sharath Chaitanya Vipparthy, Athul Vivek, Hai Nam Vu, Megha Walia, Tanveer A A Wani, Dharmveer Yadav, Suresh Yadav, Vikas Yadav, Saba Yahoo, S Yogeshkumar, Ghazala Yunus, Seema Zargar, Sanjay Zodpey, Christopher J L Murray, Lalit Dandona, Austin E Schumacher, and Nicholas J Kassebaum.

## Affiliations

Indian Institute of Public Health-Delhi (S Kaur PhD), Public Health Foundation of India, New Delhi, India (Prof R Dandona PhD, G Kumar PhD, M Akbar MA, S S P Dora MPhil, M Majumder MPhil, Prof S Zodpey PhD, Prof L Dandona MD); Institute for Health Metrics and Evaluation (Prof R Dandona PhD, H Comfort MPH, T A McHugh PhD, Prof S I Hay DSc, Prof A H Mokdad PhD, H E Robinson-Oden MLIS, H N Vu MPS, Prof C J L Murray DPhil, Prof L Dandona MD, A E Schumacher PhD, N J Kassebaum MD), Department of Health Metrics Sciences, School of Medicine (Prof R Dandona PhD, Prof S I Hay DSc, Prof A H Mokdad PhD, Prof C J L Murray DPhil, N J Kassebaum MD), Department of Anesthesiology & Pain Medicine (N J Kassebaum MD), University of Washington, Seattle, WA, USA; Amity Institute of Public Health and Hospital Administration (Prof B A PhD, A Koul MHA), Amity University Noida, Noida, India; National Institute of Epidemiology (R Abdulkader PhD), ICMR-National Institute for Research in Tuberculosis (K Nagarajan PhD), National Institute for Research in Tuberculosis (B Ramraj MD), Indian Council of Medical Research, Chennai, India; Department of Restorative Dentistry (A B Acharya PhD), College of Health Sciences (S V Gopalani PhD), Research Institute for Medical and Health Science (S Manzoor PhD), University of Sharjah, Sharjah, United Arab Emirates; Department of Anaesthesia and Critical Care (G Acharya MD), Bhopal Memorial Hospital and Research Centre, Bhopal, India; Department of Oral Pathology and Microbiology (S Acharya PhD), School of Public Health (S B MPH), Department of Biochemistry (Prof A Prashant PhD), JSS Academy of Higher Education and Research, Mysuru, India; Apollo Institute of Medical Sciences & Research Chittoor (Prof U Adiga PhD), Apollo Hospital, Chittoor, India; Department of Biology (Prof M Adnan PhD, M Alam PhD, Prof M Saeed PhD, S Sherwani PhD), Department of Biochemistry (S Anwar PhD, M A Kausar PhD, Prof M Kuddus PhD), Pharmacology and Toxicology (M Hussain PhD), Department of Chemistry (Prof M Khan PhD), Medical and Diagnostic Research Center (Prof M Khan PhD, S Sherwani PhD), College of Medicine (Y S Khan MD), Department of Health Management (R Kumar PhD), College of Pharmacy Department of Pharmaceutics (A Moin PhD), Department of Pharmaceutics (S Rizvi PhD), Department of Public Health (U Shahab PhD), Biochemistry Department (S M A Shahid PhD), Pathology (A Syed Khaja PhD), Department of Basic Science (G Yunus PhD), University of Hail, Hail, Saudi Arabia (S Siddiqui PhD); Vadu Rural Health Program (D M Agarwal PhD), KEM Hospital Research Centre, Pune, India; Department of Oncology (V Aggarwal PhD), Department of Community Medicine (P Halder MD, P Kumar PhD), Department of Pediatrics (J Kumar MD), Post Graduate Institute of Medical Education and Research, Chandigarh, India; College of Medicine (A Ahmad PhD), Department of Clinical Sciences (A Bandy MD), Shaqra University, Shaqra, Saudi Arabia; School of Medicine and Psychology (D Ahmad PhD), Australian National University, Canberra, ACT, Australia; Health Research Institute (D Ahmad PhD, I Mohanty PhD), University of Canberra, Canberra, NSW, Australia; Department of Computer Science & Information Systems (N Ahmad PhD), Department of Pharmacy Practice (Prof S Asdaq PhD), College of Medicine (M Fareed PhD), Department of Basic Medical Sciences (W Khan MD), AlMaarefa University, Riyadh, Saudi Arabia; Department of Biosciences (S Ahmad PhD), University of Hail, Hail City, Saudi Arabia; College of Nursing (M S Ahmed MSc), Department of Computer and Information Science (M Haq PhD), Department of Physical Therapy and Health Rehabilitation (Prof F Z Kashoo PhD), Majmaah University, Al Majmaah, Saudi Arabia; College of Medicine (S Ahmed MD, R Anwer PhD), Department of Biology (A A Chaudhary PhD), Imam Mohammad Ibn Saud Islamic University, Riyadh, Saudi Arabia; Brody School of Medicine (S Ahmed PhD), East Carolina University, Greenville, NC, USA; Department of Statistics and Operations Research (I Ali PhD), Department of Zoology (S Bhat PhD), Aligarh Muslim University, Aligarh, India; Department of Pharmacy (M Ali PhD), Mohammed Al-Mana College for Medical Sciences, Dammam, Saudi Arabia; Department of Biosciences (R Ali PhD), Faculty Of Dentistry (S Hasan MDS), Centre for Interdisciplinary Research in Basic Sciences (Prof M I Hassan PhD, S Khan MSc), Centre For Interdisciplinary Research In Basic Sciences (CIRBSc) (T Mohammad PhD), Jamia Millia Islamia, New Delhi, India; SRM School of Public Health, Chennai, India (M Alocious Sukumar PhD); Division of Medical Research (A Anil Kumar PhD, T Mitra MSc), Department of Obstetrics and Gynecology (S Kumari R MS), Department of General Medicine (M Subramaniyan Natarajan MD), Sri Ramaswamy Memorial Institute of Science and Technology, Chengalpattu, India; Ashok & Rita Patel Institute of Physiotherapy (P Anjana MPT), Charotar University of Science and Technology, Petlad, India; Department of Public Health (K Annadurai PhD), School of Management (R Karnam PhD), Centre for Digital Health and Precision Medicine (L Ravi PhD), The Apollo University, Chittoor, India; Department of Head and Neck Oncology (Prof G Arakeri PhD), HCG Cancer Hospital, Bengaluru, India; Department of Biotechnology (Prof J Arockiaraj PhD, T Sundaram PhD), Division of Medical Research (P Daniel PhD, R Janardhanan PhD), SRM Medical College Hospital and Research Centre (J James MD), School of Public Health (B Pichamuthu PhD), Sri Ramaswamy Memorial Institute of Science and Technology, Kattankulathur, India; Department of Basic Sciences (M Ashraf PhD), Riyadh ELM University, Riyadh, Saudi Arabia; Department of Food Technology (S Ashraf PhD), Jamia Hamdard, New Delhi, India; Department of Biostatistics (A Awasthi PhD), CSIR-Central Drug Research Institute, Lucknow, India; Department of BIostatistics (A Awasthi PhD), Academy of Scientifc and Innovative Research (AcSIR), Ghaziabad, India; Biostatistics, Epidemiology, and Data Management (BEAD) Core (P H Babar ScM), School of Medicine (B M Jeswani MBBS), Johns Hopkins University, Baltimore, MD, USA; Department of Population Medicine (Prof G Babu PhD), Qatar University, Doha, Qatar; Saveetha Medical College and Hospital (R Balaji PhD), Department of Biosciences (S Chopra MPH), Deparatment of Pharmacology, Saveetha Dental College and Hospitals (Prof D Ezhilarasan PhD), Department of Pathology (H K PhD), Department of Oral Biology (N Krishnasamy PhD), Saveetha Dental College and Hospitals (G Minervini PhD, Prof D Sekar PhD, M Tovani-Palone PhD), Department of Biochemistry (S Mohan PhD), Department of Biomaterials (N Rabiee PhD), Saveetha College of Physiotherapy (P Royapuram Parthasarathy PhD), Center for Global Health Research (Prof A Sahebkar PhD), Department of Microbiology (S Sankar PhD, I Sivakumar MSc, J Thirumalai MSc), Department of Neonatology (R Thiruvengadam PhD), Centre of Molecular Medicine and Diagnostics (Prof V Veeraraghavan PhD), Centre for Biosciences and Biotechnology (B Venkidasamy PhD), Saveetha University, Chennai, India; Division of Biological Sciences (S Balakrishnan PhD), Tamil Nadu State Council for Science and Technology, Chennai, India; Division of Cardiology (B Bansal MD), Department of Anesthesiology and Pain Management (S S Patnaik PhD), University of Texas, Dallas, TX, USA; Department of Forensic Science (H Bansal PhD), Government Institute of Forensic Science, Nagpur, Nagpur, India; Department of Human Anatomy and Histology (Prof N M Beeraka PhD), I.M. Sechenov First Moscow State Medical University, Moscow, Russia; Department of Biochemistry (Prof G S Begum MD), College of Medicine and Health Sciences (M Rahman PhD), National University of Science and Technology, Sohar, Oman; Department of Biostatistics (S Begum PhD), ICMR-National Institute for Research in Reproductive and Child Health, Mumbai, India; Community Medicine Department (S Bele MD), Central University of Tamil Nadu, Madurai, India; Department of Oral Pathology and Microbiology (U I Belgaumi MD), Department of Public Health Dentistry (Prof S KM PhD), Krishna Vishwa Vidyapeeth (Deemed to be University), Karad, India; Health Systems Strengthening (O P Bera MPH), Global Health Advocacy Incubator, Washington, DC, USA; Centre for Policy Research and Data Analytics in Health and Environment (O P Bera MPH), Department of Biochemistry (S Malik MSc), Department of Community Medicine and Family Medicine (U Venkatesh MD), All India Institute of Medical Sciences, Gorakhpur, India; Department of Community and Family Medicine (A S Bhadoria MD), All India Institute of Medical Sciences, Rishikesh, India; Community Health Department (A S Bhadoria MD), University of South Wales, South Wales, UK; Amity Institute of Public Health (A Bhardwaj PhD), Amity Institute of Forensic Sciences (P Puri PhD), Amity Institute of Public Health and Hospital Administration (I Rathi MPH, M Shannawaz PhD, A Singh PhD, P S Singh PhD), Amity University, Noida, India; Department of Anatomy (N Bhardwaj MD), Department of Pharmacology (A Saravanan MD, M Shamim MBBS), Department of Biochemistry (D Yadav PhD), All India Institute of Medical Sciences, Jodhpur, India; National Institute for Implementation Research on Non-Communicable Diseases (Prof P Bhardwaj MD, S Yadav PhD), Indian Council of Medical Research, Jodhpur, India; Global Health Neurology Lab (S Bhaskar MD), NSW Brain Clot Bank, Sydney, NSW, Australia; Division of Cerebrovascular Medicine and Neurology (S Bhaskar MD), National Cerebral and Cardiovascular Center, Suita, Japan; Family Medicine Department (A Bhatnagar MD), Texas Tech University Health Science Center (M Pustake MD), Texas Tech University, El Paso, TX, USA; Translational and Clinical Research Institute (P Bhattacharjee MD), Newcastle University, Newcastle upon Tyne, UK; Department of Community and Family Medicine (S Bhattacharya MD, B Biswas MD), Department of Physiology (H Mondal MD), College of Nursing (S Mudgal PhD), All India Institute of Medical Sciences, Deoghar, India; Department of Medical Lab Technology (Prof G K Bhatti PhD), University Centre for Research and Development (S Kalra DM), University Centre for Research and Development (UCRD) (B Singh PhD), Chandigarh University, Mohali, India; Laboratory of Translational Medicine and Nanotherapeutics (Prof J S Bhatti PhD), Central University of Punjab (Prof V K Garg PhD), Department of Microbiology (P K Kushawaha PhD, A Singh PhD), Department of Human Genetics and Molecular Medicine (U Sharma PhD), Department of Biochemistry (B Singh PhD), Department of Computer Science & Technology (Prof S Singh PhD), Department of Botany (Y Singh PhD), Central University of Punjab, Bathinda, India; Centre for the Study of Regional Development (M Biswas PhD), Jawahar Lal Nehru University, New Delhi, India; Department of Community and Family Medicine (Prof T Bodhare MD), All India Institute of Medical Sciences, Ramanathapuram, India; Department of Pathology (J Brahmaiah MD), Department of Dermatology, Venereology and Leprology (V Eddula MD), Department of Biochemistry (S Vasishta PhD), Apollo Institute of Medical Sciences and Research Chittoor, Chittoor, India; Department of Community Medicine (S D C MD), Apollo Institute of Medical Sciences and Research, Chittoor, India; JSS Dental College & Hospital (S C J MDS), Department of Respiratory Medicine (Prof M P A DNB), Department of Oral and Maxillofacial Surgery (M S MDS, C S N PhD), JSS Medical College (Prof P K Santhekadur PhD), Jagadguru Sri Shivarathreeswara Academy of Health Education and Research, Mysore, India; Non-communicable Diseases Division (J Chadwick MD), Laboratory Sciences Division (B Ganesh PhD), Division of Epidemiology & Biostatistics (D Shanmugasundaram PhD), National Institute of Epidemiology, Chennai, India; Clinical Nutrition Department (R M Chandika PhD), Epidemiology Program (M Khan MD), College of Nursing and Health Sciences (S N Qurashi PhD, M Shanawaz MD, S Uddin PhD), Department of Mathematics (Y Raghav PhD), Department of Public Health (P Rajpoot PhD, F Sobia PhD, J Varghese PhD), Department of Basic Medical Sciences (R Ravi DM), Jazan University, Jazan, Saudi Arabia; Symbiosis School of Sports Sciences (B Chandrasekaran PhD), Symbiosis International University, Pune, India; Department of Public Health and Health Sciences (Prof V Chattu PhD), Tennessee State University (TSU), Nashville, TN, USA; Department of Community Medicine (Prof V Chattu PhD), Datta Meghe Institute of Medical Sciences, Sawangi, India; Department of Public Health (S Chaudhuri MD), Indian Institute of Public Health, Hyderabad, India; Department of Physiology (W Chauhan PhD, A Majid PhD), University of Tennessee, Memphis, TN, USA; Department of Oral Medicine and Radiology (Prof A Chaurasia MD), Internal Medicine Department (V Singh MBBS), King George's Medical University, Lucknow, India; National Institute of Translational Virology and AIDS Research (S P Chavhan MD), Indian Council of Medical Research, Pune, India; SRM School of Public Health (S Chinnaiyan PhD), Department of Paediatrics (R Kumar MD), Centre for Clinical Pharmacology (M Masilamani MTech), Sri Ramaswamy Memorial Institute of Science and Technology, Chennai, India; Department of Pulmonary Medicine (Prof D J Christopher DNB), Department of Endocrinology, Diabetes and Metabolism (Prof F K Jebasingh DM, Prof N Thomas PhD), Christian Medical College and Hospital (CMC), Vellore, India; Department of Public Health and Primary Care (M Dalakoti MPH), University of Cambridge, Cambridge, UK; Cardiovascular Metabolic Translational Research Program (M Dalakoti MPH), National University of Singapore, Singapore, Singapore; Department of Community Medicine (R A Daniel MD), Employees' State Insurance Model Hospital, Chennai, India; International Center for Materials Sciences and Technology (A M Darwesh PhD, O Hassan Ahmed PhD), Western Caspian University, Baku, Azerbaijan; Jadara Research Center (A M Darwesh PhD), Jadara University, Irbid, Jordan; Division of Gynecology Oncology (D Dave MD), Department Health Services Research (G Naik MPH), University of Alabama at Birmingham, Birmingham, AL, USA; Chettinad Hospital & Research Institute (Prof V Devanbu MD), Chettinad Academy of Research and Education, Chennai, India; JSS Medical College (D Devegowda PhD), Jagadguru Sri Shivarathreeswara Academy of Health Education and Research, Mysuru, India; Centre for Atmospheric Sciences (Prof S Dey PhD), Indian Institute of Technology Delhi, New Delhi, India; School of Health, Policy and Management (Prof S Dey PhD), Korea University, Seoul, South Korea; Sheffield Teaching Hospitals NHS Foundation Trust, Sheffield, UK (A Dhali MBBS); Division of Pathology (K Dhama PhD), ICAR-Indian Veterinary Research Institute, Bareilly, India; Research and Development Cell (A S Dhane MBA), Department of Oral Pathology and Microbiology (Prof S C Sarode PhD), Dr. D. Y. Patil Vidyapeeth, Pune (Deemed to be University), Pune, India; Health Information Management (R R Dhar MPH), Department of Pharmacology (S Gangachannaiah MD, R Kateel PhD, R R Shenoy PhD), Centre for Toxicovigilance & Drug Safety (A K Gupta PharmD), Kasturba Medical College, Mangalore (R Holla MD), Prasanna School of Public Health (R Kamath MHA), Department of Community Medicine (S Kini B MD, C R Rao MD), Kasturba Medical College, Manipal (A Kumar MD, P L C MD), Department of Physiotherapy (S Kumaran D PhD, G Nandakumar PhD, M K Sinha PhD), Department of Pharmaceutical Regulatory Affairs and Management (V S Ligade PhD), Division of Clinical Pharmacology (J P Raj DM), Department of Medical Imaging Technology (P Rao PhD), Manipal College of Dental Sciences, Mangalore (Prof P K Shetty MDS), Department of General Medicine (A Shirali MD), Department of Physiology (P A Shirali MD), Kasturba Medical College Mangalore (Prof B Unnikrishnan MD), Forensic Medicine and Toxicology (A Vivek MD), Manipal Academy of Higher Education, Manipal, India; Manipal College of Health Professions (W Dkhar PhD, V Jaganathan PhD), Kasturba Medical College, Manipal (S Koulmane Laxminarayana MD), Department of Gastroenterology and Hepatology (Prof S Shetty MD), Manipal Academy of Higher Education, Udupi, India; Independent Consultant, Bridgewater, NJ, USA (O P Doshi MS); Department of Cardiology (R P Doshi MD), Hackettstown Medical Center, Hackettstown, NJ, USA; Newton Medical Center, Sparta, NJ, USA (R P Doshi MD); NIMS Institute of Public Health and Governance (V S Dsouza MSc), NIMS University Rajasthan, Jaipur, India; Department of Pharmacology (S Dutta MD), Department of Community and Family Medicine (K M Jasani MD), Institute of National Importance (K Joshi MD), All India Institute of Medical Sciences, Rajkot, India; College of Medicine (S Dutta PhD), Ajman University, Ajman, United Arab Emirates; Department of Pharmaceutical Sciences (B M Ejeta MSc), Dibrugarh University, Dibrugarh, India; Department of Clinical Laboratory Sciences (A Farhana PhD), Jouf University, Sakaka, Saudi Arabia; Medical Center (R Ganduboina MD), Cedar Sinnai Medical Center, Los Angeles, CA, USA; School of American Education (B Ganesan PhD), Institute of Health & Management, Australia, Melbourne, VIC, Australia; School of Engineering (B Ganesan PhD), Swinburne University of Technology, Melbourne, VIC, Australia; Department of Emergency Medicine (E Ganessane MD), Trauma and Emergency Medicine Department (G Rajendran MD), All India Institute of Medical Sciences (AIIMS), Madurai, Madurai, India; Amity Institute of Pharmacy (Prof R K Gautam PhD), Amity University Uttar Pradesh, Noida, India; Department of Medicine (V Gill MBBS), Rutgers University, New Brunswick, NJ, USA; Department of Zoology (B R Giri PhD), KKS Women's College, Balasore, India; Department of Medicine (A Goel MBBS), Department of Community and Family Medicine (A Ludhiadch PhD, S S Sahoo MD), Department of Human Anatomy (A Patra MD), Department of Psychiatry (J Singh MD), Department of Radiodiagnosis (P Singh MD), All India Institute of Medical Sciences, Bathinda, India; Department of Pharmaceutical Sciences and Drug Research (Prof R K Goel PhD), Department of Human Genetics (P Singh PhD), Punjabi University, Patiala, Patiala, India; Department of Health Systems and Policy Research (Prof M Golechha PhD), Indian Institute of Public Health, Gandhinagar, India; Department of Cardiology (A Gulati MD), Icahn School of Medicine at Mount Sinai, New York, NY, USA; Department of Pediatrics (Prof S Gulati MD, J Meena DM), Centre for Community Medicine (P Halder MD, S Kansal MD, M Mathur MD), Department of Biophysics (T Mohammad PhD), Department of Psychiatry (Prof R Sagar MD), Department of Radiation Oncology (V Sahni MDS), Department of Laboratory Medicine (A Singh PhD), Department of Physiology (V K Tiwari MD), All India Institute of Medical Sciences, New Delhi, India; Department of Nephrology (A K Gupta PharmD), Max Super Speciality Hospital, New Delhi, India; Department of Anaesthesia (Prof L Gupta MD), Community Medicine (S Puri MD), Maulana Azad Medical College, New Delhi, India; Department of Microbiology and Parasitology (A Hafiz PhD), Umm Al-Qura University, Makkah, Saudi Arabia; Epidemiology Programme (A Hafiz PhD), London School of Hygiene & Tropical Medicine, London, UK; Department of Pharmaceutical Technology (Prof M S Hasnain PhD), Adamas University, Kolkata, India; School of Engineering and Technology (O Hassan Ahmed PhD, Prof M Hosseinzadeh PhD), Institute of Research and Development (P Khoshvaght BSc, S Khoshvaght MSc), School of Engineering & Technology (P Khoshvaght BSc, S Khoshvaght MSc), Duy Tan University, Da Nang, Viet Nam; Department of AI (Prof M Hosseinzadeh PhD), Galgotias University, Greater Noida, India; Department of Clinical Pharmacy (M Imam PhD), Department of Pharmaceutical Chemistry (M Kamal PhD), Prince Sattam bin Abdulaziz University, Al Kharj, Saudi Arabia; Center for Health Research (M Imran PhD, A Khan PhD), Northern Border University, Arar, Saudi Arabia; Department of Health Research (L R Inbaraj MD), ICMR National Institute for Research in Tuberculosis, Chennai, India; Department of Health information Management (D Iqbal PhD), Buraydah Private Colleges, Al-Qassim, Saudi Arabia; International Institute of Migration and Development, Trivandrum, Trivandrum, India (C Irshad PhD); Department of Microbiology (M A Isa PhD), University of Maiduguri, Maiduguri, Nigeria; Department of Biotechnology (M A Isa PhD), Sharda University, Greater Noida, India; College of Nursing (J Jacob MSc), Department of Psychiatry (A Parmar DM), Department of Medicine (S Rath MBBS), All India Institute of Medical Sciences, Bhubaneswar, India; Department of Health Informatics (Prof Q Jamal PhD), Department of Basic Health Sciences (Prof M I Khan PhD), Qassim University, Buraydah, Saudi Arabia; Department of Biochemistry (Prof S Jayaram MD), Government Medical College, Mysuru, India; Department of Medicine (Prof F K Jebasingh DM), University of Melbourne, Melbourne, VIC, Australia; NGSM Institute of Pharmaceutical Sciences (J Jose PhD), KS Hegde Medical Academy (S S Shetty MD), Nitte University, Mangalore, India; Department of Community Medicine (J Jose MD), Jubilee Mission Medical College & Research Institute, Thrissur, Thrissur, India; Department of Community Medicine (N Joseph MD, N Kumar MD, R Motappa MD, R Thapar MD), Kasturba Medical College (G A Menezes PhD), Department of Anatomy (B Murlimanju MD), Department of Forensic Medicine and Toxicology (Prof J Padubidri MD, Prof B K Shetty MD), Kasturba Medical College, Mangalore (Prof S S Rao MD), Department of Obstetrics and Gynaecology (A Shetty MS), Manipal Academy of Higher Education, Mangalore, India; Department of Oral and Maxillofacial Pathology and Microbiology (V Kadashetti MDS), Krishna Vishwa Vidyapeeth Deemed to be University, Karad, India; Department of Endocrinology (S Kalra DM), Bharti Hospital Karnal, Karnal, India; Care and Public Health Research Institute (CAPHRI) (R Kamath MHA), Maastricht University, Maastricht, Netherlands; Department of Community Medicine (S Kannan S MD), ESIC Medical College and Hospital Chennai, Chennai, India; Analytical and Applied Economics (S K Kar MPhil), Department of Analytical and Applied Economics (B Malik MPhil, S S Mohanty PhD, Prof H Rout PhD, C Swain MPhil), RUSA Centre of Excellence in Public Policy and Governance (Prof H Rout PhD), UGC Centre of Advanced Study in Psychology (Prof M Satpathy PhD), Utkal University, Bhubaneswar, India; Amity Stem Cell Institute (ASCI) (Prof M K Kashyap PhD), Department of Mathematics (Prof K Kumar PhD), Amity University Haryana, Gurugram, India; Department of Gastroenterology (A Kaur MD), State University of New York, Syracuse, NY, USA; Department of Radiology (H Kaur MD), Columbia University Medical Center, New York, NY, USA; Department of Surgery and Cancer (S Kaur PhD), Imperial College London, London, UK; Department of Clinical Research and Epidemiology (K Kaushal MD), Institute of Liver and Biliary Sciences, New Delhi, India; Department of Biotechnology and Microbiology (M Khan PhD), Khandelwal College of Management, Science and Technology, Bareilly, India; Department of Public Health (J Khubchandani PhD), New Mexico State University, Las Cruces, NM, USA; Ashok & Rita Patel Institute of Physiotherapy (P Khuman PhD, N R Patel MPT, D S Shah MPT), Charotar University of Science and Technology, Changa, India; School of Pharmaceutical Sciences (Prof L Kishore PhD), CGC University, Mohali, Mohali, India; Department of Anthropology (Prof K Krishan PhD), Department of Forensic Science (Prof V Sharma PhD), Panjab University, Chandigarh, India; Department of Physiology (B Krishnan MD), Sree Balaji Medical College and Hospital, Chennai, India; Centre for Biotechnology (A Kuanar PhD, S K Panda PhD), Siksha 'O' Anusandhan Deemed to be University, Bhubaneswar, India; Rajendra Institute of Medical Sciences (A Kujur PharmD), Ministry of Health and Family Welfare, Ranchi, India; Amity Institute of Health Allied Sciences (Prof C Kumar PhD), Amity University Noida, Uttar Pradesh, Noida, India; Department of Community Medicine (D Kumar MD, N Kumari MSc), Department of Anaesthesiology (T Kumar DNB), Rajendra Institute of Medical Sciences, Ranchi, India; Lady Hardinge Medical College (M Kumar MD), Lady Hardinge Medical College, New Delhi, India; Department of Physical Medicine and Rehabilitation (M Kumar PhD), University of Utah Health, Salt Lake City, UT, USA; Wits Advanced Drug Delivery Platform Research Unit (Prof P Kumar PhD), Wits Infectious Diseases and Oncology Research Institute (Prof P Kumar PhD), University of the Witwatersrand, Johannesburg, South Africa; Faculty of Education (S Kumar PhD), Department of Forensic Science (Prof B K Sharma PhD, M Walia MPhil), Shree Guru Gobind Singh Tricentenary University, Gurugram, India; Department of Economics (S Kumar PhD), Central University of Haryana, Jant-Pali, India; IITM Pravartak Technologies Foundation (S Kumaran PhD), IITM Pravartak Technologies Foundation, Chennai, India; Nursing & Midwifery Research Department (NMRD) (J Kunjavara PhD), Corporate Nursing and Midwifery Research Department (K Mannethodi MPH), Research Department (K Singh PhD), Hamad Medical Corporation, Doha, Qatar; Division of Cardiology (K Kurpad MD), University of Texas, Galveston, TX, USA; Division of Evidence Synthesis (C Lahariya MD), Foundation for People-centric Health Systems, New Delhi, India; Division of Lifestyle Medicine (C Lahariya MD), Centre for Health: The Specialty Practice, New Delhi, India; Department of Chemistry (Prof A Lakhani PhD), Dayalbagh Educational Institute, Agra, India; Communicable Diseases (A Velayudhan MPH), Indian Council of Medical Research, New Delhi, India (D K Lal MD, R Mukherjee MD); Department of Otorhinolaryngology (Prof S Lasrado MS), Father Muller Medical College, Mangalore, India; Ashok & Rita Patel Institute of Physiotherapy (S D Lourembam PhD, D Modi MPT, B U Patel MPT, S P Swami MPT), Charotar University of Science and Technology, Anand, India; Institute of Health (Prof T Malik PhD), Jimma University, Jimma, Ethiopia; Division of Research and Development (Prof T Malik PhD), Research and Development Cell (V Srivastava PhD), Lovely Professional University, Phagwara, India; Internal Medicine Department (L Manjani MD), MedStar Health, Washington, DC, USA; Department of Medicine (S Manoharan MBBS), Tirunelveli Medical College, Tirunelveli, India; College of Medicine and Health Sciences (V Marakala MD), National University of Science and Technology, Oman, Sohar, Oman; Department of Community Medicine (J Mary MD), Sri Balaji Vidyapeeth (Deemed to be University), Pondicherry, India; Department of Community Medicine (M Mathur MD), Geetanjali Medical College and Hospital in Udaipur India, Udaipur, India; Turning Point, Eastern Health Clinical School (S Melwani PhD), Monash University, Melbourne, VIC, Australia; Department of Medical Microbiology and Immunology (G A Menezes PhD), Trinity Medical Sciences University, St. Vincent, Saint Vincent and the Grenadines; Department of Pathology (Prof R G Menezes MD), Imam Abdulrahman Bin Faisal University, Dammam, Saudi Arabia; Multidisciplinary Department of Medical-Surgical and Dental Specialties (G Minervini PhD), University of Campania Luigi Vanvitelli, Naples, Italy; Department of Public Health Dentistry (Prof G Mini PhD), Saveetha Institute of Medical and Technical Sciences (SIMATS), Chennai, India; Global Institute of Public Health (Prof G Mini PhD), Ananthapuri Hospitals and Research Institute, Trivandrum, India; Department of Community Medicine (K Mishra MD), ESIC Medical College and Hospital, Andheri, Mumbai, Mumbai, India; Thumbay College of Management and AI in Healthcare (V Mishra PhD), Department of Biomedical Sciences (P Sengupta PhD), Gulf Medical University, Ajman, United Arab Emirates; Research and Development Department (V Mishra PhD), Panacea Institute of Interdisciplinary Research and Education, Varanasi, India; Faculty of Medicine and Health (S Mitra PhD), University of Sydney, Sydney, NSW, Australia; School of Clinical Medicine (S Mitra PhD), University of New South Wales, Sydney, NSW, Australia; Department of Forensic Medicine and Toxicology (C Mittal MD), All India Institute of Medical Sciences, Patna, India; Department of Medicine (O Mohammed MBBS), Government Medical College Kozhikode, Kozhikode, India; School of Health Sciences (S Mohan PhD), University of Petroleum and Energy Studies, Dehradun, India; Centre for Biotechnology (S Mohapatra PhD, Prof S Nayak PhD, A Sahoo PhD, B K Sahu MSc, P Verma PhD), Siksha 'O' Anusandhan (Deemed to be University), Bhubaneswar, India (A K Verma PhD); Knowledge Management Department (S Mukherjee PhD), Prahlad Omkarwati Foundation (POF), Mumbai, India; Independent Consultant, New Delhi, India (S Mukherjee PhD, P Sood PhD); Centre for Early Childhood Caries Research (Prof S Murugan PhD), Department of Biotechnology (V Selvaraj PhD), Sri Ramachandra University, Chennai, India; Santosh University, New Delhi, India (Prof S Murugan PhD); Department of Research Methods (S Muthu PhD), Orthopaedic Research Group, Coimbatore, India; Central Research Laboratory (S Muthu PhD), Vinayaka Mission’s Research Foundation (Deemed to be University), Puducherry, India; School of Public Health Sciences and Technology (Prof R Muthusamy PhD), Malla Reddy Vishwavidyapeeth, Hyderabad, India; Vivekanandha College of Arts and Science for Women (D Nagarajan Balachandran PhD), Vivekanandha Educational Institution, Tiruchengode, India; College of Medicine and Public Health (G R Naik PhD), Flinders University, Adelaide, SA, Australia; Department of Computer Science and IT (G R Naik PhD), Torrens University, Adelaide, SA, Australia; Department of Oral and Craniomaxillofacial Surgery (P Nalabothu PhD), Department of Pediatrics Oral Health and Orthodontics (P Nalabothu PhD), University of Basel, Basel, Switzerland; Dental Research Cell (D Nandi MPH), Dr. D. Y. Patil University, Pune, India; Suraj Eye Institute, Nagpur, India (V Nangia PhD); Ashok & Rita Patel Institute of Physiotherapy (D M Paija MPT), Charotar University of Science and Technology, Nadiad, India; Community Medicine and Family Medicine (D Pal MD), Community Medicine (S Palepu MD), All India Institute of Medical Sciences, Kalyani, India; Institute of Physiotherapy (V Palaniswamy PhD), Srinivas University, Mangalore, India; School of Sciences (Prof D Pande Katare PhD), Department of Biotechnology and Microbiology (D Shrivastav PhD), Department of Biotechnology, Microbiology and Forensic Science (S Singh PhD), Noida International University, Greater Noida, India; Department of Health Systems and Implementation Research (A Pandey MPH), International Institute of Health Management Research, New Delhi, India; Institute for Population and Social Research (A Pandey MPH), Mahidol University, Nakhornpathom, Thailand; Division of Health Policy and Management (R R Parikh MD), University of Minnesota, Minneapolis, MN, USA; Forensic Medicine and Toxicology (P Parmar MD), Department of Community Medicine and Family Medicine (A M Peddireddy MD), All India Institute of Medical Sciences, Hyderabad, India; Department of Cardiology (K Patel MD), University of Kansas Medical Center, Kansas City, KS, USA; Department of Cardiology (K Patel MD), US Department of Veterans Affairs (VA), Kansas City, MO, USA; Department of Physical Therapy (M L Patel DPT), Right Motion Physical Therapy PT PC., Woodbury, NY, USA; Department of Computer Science and Bioscience (M Patel PhD), Marwadi University, Rajkot, India; Department of Cardiovascular Medicine (N N Patel MD), University of Tennessee, Nashville, TN, USA; Department of Research and Training (S K Patel PhD), Population Council Institute, New Delhi, India; Zoology Section (S Patel PhD), Regional Institute of Education (RIE), Ajmer, India; Ingenus Pharmaceuticals, LLC (S Patel MS), Long Island University, Brooklyn, Fairfield, NJ, USA; Department of Physiology (A Patil MD), College of Nursing (R V R MSc), All India Institute of Medical Sciences, Nagpur, India; Department of Haematology and Oncology (S S Patil PhD), Shenzhen Children's Hospital, Shenzhen, China; College of Dental Medicine (Prof S Patil PhD), Roseman University of Health Sciences, South Jordan, UT, USA; Department of Community Medicine (M Patni MD), RAK College of Nursing (R K Ravi PhD), Department of Pharmacology (S Satyam PhD), RAK Medical and Health Sciences University, Ras Al Khaimah, United Arab Emirates; Department of Genetics (S Pawar PhD), Yale University, New Haven, CT, USA; Department of Data Management and Analysis (R Poluru PhD), The INCLEN Trust International, New Delhi, India; Maternal and Child Health Research Centre (S Puthussery PhD), University of Bedfordshire, Luton, UK; Department of Biostatistics, Epidemiology, and Informatics (J Puvvula PhD), University of Pennsylvania, Philadelphia, PA, USA; Clinical Laboratory Sciences (S O Rab PhD), King Khalid University, Abha, Saudi Arabia; Department of Medical Oncology (Prof V Radhakrishnan MD), Cancer Institute (W.I.A), Chennai, India; Department of Epidemiology (P Raghuveer MD), National Institute of Mental Health and Neurosciences, Bengaluru, India; Department of Pharmaceutical Analysis (A Rai PhD), National Institute of Pharmaceutical Education and Research, Hajipur, Hajipur, India; Dr. Rajendra Prasad Government Medical College, Tanda, Kangra, India (Prof S K Raina MD); Department of Cardiovascular Medicine (J Rajendran MD), Cleveland Clinic, Cleveland, OH, USA; Centre for Chronic Disease Control, New Delhi, India (P Rajput PhD); Department of Anatomy (C Ramasamy MD), Govt. Siddhartha Medical College, Vijayawada, India; Department of Radiology (S Ramasamy MD), Stanford University, Stanford, CA, USA; All India Institute of Medical Sciences, Bilaspur, India (L Rana MD); Department of Community Medicine (R Rao MD), ESIC Medical College & PGIMSR & Model Hospital, Rajajinagar, Bengaluru, India; Department of Internal Medicine (K Rayapureddi MD), Armed Forces Medical College, Pune, India; Yenepoya Research Centre (B Rekadwad PhD), Yenepoya University, Mangalore, India; Faculty of Allied Health Sciences (Prof M R Rizvi PhD), Santosh (Deemed to be University), Delhi, India; Department of Community Medicine (R Rohilla MD), Government Medical College, Chandigarh, India; College of Applied and Health Sciences (A Sah PhD), A' Sharqiyah University, Ibra, Oman; National Institute for Research in Bacterial Infections (ICMR NIRBI) (Prof I Saha PhD), Indian Council of Medical Research, Kolkata, India; Biotechnology Research Center (Prof A Sahebkar PhD), Mashhad University of Medical Sciences, Mashhad, Iran; Department of Family and Generations (H Sahoo PhD), International Institute for Population Sciences, Mumbai, India; Canadian Red Cross (K Sahu PhD), Red Cross, Ottawa, ON, Canada; Department of Health Research (M C Sahu PhD), ICMR-Regional Medical Research Centre, Bhubaneswar, Bhubaneswar, India; Department of Forensic Biology (S G Sangle PhD), Government Institute of Forensic Science Chhatrapati Sambhajinagar, Chhatrapati Sambhajinagar, India; Department of Pediatrics (Prof R Sanjeev MD), SRM Medical College Hospital And Research Centre, Kattankulathur, India; Department of Pediatrics (S Sankararaman MD), University Hospitals Rainbow Babies & Children's Hospital, Cleveland, OH, USA; Department of Pediatrics (S Sankararaman MD), Case Western Reserve University, Cleveland, OH, USA; Independent Consultant, Thiruvananthapuram, India (S Y I Saraswathy PhD); CVM Nursing College (Prof S S Sarate MSc), Rita A. Patel Institute of Physiotherapy (S Shah PhD), The Charutar Vidya Mandal (CVM) University, Anand, India; Indira Gandhi Medical College and Research Institute, Puducherry, India (A Saravanan MD); Department of Epidemiology (M Sathya Narayanan MBBS), Department of Clinical Research (V Srinivasalu MBBS), National Institute for Research in Tuberculosis, Chennai, India; Udyam-Global Association for Sustainable Development, Bhubaneswar, India (Prof M Satpathy PhD); Department of Food Processing and Nutrition (J Saulam PhD), Karnataka State Akkamahadevi Women University, Vijayapura, India; Department of Preventive and Social Medicine (G Saya MD), Jawaharlal Institute of Postgraduate Medical Education and Research, Puducherry, India; Faculty of Dentistry (Prof S Selvaraj PhD), University of Puthisastra, Phnom Penh, Cambodia; Emergency Department (S Senthilkumaran PhD), Manian Medical Centre, Erode, India; Department of Pharmacy Practice (M Shabil MPharm), MS Ramaiah University of Applied Sciences, Bangalore, India; Charotar University of Science and Technology (CHARUSAT) (S A Shah MPT), Ashok and Rita Patel Institute of Physiotherapy, Changa, India; Department of Plantation Spices Medicinal and Aromatic Crops (Prof A B Sharangi PhD), Bidhan Chandra Krishi Viswavidyalaya Agricultural University in India, Mohanpur, India; School of Agriculture, Food and Ecosystem Sciences (Prof A B Sharangi PhD), University of Melbourne, Burnley, VIC, Australia; Department of Physiotherapy (A Sharma PhD), Amity University Uttar Pradesh, Noida, Noida, India; Government Medical College, Chennai, India (A Sharma MBBS); Amity Institute of Biotechnology (A Sharma PhD, E Upadhyay PhD), Amity University Rajasthan, Jaipur, India; Department of Biotechnology (B Sharma PhD), Graphic Era (Deemed to be University), Dehradun, India; Department of Biotechnology (D Sharma PhD), Noida Institute of Engineering & Technology, Greater Noida, India; Graphic Era (Deemed to be University) (K Sharma PhD), Department of Chemistry, Dehradun, India; Department of Social and Behavioral Health (Prof M Sharma PhD), University of Nevada Las Vegas, Las Vegas, NV, USA; Department of University Institute of Biotechnology (R Sharma PhD), Chandigarh University, Gharuan, India; Alva's Institute of Medical Sciences & Research Centre (Prof M Shetty MD), Rajiv Gandhi University of Health Sciences, Moodubidire, India; Department of Pharmacology (H Singh DM), Government Medical College and Hospital, Chandigarh, India; School of Pharmaceutical Sciences (H Singh PhD), IFTM University, Moradabad, India; School of Medicine (Prof J A Singh MD), Baylor College of Medicine, Houston, TX, USA; Department of Medicine Service (Prof J A Singh MD), US Department of Veterans Affairs (VA), Houston, TX, USA; Department of Community Medicine (M Singh MD), Department of Health, Delhi, India; Institute of Medical Sciences (S Singh PhD), Banaras Hindu University, Varanasi, India; Mehta Family School of Biosciences and Biomedical Engineering (S Singh MSc), Indian Institute of Technology Indore, Indore, India; Department of Community Medicine (S Singh MD), Veer Chandra Singh Garhwali Government Institute of Medical Science and Research, Srinagar Garhwal, India; Amity Institute of Public Health and Hospital Administration (A P Sinha PhD), Amity University Noida, Delhi, India; Department of Preventive Cardiology (S Soni MD), Houston Methodist Hospital, Houston, TX, USA; Department of Management, Policy and Community Health (S Soni MD), University of Texas, Houston, TX, USA; Department of Public Health and Community Medicine (Prof C T Sreeramareddy MD), International Medical University, Kuala Lumpur, Malaysia; Department of Pharmacology and Therapeutics (Prof K Sridharan DM), Arabian Gulf University, Manama, Bahrain; College of Health and Public Service (S Sriram PhD), University of North Texas, Denton, TX, USA; Department of Medical Sciences (Prof V Subramaniyan PhD), Sunway University, Subang Jaya, Malaysia; Integrative Epidemiology Unit (A Suhag PhD), University of Bristol, Bristol, UK; Department of Environmental, Agricultural and Occupational Health (J Taiba PhD), University of Nebraska Medical Center, Omaha, NE, USA; Internal Medicine (J Tewari MD), Baptist Hospitals Of Southeast Texas, Beaumont, TX, USA; Faculty of Medicine (Prof L Thangavelu PhD), University of Cyberjaya, Selangor, Malaysia; Amrita Vishwa Vidyapeetham (Prof K R Thankappan MD), Amrita Institute of Medical Sciences, Kochi, India; Department of Pharmaceutical Sciences and Natural Products (Prof S Thareja PhD), Central University of Punjab, Bathinda, Bathinda, India; Department of Obstetrics and Gynecology (M Thayumana Sundaram MS), St. John's National Academy of Health Sciences, Miami, FL, USA; Department of Applied Bioscience (Prof M Thiruvengadam PhD), Konkuk University, Seoul, South Korea; Department of Gastroenterology (N K Thomas MD), St. Luke's Hospital, Patanamthitta, India; Department of Systems Neuroscience (V K Tiwari MD), Tohoku University, Sendai, Japan; ESIC Medical College PGIMSR and Model Hospital, Bengaluru, India (S Tumkur Narayanappa BSc); ESIC Alumni Association, Bengaluru, India (S Tumkur Narayanappa BSc); Independent Consultant, St. Augustine, Trinidad and Tobago (S Umakanthan MD); North Central Regional Health Authority, Champ Fleurs, Trinidad and Tobago (S Umakanthan MD); Section of Advanced Heart Failure and Transplant (D Uppal MD), Northwell Health / North Shore University Hospital, Manhasset, NY, USA; College of Health and Sport Sciences (A G Vaithinathan MSc), University of Bahrain, Zallaq, Bahrain; Department of Community Medicine (S Vajjala MD), Government Medical College Yadadri, Yadadri, India; Department of Epidemiology and Public Health (S Vajjala MD), Sankhyatraya Science and Research Association, New Delhi, India; Department of Community Medicine (P Varma MD, S Yahoo MD), Apollo Institute of Medical Sciences and Research, Hyderabad, India; School of Epidemiology (A Velayudhan MPH), Indian Institute of Health Management Research University, Jaipur, India; Department of Pediatrics (Prof R Vidavalur MD), Cornell University, Ithaca, NY, USA; Cardiac Electrophysiology (S C Vipparthy MD), St Bernard’s Medical Center, Jonesboro, AR, USA; Department of Pharmaceutical Chemistry (Prof T A Wani PhD), Department of Biochemistry (Prof S Zargar PhD), King Saud University, Riyadh, Saudi Arabia; Department of Environmental Health and Epidemiology (V Yadav MD), National Institute for Research in Environmental Health, Bhopal, India; Department of Community Medicine (S Yogeshkumar MD), KLE Academy of Higher Education and Research, Belagavi, India

## Authors’ Contributions

### Writing the first draft of the manuscript

Rakhi Dandona, G Anil Kumar, Haley Comfort, and Theresa A McHugh.

### Providing data or critical feedback on data sources

Bhoomadevi A, Rizwan Suliankatchi Abdulkader, Anirudh Balakrishna Acharya, Swetha Acharya, Mohd Adnan, Vaishali Aggarwal, Naved Ahmad, Saheem Ahmad, Mehrunnisha Sharif Ahmed, Saif Ahmed, Mohd Akbar, Mohammad Jahoor Alam, Margret Beaula Alocious Sukumar, AH Sruthi Anil Kumar, Kabilan Annadurai, Sadaf Anwar, Gururaj Arakeri, Jesu Arockiaraj, Mohammad Saquib Ashraf, Syed Amir Ashraf, Ashish Awasthi, Giridhara Rathnaiah Babu, Raju Balaji, Bhavik Bansal, Narasimha M Beeraka, Gulam Saidunnisa Begum, Uzma Iqbal Belgaumi, Om Prakash Prakash Bera, Sonu Bhaskar, Arushee Bhatnagar, Priyadarshini Bhattacharjee, Sudip Bhattacharya, Gurjit Kaur Bhatti, Jasvinder Singh Bhatti, Bijit Biswas, Monirujjaman Biswas, Jyoti Brahmaiah, Baskaran Chandrasekaran, Vijay Kumar Chattu, Sirshendu Chaudhuri, Waseem Chauhan, Akhilanand Chaurasia, Saravanan Chinnaiyan, Shivani Chopra, Haley Comfort, Mayank Dalakoti, Lalit Dandona, Rakhi Dandona, Aso Mohammad Darwesh, Dhruva Dave, Vinoth Gnana Chellaiyan Devanbu, Devananda Devegowda, Sagnik Dey, Redolen Rose Dhar, S Siva Prasad Dora, Ojas Prakashbhai Doshi, Rajkumar Prakashbhai Doshi, Siddhartha Dutta, Aisha Farhana, Rohit Ganduboina, Balasankar Ganesan, Balasubramanian Ganesh, Ezhilkugan Ganessane, Vinod Kumar Garg, Archit Goel, Rajesh Kumar Goel, Mahaveer Golechha, Amit Gulati, Abdul Hafiz, Pritam Halder, Md Imtaiyaz Hassan, Omed Hassan Ahmed, Simon I Hay, Mehdi Hosseinzadeh, MD Talib Hussain, Jaison Jacob, Jerin James, Rajiv Janardhanan, Krishna Mahendrabhai Jasani, Shubha Jayaram, Bijay Mukesh Jeswani, Jobin Jose, Krupal Joshi, Hema Shree K, Reddy Praneeth Karnam, Faizan Zaffar Kashoo, Manoj Kumar Kashyap, Nicholas J Kassebaum, Avleen Kaur, Harleen Kaur, Mohd Adnan Kausar, Kanica Kaushal, Maseer Khan, Mohammad Idreesh Khan, Mohd Faheem Khan, Parisa Khoshvaght, Samira Khoshvaght, Jagdish Khubchandani, Sanjay Kini B, Ladli Kishore, Shivakumar KM, Sindhura Lakshmi Koulmane Laxminarayana, Kewal Krishan, Nitya Krishnasamy, Ashwini Kumar, Chandan Kumar, Dewesh Kumar, G Anil Kumar, Jogender Kumar, Kamal Kumar, Manoj Kumar, Mukesh Kumar, Pawan Kumar, Pradeep Kumar, Sandeep Kumar, Sumit Kumar, Subramanian Kumaran, Nisha Kumari, Sajeetha Kumari R, Pallavi L C, Chandrakant Lahariya, Dharmesh Kumar Lal, Savita Lasrado, Virendra S Ligade, Abdul Majid, Moutushi Majumder, Shahid Malik, Lokesh Manjani, Shaista Manzoor, Mansi Mathur, Medha Mathur, Satish Melwani, Godfred Antony Menezes, Ritesh G Menezes, Kumar Guru Mishra, Tridip Mitra, Chaitanya Mittal, Syam Mohan, Siba Sankar Mohanty, Shibani Mohapatra, Afrasim Moin, Ali H Mokdad, Rohith Motappa, Shiv Kumar Mudgal, , Reema Mukherjee, Sumoni Mukherjee, Christopher J L Murray, Dhayanithi Nagarajan Balachandran, Ganesh R Naik, Prasad Nalabothu, Sanghamitra Nayak, Mahesh P A, Jagadish Rao Padubidri, Dimpal Manilal Paija, Debkumar Pal, Vijayakumar Palaniswamy, Sujogya Kumar Panda, Deepshikha Pande Katare, Romil R Parikh, Kunal Nitinkumar Patel, Mehali Lalitbhai Patel, Mitesh Patel, Neel Navinkumar Patel, Niki Rut Patel, Sangram Kishor Patel, Shivani Patel, Sandip Shripati Patil, Shankargouda Patil, Sourav S Patnaik, Apurba Patra, Shrikant Pawar, Bala Ganesh Pichamuthu, Ramesh Poluru, Pooja Puri, Surabhi Puri, Manas Pustake, Shuby Puthussery, Jagadeesh Puvvula, Shahazad Niwazi Qurashi, Navid Rabiee, Venkatraman Radhakrishnan, Pracheth Raghuveer, Mohammad Hifz Ur Rahman, Sunil Kumar Raina, Jeffrey Pradeep Raj, Gunaseelan Rajendran, Judah Rajendran, Pushp Lata Rajpoot, Chitra Ramasamy, Shakthi Kumaran Ramasamy, Balaji Ramraj, Lokesh Rana, Chythra R Rao, Rajath Rao, Shree Rath, Ramya Kundayi Ravi, Renju Ravi, Karthik Rayapureddi, Bhagwan Narayan Rekadwad, Moattar Raza Rizvi, Syed Mohd Danish Rizvi, Hannah Elizabeth Robinson-Oden, Parameswari Royapuram Parthasarathy, Mohd Saeed, Rajesh Sagar, Ashok Kumar Sah, Vaibhav Sahni, Ambika Sahoo, Harihar Sahoo, Soumya Swaroop Sahoo, Binaya Krushna Sahu, Kirti Sundar Sahu, Mahesh Chandra Sahu, Sathish Sankar, Prasanna K Santhekadur, Maheswar Satpathy, Shakta Mani Satyam, Durairaj Sekar, Siddharthan Selvaraj, Vimalraj Selvaraj, Subramanian Senthilkumaran, Muhammed Shabil, Disha Sanjivkumar Shah, Shipraa Arpan Shah, Syed Monowar Alam Shahid, Muhammad Aaqib Shamim, Mohammed Shannawaz, Amit Baran Sharangi, Ankita Sharma, Arkansh Sharma, Avimanu Sharma, Dolly Sharma, Kamlesh Sharma, Ravi Kumar Sharma, Vishal Sharma, Adithi Shetty, B Suresh Kumar Shetty, Mahabalesh Shetty, Premalatha K Shetty, Dharmsheel Shrivastav, Samra Siddiqui, Amit Singh, Baljinder Singh, Brij Pal Singh, Harmanjit Singh, Harpreet Singh, Jasvinder A Singh, Jawahar Singh, Kalpana Singh, Paramdeep Singh, Samer Singh, Satwinder Singh, Shilpy Singh, Siddharth Singh, Surendra Singh, Vanshika Singh, Abhijeet Prasad Sinha, Mukesh Kumar Sinha, Indra Sivakumar, Farrukh Sobia, Suha Soni, Chandrashekhar T Sreeramareddy, Shyamkumar Sriram, Vipul Srivastava, Vetriselvan Subramaniyan, Meenakshi Sundari Subramaniyan Natarajan, Alisha Suhag, Thanigaivel Sundaram, Chandan Kumar Swain, Shreya P Swami, Azharuddin Sajid Syed Khaja, Jabeen Taiba, Jay Tewari, Jeevarathinam Thirumalai, Nihal Thomas, Nikhil Kenny Thomas, Vikas Kumar Tiwari, Marcos Roberto Tovani-Palone, Santhosh Kumar Tumkur Narayanappa, Srikanth Umakanthan, Bhaskaran Unnikrishnan, Era Upadhyay, Rakesh V R, Sai Mahesh Vajjala, Joe Varghese, Sampara Vasishta, Vishnu Priya Veeraraghavan, Anoop Velayudhan, Baskar Venkidasamy, Ramesh Vidavalur, Sharath Chaitanya Vipparthy, Hai Nam Vu, Megha Walia, Tanveer A A Wani, Dharmveer Yadav, Seema Zargar, and Sanjay Zodpey.

### Developing methods or computational machinery

Haley Comfort, Rakhi Dandona, G Anil Kumar, Simon I Hay, Nicholas J Kassebaum, , Ali H Mokdad, Christopher J L Murray, and Austin E Schumacher.

### Providing critical feedback on methods or results or interpretation

Bhoomadevi A, Rizwan Suliankatchi Abdulkader, Anirudh Balakrishna Acharya, Swetha Acharya, Usha Adiga, Mohd Adnan, Dhiraj Motilal Agarwal, Vaishali Aggarwal, Aqeel Ahmad, Danish Ahmad, Naved Ahmad, Saif Ahmed, Syed Anees Ahmed, Mohammad Jahoor Alam, Irfan Ali, Mohammad Daud Ali, Rafat Ali, Margret Beaula Alocious Sukumar, AH Sruthi Anil Kumar, Kabilan Annadurai, Sadaf Anwar, Razique Anwer, Gururaj Arakeri, Jesu Arockiaraj, Syed Mohammed Basheeruddin Asdaq, Mohammad Saquib Ashraf, Syed Amir Ashraf, Ashish Awasthi, Sheeba B, Prasad Hanmantrao Babar, Giridhara Rathnaiah Babu, Raju Balaji, Senthilkumar Balakrishnan, Altaf Bandy, Bhavik Bansal, Hansi Bansal, Narasimha M Beeraka, Gulam Saidunnisa Begum, Shahina Begum, Samir Bele, Uzma Iqbal Belgaumi, Om Prakash Prakash Bera, Ajeet Singh Bhadoria, Anuradha Bhardwaj, Nikha Bhardwaj, Pankaj Bhardwaj, Sonu Bhaskar, Shahnawaz Ali Bhat, Arushee Bhatnagar, Priyadarshini Bhattacharjee, Sudip Bhattacharya, Gurjit Kaur Bhatti, Jasvinder Singh Bhatti, Bijit Biswas, Monirujjaman Biswas, Trupti Bodhare, Jyoti Brahmaiah, Sravana Deepthi C, Joshua Chadwick, Rama Mohan Chandika, Baskaran Chandrasekaran, Vijay Kumar Chattu, Sirshendu Chaudhuri, Waseem Chauhan, Akhilanand Chaurasia, Snehal Prakash Chavhan, Saravanan Chinnaiyan, Shivani Chopra, Devasahayam J Christopher, Haley Comfort, Mayank Dalakoti, Lalit Dandona, P Vineeth Daniel, Roy Arokiam Daniel, Aso Mohammad Darwesh, Dhruva Dave, Vinoth Gnana Chellaiyan Devanbu, Devananda Devegowda, Sagnik Dey, Arkadeep Dhali, Kuldeep Dhama, Amol S Dhane, Redolen Rose Dhar, Winniecia Dkhar, Ojas Prakashbhai Doshi, Rajkumar Prakashbhai Doshi, Viola Savy Dsouza, Siddhartha Dutta, Sulagna Dutta, Venkata Ravikanth Eddula, Balisa Mosisa Ejeta, Devaraj Ezhilarasan, Mohammad Fareed, Aisha Farhana, Rohit Ganduboina, Balasankar Ganesan, Balasubramanian Ganesh, Ezhilkugan Ganessane, Shivaprakash Gangachannaiah, Vinod Kumar Garg, Rupesh K Gautam, Vikram Jeet Singh Gill, Bikash Ranjan Giri, Archit Goel, Rajesh Kumar Goel, Mahaveer Golechha, Sameer Vali Gopalani, Amit Gulati, Anish Kumar Gupta, Lalit Gupta, Abdul Hafiz, Pritam Halder, Mohd Anul Haq, Md Saquib Hasnain, Md Imtaiyaz Hassan, Omed Hassan Ahmed, Simon I Hay, Ramesh Holla, Mehdi Hosseinzadeh, MD Talib Hussain, Mohammad Tarique Imam, Mohd Imran, Leeberk Raja Inbaraj, Danish Iqbal, CV Irshad, Mustafa Alhaji Isa, Jaison Jacob, Vennila Jaganathan, Jerin James, Rajiv Janardhanan, Krishna Mahendrabhai Jasani, Shubha Jayaram, Felix K Jebasingh, Bijay Mukesh Jeswani, Jobin Jose, Jobinse Jose, Nitin Joseph, Krupal Joshi, Hema Shree K, Vidya Kadashetti, Mehnaz Kamal, Rajesh Kamath, Suthanthira Kannan S, Subham Kansal, Sunil Kumar Kar, Reddy Praneeth Karnam, Faizan Zaffar Kashoo, Manoj Kumar Kashyap, Nicholas J Kassebaum, Ramya Kateel, Avleen Kaur, Sandeep Kaur, Mohd Adnan Kausar, Kanica Kaushal, Abida Khan, Maseer Khan, Mohammad Idreesh Khan, Mohd Faheem Khan, Mohd Wajid Ali Khan, Sumaiya Khan, Wasif Ali Khan, Yusuf Saleem Khan, Parisa Khoshvaght, Samira Khoshvaght, Jagdish Khubchandani, P Ratan Khuman, Sanjay Kini B, Ladli Kishore, Shivakumar KM, Archana Koul, Sindhura Lakshmi Koulmane Laxminarayana, Kewal Krishan, Bindu Krishnan, Nitya Krishnasamy, Mohammed Kuddus, Anit Kujur, Ashwini Kumar, Chandan Kumar, Dewesh Kumar, Jogender Kumar, Kamal Kumar, Manoj Kumar, Mukesh Kumar, Nithin Kumar, Pawan Kumar, Pradeep Kumar, Radha Kumar, Sandeep Kumar, Sumit Kumar, Tushar Kumar, Subramanian Kumaran, Senthil Kumaran D, Nisha Kumari, Sajeetha Kumari R, Jibin Kunjavara, Krishna Prasad Kurpad, Pramod Kumar Kushawaha, Pallavi L C, Chandrakant Lahariya, Anita Lakhani, Dharmesh Kumar Lal, Savita Lasrado, Virendra S Ligade, Surbala Devi Lourembam, Abhilash Ludhiadch, Abdul Majid, Bhagabat Malik, Shahid Malik, Lokesh Manjani, Kamaruddeen Mannethodi, Sahana Manoharan, Shaista Manzoor, Vijaya Marakala, J Jenifer Florence Mary, Madhumitha Masilamani, Mansi Mathur, Medha Mathur, Jitendra Meena, Satish Melwani, Godfred Antony Menezes, Ritesh G Menezes, Giuseppe Minervini, GK Mini, Kumar Guru Mishra, Vinaytosh Mishra, Sayan Mitra, Tridip Mitra, Chaitanya Mittal, Dhruti Modi, Taj Mohammad, Omer Mohammed, Itismita Mohanty, Siba Sankar Mohanty, Shibani Mohapatra, Afrasim Moin, Ali H Mokdad, Himel Mondal, Rohith Motappa, Shiv Kumar Mudgal, Sumoni Mukherjee, Christopher J L Murray, Satta Muthu Murugan, Sathish Muthu, Raman Muthusamy, Karikalan Nagarajan, Dhayanithi Nagarajan Balachandran, Ganesh R Naik, Gurudatta Naik, Prasad Nalabothu, Girish Nandakumar, Dhruva Nandi, Vinay Nangia, Sanghamitra Nayak, Mahesh P A, Jagadish Rao Padubidri, Dimpal Manilal Paija, Debkumar Pal, Vijayakumar Palaniswamy, Sarika Palepu, Sujogya Kumar Panda, Deepshikha Pande Katare, Anuj Kumar Pandey, Romil R Parikh, Arpit Parmar, Pragnesh Parmar, Bhargav U Umeshchandra Patel, Kunal Nitinkumar Patel, Mehali Lalitbhai Patel, Mitesh Patel, Neel Navinkumar Patel, Niki Rut Patel, Sangram Kishor Patel, Satyananda Patel, Shivani Patel, Ashlesh Patil, Sandip Shripati Patil, Shankargouda Patil, Sourav S Patnaik, Mohamed Anas Patni, Apurba Patra, Shrikant Pawar, Arun Mitra Peddireddy, Bala Ganesh Pichamuthu, Ramesh Poluru, Akila Prashant, Pooja Puri, Surabhi Puri, Manas Pustake, Shuby Puthussery, Jagadeesh Puvvula, Shahazad Niwazi Qurashi, Safia Obaidur Rab, Navid Rabiee, Venkatraman Radhakrishnan, Yashpal Singh Raghav, Pracheth Raghuveer, Mohammad Hifz Ur Rahman, Amita Rai, Sunil Kumar Raina, Jeffrey Pradeep Raj, Gunaseelan Rajendran, Judah Rajendran, Pushp Lata Rajpoot, Prashant Rajput, Chitra Ramasamy, Shakthi Kumaran Ramasamy, Balaji Ramraj, Lokesh Rana, Chythra R Rao, Priyanka Rao, Rajath Rao, Suchetha S Rao, Shree Rath, Isha Rathi, Lokesh Ravi, Ramya Kundayi Ravi, Renju Ravi, Karthik Rayapureddi, Bhagwan Narayan Rekadwad, Moattar Raza Rizvi, Syed Mohd Danish Rizvi, Ravi Rohilla, Himanshu Sekhar Rout, Parameswari Royapuram Parthasarathy, Manjula S, Mohd Saeed, Rajesh Sagar, Ashok Kumar Sah, Indranil Saha, Vaibhav Sahni, Ambika Sahoo, Harihar Sahoo, Soumya Swaroop Sahoo, Binaya Krushna Sahu, Kirti Sundar Sahu, Mahesh Chandra Sahu, Sandeep G Sangle, Rama Krishna Sanjeev, Sathish Sankar, Prasanna K Santhekadur, Sivan Yegnanarayana Iyer Saraswathy, Siddaram Shivaji Sarate, Sachin C Sarode, Mukesh Kumar Sathya Narayanan, Maheswar Satpathy, Shakta Mani Satyam, Jennifer Saulam, Ganesh Kumar Saya, Durairaj Sekar, Siddharthan Selvaraj, Vimalraj Selvaraj, Pallav Sengupta, Subramanian Senthilkumaran, Muhammed Shabil, Disha Sanjivkumar Shah, Shipraa Arpan Shah, Sweni Shah, Uzma Shahab, Syed Monowar Alam Shahid, Muhammad Aaqib Shamim, Mohd Shanawaz, Devika Shanmugasundaram, Mohammed Shannawaz, Amit Baran Sharangi, Ankita Sharma, Arkansh Sharma, Avimanu Sharma, Bhoopesh Kumar Sharma, Bunty Sharma, Dolly Sharma, Kamlesh Sharma, Ravi Kumar Sharma, Vishal Sharma, Subuhi Sherwani, Mahabalesh Shetty, Shiran Shetty, Arun Shirali, Priyanka Arun Shirali, Dharmsheel Shrivastav, Samra Siddiqui, Amit Singh, Baljinder Singh, Brij Pal Singh, Harmanjit Singh, Harpreet Singh, Jasvinder A Singh, Jawahar Singh, Kalpana Singh, Paramdeep Singh, Puneetpal Singh, Samer Singh, Satwinder Singh, Shilpy Singh, Siddharth Singh, Surendra Singh, Vanshika Singh, Yadvinder Singh, Abhijeet Prasad Sinha, Mukesh Kumar Sinha, Indra Sivakumar, Farrukh Sobia, Suha Soni, Prashant Sood, Chandrashekhar T Sreeramareddy, Kannan Sridharan, Vignes Anand Srinivasalu, Shyamkumar Sriram, Vipul Srivastava, Vetriselvan Subramaniyan, Meenakshi Sundari Subramaniyan Natarajan, Alisha Suhag, Thanigaivel Sundaram, Chandan Kumar Swain, Shreya P Swami, Azharuddin Sajid Syed Khaja, Jabeen Taiba, Jay Tewari, Lakshmi Thangavelu, Kavumpurathu Raman Thankappan, Rekha Thapar, Mahalakshmi Thayumana Sundaram, Jeevarathinam Thirumalai, Muthu Thiruvengadam, Rekha Thiruvengadam, Nihal Thomas, Nikhil Kenny Thomas, Vikas Kumar Tiwari, Marcos Roberto Tovani-Palone, Santhosh Kumar Tumkur Narayanappa, Srikanth Umakanthan, Bhaskaran Unnikrishnan, Era Upadhyay, Dipan Uppal, Rakesh V R, Sai Mahesh Vajjala, Joe Varghese, Pavani Varma, Sampara Vasishta, Vishnu Priya Veeraraghavan, Anoop Velayudhan, U Venkatesh, Baskar Venkidasamy, Ramesh Vidavalur, Sharath Chaitanya Vipparthy, Athul Vivek, Megha Walia, Tanveer A A Wani, Dharmveer Yadav, Suresh Yadav, Vikas Yadav, Saba Yahoo, S Yogeshkumar, Ghazala Yunus, Seema Zargar, and Sanjay Zodpey.

### Drafting the work or revising it critically for important intellectual content

Bhoomadevi A, Anirudh Balakrishna Acharya, Gaurav Acharya, Swetha Acharya, Usha Adiga, Mohd Adnan, Dhiraj Motilal Agarwal, Danish Ahmad, Naved Ahmad, Mehrunnisha Sharif Ahmed, Syed Anees Ahmed, Mohammad Jahoor Alam, Margret Beaula Alocious Sukumar, Prapti Anjana, Kabilan Annadurai, Sadaf Anwar, Razique Anwer, Jesu Arockiaraj, Syed Mohammed Basheeruddin Asdaq, Mohammad Saquib Ashraf, Ashish Awasthi, Giridhara Rathnaiah Babu, Senthilkumar Balakrishnan, Bhavik Bansal, Hansi Bansal, Narasimha M Beeraka, Uzma Iqbal Belgaumi, Om Prakash Prakash Bera, Ajeet Singh Bhadoria, Anuradha Bhardwaj, Sonu Bhaskar, Shahnawaz Ali Bhat, Arushee Bhatnagar, Priyadarshini Bhattacharjee, Sudip Bhattacharya, Gurjit Kaur Bhatti, Jasvinder Singh Bhatti, Bijit Biswas, Monirujjaman Biswas, Sanjay C J, Joshua Chadwick, Rama Mohan Chandika, Baskaran Chandrasekaran, Vijay Kumar Chattu, Anis Ahmad Chaudhary, Sirshendu Chaudhuri, Waseem Chauhan, Akhilanand Chaurasia, Shivani Chopra, Devasahayam J Christopher, Haley Comfort, Mayank Dalakoti, Rakhi Dandona, Roy Arokiam Daniel, Dhruva Dave, Vinoth Gnana Chellaiyan Devanbu, Devananda Devegowda, Arkadeep Dhali, Amol S Dhane, Winniecia Dkhar, Ojas Prakashbhai Doshi, Rajkumar Prakashbhai Doshi, Venkata Ravikanth Eddula, Devaraj Ezhilarasan, Mohammad Fareed, Aisha Farhana, Balasankar Ganesan, Balasubramanian Ganesh, Ezhilkugan Ganessane, Shivaprakash Gangachannaiah, Vinod Kumar Garg, Rupesh K Gautam, Archit Goel, Rajesh Kumar Goel, Sameer Vali Gopalani, Sheffali Gulati, Lalit Gupta, Abdul Hafiz, Mohd Anul Haq, Shamimul Hasan, Md Saquib Hasnain, Md Imtaiyaz Hassan, Simon I Hay, Ramesh Holla, Mohd Imran, CV Irshad, Mustafa Alhaji Isa, Qazi Mohammad Sajid Jamal, Jerin James, Rajiv Janardhanan, Shubha Jayaram, Felix K Jebasingh, Bijay Mukesh Jeswani, Jobin Jose, Nitin Joseph, Hema Shree K, Vidya Kadashetti, Sanjay Kalra, Rajesh Kamath, Reddy Praneeth Karnam, Faizan Zaffar Kashoo, Manoj Kumar Kashyap, Nicholas J Kassebaum, Avleen Kaur, Mohd Adnan Kausar, Kanica Kaushal, Abida Khan, Maseer Khan, Mohd Faheem Khan, Mohd Wajid Ali Khan, Sumaiya Khan, Wasif Ali Khan, Yusuf Saleem Khan, Jagdish Khubchandani, Sanjay Kini B, Ladli Kishore, Shivakumar KM, Sindhura Lakshmi Koulmane Laxminarayana, Kewal Krishan, Bindu Krishnan, Ananya Kuanar, Mohammed Kuddus, Ashwini Kumar, Chandan Kumar, Dewesh Kumar, G Anil Kumar, Jogender Kumar, Pawan Kumar, Pradeep Kumar, Radha Kumar, Rakesh Kumar, Sandeep Kumar, Sumit Kumar, Tushar Kumar, Senthil Kumaran D, Nisha Kumari, Sajeetha Kumari R, Krishna Prasad Kurpad, Pallavi L C, Chandrakant Lahariya, Anita Lakhani, Savita Lasrado, Abhilash Ludhiadch, Abdul Majid, Shahid Malik, Tabarak Malik, Lokesh Manjani, Kamaruddeen Mannethodi, Sahana Manoharan, Shaista Manzoor, Vijaya Marakala, Medha Mathur, Theresa A McHugh, Godfred Antony Menezes, Ritesh G Menezes, Kumar Guru Mishra, Tridip Mitra, Chaitanya Mittal, Taj Mohammad, Omer Mohammed, Shibani Mohapatra, Ali H Mokdad, Himel Mondal, Rohith Motappa, Shiv Kumar Mudgal, BV Murlimanju, Christopher J L Murray, Satta Muthu Murugan, Sathish Muthu, Raman Muthusamy, Karikalan Nagarajan, Dhayanithi Nagarajan Balachandran, Prasad Nalabothu, Girish Nandakumar, Dhruva Nandi, Mahesh P A, Jagadish Rao Padubidri, Debkumar Pal, Vijayakumar Palaniswamy, Sujogya Kumar Panda, Deepshikha Pande Katare, Romil R Parikh, Kunal Nitinkumar Patel, Mehali Lalitbhai Patel, Mitesh Patel, Neel Navinkumar Patel, Satyananda Patel, Shivani Patel, Ashlesh Patil, Shankargouda Patil, Mohamed Anas Patni, Apurba Patra, Shrikant Pawar, Bala Ganesh Pichamuthu, Akila Prashant, Pooja Puri, Manas Pustake, Shuby Puthussery, Jagadeesh Puvvula, Shahazad Niwazi Qurashi, Navid Rabiee, Venkatraman Radhakrishnan, Pracheth Raghuveer, Mohammad Hifz Ur Rahman, Amita Rai, Sunil Kumar Raina, Jeffrey Pradeep Raj, Gunaseelan Rajendran, Judah Rajendran, Pushp Lata Rajpoot, Prashant Rajput, Chitra Ramasamy, Shakthi Kumaran Ramasamy, Balaji Ramraj, Lokesh Rana, Chythra R Rao, Rajath Rao, Shree Rath, Lokesh Ravi, Ramya Kundayi Ravi, Renju Ravi, Karthik Rayapureddi, Bhagwan Narayan Rekadwad, Moattar Raza Rizvi, Ravi Rohilla, Manjula S, Chandan S N, Rajesh Sagar, Ashok Kumar Sah, Indranil Saha, Amirhossein Sahebkar, Vaibhav Sahni, Ambika Sahoo, Soumya Swaroop Sahoo, Binaya Krushna Sahu, Kirti Sundar Sahu, Sandeep G Sangle, Rama Krishna Sanjeev, Sathish Sankar, Senthilkumar Sankararaman, Prasanna K Santhekadur, Aswini Saravanan, Sachin C Sarode, Mukesh Kumar Sathya Narayanan, Maheswar Satpathy, Shakta Mani Satyam, Ganesh Kumar Saya, Austin E Schumacher, Siddharthan Selvaraj, Vimalraj Selvaraj, Subramanian Senthilkumaran, Muhammed Shabil, Disha Sanjivkumar Shah, Sweni Shah, Uzma Shahab, Syed Monowar Alam Shahid, Muhammad Aaqib Shamim, Mohd Shanawaz, Devika Shanmugasundaram, Mohammed Shannawaz, Amit Baran Sharangi, Ankita Sharma, Arkansh Sharma, Avimanu Sharma, Bunty Sharma, Kamlesh Sharma, Manoj Sharma, Ujjawal Sharma, Vishal Sharma, Rekha Raghuveer Shenoy, Subuhi Sherwani, Mahabalesh Shetty, Premalatha K Shetty, Shiran Shetty, Suraj S Shetty, Arun Shirali, Priyanka Arun Shirali, Dharmsheel Shrivastav, Akanksha Singh, Brij Pal Singh, Harmanjit Singh, Jasvinder A Singh, Jawahar Singh, Kalpana Singh, Mitasha Singh, Paramdeep Singh, Poornima Suryanath Singh, Samer Singh, Satwinder Singh, Shilpy Singh, Siddharth Singh, Surendra Singh, Vanshika Singh, Yadvinder Singh, Indra Sivakumar, Farrukh Sobia, Prashant Sood, Chandrashekhar T Sreeramareddy, Kannan Sridharan, Vignes Anand Srinivasalu, Shyamkumar Sriram, Vetriselvan Subramaniyan, Meenakshi Sundari Subramaniyan Natarajan, Thanigaivel Sundaram, Azharuddin Sajid Syed Khaja, Suresh Thareja, Mahalakshmi Thayumana Sundaram, Jeevarathinam Thirumalai, Nihal Thomas, Nikhil Kenny Thomas, Vikas Kumar Tiwari, Marcos Roberto Tovani-Palone, Shadab Uddin, Srikanth Umakanthan, Bhaskaran Unnikrishnan, Era Upadhyay, Dipan Uppal, Asokan Govindaraj Vaithinathan, Sai Mahesh Vajjala, Joe Varghese, Pavani Varma, Vishnu Priya Veeraraghavan, Anoop Velayudhan, Akshaya Kumar Verma, Poonam Verma, Ramesh Vidavalur, Sharath Chaitanya Vipparthy, Tanveer A A Wani, Vikas Yadav, Saba Yahoo, S Yogeshkumar, Ghazala Yunus, and Seema Zargar.

### Managing the publications process

Rakhi Dandona, Simon I Hay, G Anil Kumar, Nicholas J Kassebaum, Ali H Mokdad, and Christopher J L Murray.
