# Supplementary material for: Stillbirths at 22- and 28-weeks’ gestation or longer for the states in India: findings from the Global Burden of Disease Study 2023
Source: Lancet Reg Health Southeast Asia. 2026 Jul 27;52:100819. doi: 10.1016/j.lansea.2026.100819 (PMC13446360; doi:10.1016/j.lansea.2026.100819)
Supplement: Supplementary file 3 [file mmc3.docx]

## India State-Level Disease Burden Initiative Stillbirths Collaborators

| **Given Names** | **Last Name** |
| --- | --- |
| Rakhi | Dandona ^A, B, C^ |
| G Anil | Kumar ^A^ |
| Haley | Comfort ^B^ |
| Theresa A | McHugh ^B^ |
| Bhoomadevi | A ^D^ |
| Rizwan Suliankatchi | Abdulkader ^E^ |
| Anirudh Balakrishna | Acharya ^F^ |
| Gaurav | Acharya ^G^ |
| Swetha | Acharya ^H^ |
| Usha | Adiga ^I^ |
| Mohd | Adnan ^J^ |
| Dhiraj Motilal | Agarwal ^K^ |
| Vaishali | Aggarwal ^L^ |
| Aqeel | Ahmad ^M^ |
| Danish | Ahmad ^N, O^ |
| Naved | Ahmad ^P^ |
| Saheem | Ahmad ^Q^ |
| Mehrunnisha Sharif | Ahmed ^R^ |
| Saif | Ahmed ^S^ |
| Syed Anees | Ahmed ^T^ |
| Mohd | Akbar ^A^ |
| Mohammad Jahoor | Alam ^J^ |
| Irfan | Ali ^U^ |
| Mohammad Daud | Ali ^V^ |
| Rafat | Ali ^W^ |
| Margret Beaula | Alocious Sukumar ^X^ |
| AH Sruthi | Anil Kumar ^Y^ |
| Prapti | Anjana ^Z^ |
| Kabilan | Annadurai ^AA^ |
| Sadaf | Anwar ^AB^ |
| Razique | Anwer ^S^ |
| Gururaj | Arakeri ^AC^ |
| Jesu | Arockiaraj ^AD^ |
| Syed Mohammed Basheeruddin | Asdaq ^AE^ |
| Mohammad Saquib | Ashraf ^AF^ |
| Syed Amir | Ashraf ^AG^ |
| Ashish | Awasthi ^AH, AI^ |
| Sheeba | B ^AJ^ |
| Prasad Hanmantrao | Babar ^AK^ |
| Giridhara Rathnaiah | Babu ^AL^ |
| Raju | Balaji ^AM^ |
| Senthilkumar | Balakrishnan ^AN^ |
| Altaf | Bandy ^AO^ |
| Bhavik | Bansal ^AP^ |
| Hansi | Bansal ^AQ^ |
| Narasimha M | Beeraka ^AR^ |
| Gulam Saidunnisa | Begum ^AS^ |
| Shahina | Begum ^AT^ |
| Samir | Bele ^AU^ |
| Uzma Iqbal | Belgaumi ^AV^ |
| Om Prakash Prakash | Bera ^AW, AX^ |
| Ajeet Singh | Bhadoria ^AY, AZ^ |
| Anuradha | Bhardwaj ^BA^ |
| Nikha | Bhardwaj ^BB^ |
| Pankaj | Bhardwaj ^BC^ |
| Sonu | Bhaskar ^BD, BE^ |
| Shahnawaz Ali | Bhat ^BF^ |
| Arushee | Bhatnagar ^BG^ |
| Priyadarshini | Bhattacharjee ^BH^ |
| Sudip | Bhattacharya ^BI^ |
| Gurjit Kaur | Bhatti ^BJ^ |
| Jasvinder Singh | Bhatti ^BK^ |
| Bijit | Biswas ^BI^ |
| Monirujjaman | Biswas ^BL^ |
| Trupti | Bodhare ^BM^ |
| Jyoti | Brahmaiah ^BN^ |
| Sravana Deepthi | C ^BO^ |
| Sanjay | C J ^BP^ |
| Joshua | Chadwick ^BQ^ |
| Rama Mohan | Chandika ^BR^ |
| Baskaran | Chandrasekaran ^BS^ |
| Vijay Kumar | Chattu ^BT, BU^ |
| Anis Ahmad | Chaudhary ^BV^ |
| Sirshendu | Chaudhuri ^BW^ |
| Waseem | Chauhan ^BX^ |
| Akhilanand | Chaurasia ^BY^ |
| Snehal Prakash | Chavhan ^BZ^ |
| Saravanan | Chinnaiyan ^CA^ |
| Shivani | Chopra ^CB^ |
| Devasahayam J | Christopher ^CC^ |
| Mayank | Dalakoti ^CD, CE^ |
| P Vineeth | Daniel ^CF^ |
| Roy Arokiam | Daniel ^CG^ |
| Aso Mohammad | Darwesh ^CH, CI^ |
| Dhruva | Dave ^CJ^ |
| Vinoth Gnana Chellaiyan | Devanbu ^CK^ |
| Devananda | Devegowda ^CL^ |
| Sagnik | Dey ^CM, CN^ |
| Arkadeep | Dhali ^CO^ |
| Kuldeep | Dhama ^CP^ |
| Amol S | Dhane ^CQ^ |
| Redolen Rose | Dhar ^CR^ |
| Winniecia | Dkhar ^CS, CT^ |
| S Siva Prasad | Dora ^A^ |
| Ojas Prakashbhai | Doshi ^CU^ |
| Rajkumar Prakashbhai | Doshi ^CV, CW^ |
| Viola Savy | Dsouza ^CX^ |
| Siddhartha | Dutta ^CY^ |
| Sulagna | Dutta ^CZ^ |
| Venkata Ravikanth | Eddula ^DA^ |
| Balisa Mosisa | Ejeta ^DB^ |
| Devaraj | Ezhilarasan ^DC^ |
| Mohammad | Fareed ^DD^ |
| Aisha | Farhana ^DE^ |
| Rohit | Ganduboina ^DF^ |
| Balasankar | Ganesan ^DG, DH^ |
| Balasubramanian | Ganesh ^DI^ |
| Ezhilkugan | Ganessane ^DJ^ |
| Shivaprakash | Gangachannaiah ^DK^ |
| Vinod Kumar | Garg ^DL^ |
| Rupesh K | Gautam ^DM^ |
| Vikram Jeet Singh | Gill ^DN^ |
| Bikash Ranjan | Giri ^DO^ |
| Archit | Goel ^DP^ |
| Rajesh Kumar | Goel ^DQ^ |
| Mahaveer | Golechha ^DR^ |
| Sameer Vali | Gopalani ^DS^ |
| Amit | Gulati ^DT^ |
| Sheffali | Gulati ^DU^ |
| Anish Kumar | Gupta ^DV, DW^ |
| Lalit | Gupta ^DX^ |
| Abdul | Hafiz ^DY, DZ^ |
| Pritam | Halder ^EA, EB^ |
| Mohd Anul | Haq ^EC^ |
| Shamimul | Hasan ^ED^ |
| Md Saquib | Hasnain ^EE^ |
| Md Imtaiyaz | Hassan ^EF^ |
| Omed | Hassan Ahmed ^CH, EG^ |
| Simon I | Hay ^B, C^ |
| Ramesh | Holla ^EH^ |
| Mehdi | Hosseinzadeh ^EG, EI^ |
| MD Talib | Hussain ^EJ^ |
| Mohammad Tarique | Imam ^EK^ |
| Mohd | Imran ^EL^ |
| Leeberk Raja | Inbaraj ^EM^ |
| Danish | Iqbal ^EN, EO^ |
| CV | Irshad ^EP^ |
| Mustafa Alhaji | Isa ^EQ, ER^ |
| Jaison | Jacob ^ES^ |
| Vennila | Jaganathan ^CS^ |
| Qazi Mohammad Sajid | Jamal ^ET^ |
| Jerin | James ^EU^ |
| Rajiv | Janardhanan ^CF^ |
| Krishna Mahendrabhai | Jasani ^EV^ |
| Shubha | Jayaram ^EW^ |
| Felix K | Jebasingh ^EX, EY^ |
| Bijay Mukesh | Jeswani ^EZ^ |
| Jobin | Jose ^FA^ |
| Jobinse | Jose ^FB^ |
| Nitin | Joseph ^FC^ |
| Krupal | Joshi ^FD^ |
| Hema Shree | K ^FE^ |
| Vidya | Kadashetti ^FF^ |
| Sanjay | Kalra ^FG, FH^ |
| Mehnaz | Kamal ^FI^ |
| Rajesh | Kamath ^FJ, FK^ |
| Suthanthira | Kannan S ^FL^ |
| Subham | Kansal ^EB^ |
| Sunil Kumar | Kar ^FM^ |
| Reddy Praneeth | Karnam ^FN^ |
| Faizan Zaffar | Kashoo ^FO^ |
| Manoj Kumar | Kashyap ^FP^ |
| Ramya | Kateel ^DK^ |
| Avleen | Kaur ^FQ^ |
| Harleen | Kaur ^FR^ |
| Sandeep | Kaur ^FS, FT^ |
| Mohd Adnan | Kausar ^AB^ |
| Kanica | Kaushal ^FU^ |
| Abida | Khan ^EL^ |
| Maseer | Khan ^FV^ |
| Mohammad Idreesh | Khan ^FW^ |
| Mohd Faheem | Khan ^FX^ |
| Mohd Wajid Ali | Khan ^FY, FZ^ |
| Sumaiya | Khan ^EF^ |
| Wasif Ali | Khan ^GA^ |
| Yusuf Saleem | Khan ^GB^ |
| Parisa | Khoshvaght ^GC, GD^ |
| Samira | Khoshvaght ^GC, GD^ |
| Jagdish | Khubchandani ^GE^ |
| P Ratan | Khuman ^GF^ |
| Sanjay | Kini B ^GG^ |
| Ladli | Kishore ^GH^ |
| Shivakumar | KM ^GI^ |
| Archana | Koul ^D^ |
| Sindhura Lakshmi | Koulmane Laxminarayana ^GJ^ |
| Kewal | Krishan ^GK^ |
| Bindu | Krishnan ^GL^ |
| Nitya | Krishnasamy ^GM^ |
| Ananya | Kuanar ^GN^ |
| Mohammed | Kuddus ^AB^ |
| Anit | Kujur ^GO^ |
| Ashwini | Kumar ^GP^ |
| Chandan | Kumar ^GQ^ |
| Dewesh | Kumar ^GR^ |
| Jogender | Kumar ^GS^ |
| Kamal | Kumar ^GT^ |
| Manoj | Kumar ^GU^ |
| Mukesh | Kumar ^GV^ |
| Nithin | Kumar ^FC^ |
| Pawan | Kumar ^CT, EA^ |
| Pradeep | Kumar ^GW, GX^ |
| Radha | Kumar ^GY^ |
| Rakesh | Kumar ^GZ^ |
| Sandeep | Kumar ^HA^ |
| Sumit | Kumar ^HB^ |
| Tushar | Kumar ^HC^ |
| Subramanian | Kumaran ^HD^ |
| Senthil | Kumaran D ^HE^ |
| Nisha | Kumari ^GR^ |
| Sajeetha | Kumari R ^HF^ |
| Jibin | Kunjavara ^HG^ |
| Krishna Prasad | Kurpad ^HH^ |
| Pramod Kumar | Kushawaha ^HI^ |
| Pallavi | L C ^GP^ |
| Chandrakant | Lahariya ^HJ, HK^ |
| Anita | Lakhani ^HL^ |
| Dharmesh Kumar | Lal ^HM^ |
| Savita | Lasrado ^HN^ |
| Virendra S | Ligade ^HO^ |
| Surbala Devi | Lourembam ^HP^ |
| Abhilash | Ludhiadch ^HQ^ |
| Abdul | Majid ^BX^ |
| Moutushi | Majumder ^A^ |
| Bhagabat | Malik ^HR^ |
| Shahid | Malik ^HS^ |
| Tabarak | Malik ^HT, HU^ |
| Lokesh | Manjani ^HV^ |
| Kamaruddeen | Mannethodi ^HW^ |
| Sahana | Manoharan ^HX^ |
| Shaista | Manzoor ^HY^ |
| Vijaya | Marakala ^HZ^ |
| J Jenifer Florence | Mary ^IA^ |
| Madhumitha | Masilamani ^IB^ |
| Mansi | Mathur ^EB^ |
| Medha | Mathur ^IC^ |
| Jitendra | Meena ^DU^ |
| Satish | Melwani ^ID^ |
| Godfred Antony | Menezes ^IE, IF^ |
| Ritesh G | Menezes ^IG^ |
| Giuseppe | Minervini ^IH, II^ |
| GK | Mini ^IJ, IK^ |
| Kumar Guru | Mishra ^IL^ |
| Vinaytosh | Mishra ^IM, IN^ |
| Sayan | Mitra ^IO, IP^ |
| Tridip | Mitra ^Y^ |
| Chaitanya | Mittal ^IQ^ |
| Dhruti | Modi ^HP^ |
| Taj | Mohammad ^IR, IS^ |
| Omer | Mohammed ^IT^ |
| Syam | Mohan ^IU, IV^ |
| Itismita | Mohanty ^O^ |
| Siba Sankar | Mohanty ^HR^ |
| Shibani | Mohapatra ^IW^ |
| Afrasim | Moin ^IX^ |
| Ali H | Mokdad ^B, C^ |
| Himel | Mondal ^IY^ |
| Rohith | Motappa ^FC^ |
| Shiv Kumar | Mudgal ^IZ^ |
| Reema | Mukherjee ^HM^ |
| Sumoni | Mukherjee ^JA, JB^ |
| BV | Murlimanju ^JC^ |
| Satta Muthu | Murugan ^JD, JE^ |
| Sathish | Muthu ^JF, JG^ |
| Raman | Muthusamy ^JH^ |
| Karikalan | Nagarajan ^JI, JJ^ |
| Dhayanithi | Nagarajan Balachandran ^JK^ |
| Ganesh R | Naik ^JL, JM^ |
| Gurudatta | Naik ^JN^ |
| Prasad | Nalabothu ^JO, JP^ |
| Girish | Nandakumar ^HE^ |
| Dhruva | Nandi ^JQ^ |
| Vinay | Nangia ^JR^ |
| Sanghamitra | Nayak ^IW^ |
| Mahesh | P A ^JS^ |
| Jagadish Rao | Padubidri ^JT^ |
| Dimpal Manilal | Paija ^JU^ |
| Debkumar | Pal ^JV^ |
| Vijayakumar | Palaniswamy ^JW^ |
| Sarika | Palepu ^JX^ |
| Sujogya Kumar | Panda ^GN^ |
| Deepshikha | Pande Katare ^JY^ |
| Anuj Kumar | Pandey ^JZ, KA^ |
| Romil R | Parikh ^KB^ |
| Arpit | Parmar ^KC^ |
| Pragnesh | Parmar ^KD^ |
| Bhargav U Umeshchandra | Patel ^HP^ |
| Kunal Nitinkumar | Patel ^KE, KF^ |
| Mehali Lalitbhai | Patel ^KG^ |
| Mitesh | Patel ^KH^ |
| Neel Navinkumar | Patel ^KI^ |
| Niki Rut | Patel ^GF^ |
| Sangram Kishor | Patel ^KJ^ |
| Satyananda | Patel ^KK^ |
| Shivani | Patel ^KL^ |
| Ashlesh | Patil ^KM^ |
| Sandip Shripati | Patil ^KN^ |
| Shankargouda | Patil ^KO^ |
| Sourav S | Patnaik ^KP^ |
| Mohamed Anas | Patni ^KQ^ |
| Apurba | Patra ^KR^ |
| Shrikant | Pawar ^KS^ |
| Arun Mitra | Peddireddy ^KT^ |
| Bala Ganesh | Pichamuthu ^KU^ |
| Ramesh | Poluru ^KV^ |
| Akila | Prashant ^KW^ |
| Pooja | Puri ^KX^ |
| Surabhi | Puri ^KY^ |
| Manas | Pustake ^KZ^ |
| Shuby | Puthussery ^LA^ |
| Jagadeesh | Puvvula ^LB^ |
| Shahazad Niwazi | Qurashi ^LC^ |
| Safia Obaidur | Rab ^LD^ |
| Navid | Rabiee ^LE^ |
| Venkatraman | Radhakrishnan ^LF^ |
| Yashpal Singh | Raghav ^LG^ |
| Pracheth | Raghuveer ^LH^ |
| Mohammad Hifz Ur | Rahman ^LI^ |
| Amita | Rai ^LJ^ |
| Sunil Kumar | Raina ^LK^ |
| Jeffrey Pradeep | Raj ^LL^ |
| Gunaseelan | Rajendran ^LM^ |
| Judah | Rajendran ^LN^ |
| Pushp Lata | Rajpoot ^LO^ |
| Prashant | Rajput ^LP^ |
| Chitra | Ramasamy ^LQ^ |
| Shakthi Kumaran | Ramasamy ^LR^ |
| Balaji | Ramraj ^LS^ |
| Lokesh | Rana ^LT^ |
| Chythra R | Rao ^GG^ |
| Priyanka | Rao ^LU^ |
| Rajath | Rao ^LV^ |
| Suchetha S | Rao ^LW^ |
| Shree | Rath ^LX^ |
| Isha | Rathi ^LY^ |
| Lokesh | Ravi ^LZ^ |
| Ramya Kundayi | Ravi ^MA^ |
| Renju | Ravi ^MB^ |
| Karthik | Rayapureddi ^MC^ |
| Bhagwan Narayan | Rekadwad ^MD^ |
| Moattar Raza | Rizvi ^ME^ |
| Syed Mohd Danish | Rizvi ^MF^ |
| Hannah Elizabeth | Robinson-Oden ^B^ |
| Ravi | Rohilla ^MG^ |
| Himanshu Sekhar | Rout ^HR, MH^ |
| Parameswari | Royapuram Parthasarathy ^MI^ |
| Manjula | S ^MJ^ |
| Chandan | S N ^MJ^ |
| Mohd | Saeed ^J^ |
| Rajesh | Sagar ^MK^ |
| Ashok Kumar | Sah ^ML^ |
| Indranil | Saha ^MM, MN^ |
| Amirhossein | Sahebkar ^MO, MP^ |
| Vaibhav | Sahni ^MQ^ |
| Ambika | Sahoo ^IW^ |
| Harihar | Sahoo ^MR^ |
| Soumya Swaroop | Sahoo ^HQ^ |
| Binaya Krushna | Sahu ^IW^ |
| Kirti Sundar | Sahu ^MS^ |
| Mahesh Chandra | Sahu ^MT^ |
| Sandeep G | Sangle ^MU^ |
| Rama Krishna | Sanjeev ^MV^ |
| Sathish | Sankar ^MW^ |
| Senthilkumar | Sankararaman ^MX, MY^ |
| Prasanna K | Santhekadur ^MZ^ |
| Sivan Yegnanarayana Iyer | Saraswathy ^NA^ |
| Siddaram Shivaji | Sarate ^NB^ |
| Aswini | Saravanan ^NC, ND^ |
| Sachin C | Sarode ^NE^ |
| Mukesh Kumar | Sathya Narayanan ^NF^ |
| Maheswar | Satpathy ^NG, NH^ |
| Shakta Mani | Satyam ^NI^ |
| Jennifer | Saulam ^NJ^ |
| Ganesh Kumar | Saya ^NK^ |
| Durairaj | Sekar ^II^ |
| Siddharthan | Selvaraj ^NL^ |
| Vimalraj | Selvaraj ^NM^ |
| Pallav | Sengupta ^NN^ |
| Subramanian | Senthilkumaran ^NO^ |
| Muhammed | Shabil ^NP^ |
| Disha Sanjivkumar | Shah ^GF^ |
| Shipraa Arpan | Shah ^NQ^ |
| Sweni | Shah ^NR^ |
| Uzma | Shahab ^NS^ |
| Syed Monowar Alam | Shahid ^NT^ |
| Muhammad Aaqib | Shamim ^NC^ |
| Mohd | Shanawaz ^LC^ |
| Devika | Shanmugasundaram ^NU^ |
| Mohammed | Shannawaz ^LY^ |
| Amit Baran | Sharangi ^NV, NW^ |
| Ankita | Sharma ^NX^ |
| Arkansh | Sharma ^NY^ |
| Avimanu | Sharma ^NZ^ |
| Bhoopesh Kumar | Sharma ^OA^ |
| Bunty | Sharma ^OB^ |
| Dolly | Sharma ^OC^ |
| Kamlesh | Sharma ^CT, OD^ |
| Manoj | Sharma ^OE^ |
| Ravi Kumar | Sharma ^OF^ |
| Ujjawal | Sharma ^OG^ |
| Vishal | Sharma ^OH^ |
| Rekha Raghuveer | Shenoy ^DK^ |
| Subuhi | Sherwani ^FZ, J^ |
| Adithi | Shetty ^OI^ |
| B Suresh Kumar | Shetty ^JT^ |
| Mahabalesh | Shetty ^OJ^ |
| Premalatha K | Shetty ^OK^ |
| Shiran | Shetty ^OL^ |
| Suraj S | Shetty ^OM^ |
| Arun | Shirali ^ON^ |
| Priyanka Arun | Shirali ^OO^ |
| Dharmsheel | Shrivastav ^OP^ |
| Samra | Siddiqui ^OQ^ |
| Akanksha | Singh ^LY^ |
| Amit | Singh ^HI, OR^ |
| Baljinder | Singh ^OS^ |
| Brij Pal | Singh ^OT^ |
| Harmanjit | Singh ^OU^ |
| Harpreet | Singh ^OV^ |
| Jasvinder A | Singh ^OW, OX^ |
| Jawahar | Singh ^OY^ |
| Kalpana | Singh ^OZ^ |
| Mitasha | Singh ^PA^ |
| Paramdeep | Singh ^PB^ |
| Poornima Suryanath | Singh ^LY^ |
| Puneetpal | Singh ^PC^ |
| Samer | Singh ^PD^ |
| Satwinder | Singh ^PE^ |
| Shilpy | Singh ^PF^ |
| Siddharth | Singh ^PG^ |
| Surendra | Singh ^PH^ |
| Vanshika | Singh ^PI^ |
| Yadvinder | Singh ^PJ^ |
| Abhijeet Prasad | Sinha ^PK^ |
| Mukesh Kumar | Sinha ^HE^ |
| Indra | Sivakumar ^MW^ |
| Farrukh | Sobia ^LO^ |
| Suha | Soni ^PL, PM^ |
| Prashant | Sood ^JB^ |
| Chandrashekhar T | Sreeramareddy ^PN^ |
| Kannan | Sridharan ^PO^ |
| Vignes Anand | Srinivasalu ^PP^ |
| Shyamkumar | Sriram ^PQ^ |
| Vipul | Srivastava ^PR^ |
| Vetriselvan | Subramaniyan ^PS^ |
| Meenakshi Sundari | Subramaniyan Natarajan ^PT^ |
| Alisha | Suhag ^PU^ |
| Thanigaivel | Sundaram ^AD^ |
| Chandan Kumar | Swain ^HR^ |
| Shreya P | Swami ^HP^ |
| Azharuddin Sajid | Syed Khaja ^PV^ |
| Jabeen | Taiba ^PW^ |
| Jay | Tewari ^PX^ |
| Lakshmi | Thangavelu ^PY^ |
| Kavumpurathu Raman | Thankappan ^PZ^ |
| Rekha | Thapar ^FC^ |
| Suresh | Thareja ^QA^ |
| Mahalakshmi | Thayumana Sundaram ^QB^ |
| Jeevarathinam | Thirumalai ^MW^ |
| Muthu | Thiruvengadam ^QC^ |
| Rekha | Thiruvengadam ^CT, QD^ |
| Nihal | Thomas ^EX^ |
| Nikhil Kenny | Thomas ^QE^ |
| Vikas Kumar | Tiwari ^QF, QG^ |
| Marcos Roberto | Tovani-Palone ^II^ |
| Santhosh Kumar | Tumkur Narayanappa ^QH, QI^ |
| Shadab | Uddin ^LC^ |
| Srikanth | Umakanthan ^QJ, QK^ |
| Bhaskaran | Unnikrishnan ^QL^ |
| Era | Upadhyay ^NZ^ |
| Dipan | Uppal ^QM^ |
| Rakesh | V R ^CT, QN^ |
| Asokan Govindaraj | Vaithinathan ^QO^ |
| Sai Mahesh | Vajjala ^QP, QQ^ |
| Joe | Varghese ^LO^ |
| Pavani | Varma ^QR^ |
| Sampara | Vasishta ^QS^ |
| Vishnu Priya | Veeraraghavan ^QT^ |
| Anoop | Velayudhan ^QU, QV^ |
| U | Venkatesh ^QW^ |
| Baskar | Venkidasamy ^QX^ |
| Akshaya Kumar | Verma ^QY^ |
| Poonam | Verma ^IW^ |
| Ramesh | Vidavalur ^QZ^ |
| Sharath Chaitanya | Vipparthy ^RA^ |
| Athul | Vivek ^RB^ |
| Hai Nam | Vu ^B^ |
| Megha | Walia ^OA^ |
| Tanveer A A | Wani ^RC^ |
| Dharmveer | Yadav ^RD^ |
| Suresh | Yadav ^BC^ |
| Vikas | Yadav ^RE^ |
| Saba | Yahoo ^QR^ |
| S | Yogeshkumar ^RF^ |
| Ghazala | Yunus ^RG^ |
| Seema | Zargar ^RH^ |
| Sanjay | Zodpey ^A^ |
| Christopher J L | Murray ^B, C^ |
| Lalit | Dandona ^A, B^ |
| Austin E | Schumacher ^B^ |
| Nicholas J | Kassebaum ^B, C, RI^ |
|  |  |

# Affiliations

^A^ Public Health Foundation of India, New Delhi, India

^B^ Institute for Health Metrics and Evaluation, University of Washington, Seattle, WA, USA

^C^ Department of Health Metrics Sciences, School of Medicine, University of Washington, Seattle, WA, USA

^D^ Amity Institute of Public Health and Hospital Administration, Amity University Noida, Noida, India

^E^ National Institute of Epidemiology, Indian Council of Medical Research, Chennai, India

^F^ Department of Restorative Dentistry, University of Sharjah, Sharjah, United Arab Emirates

^G^ Department of Anaesthesia and Critical Care, Bhopal Memorial Hospital and Research Centre, Bhopal, India

^H^ Department of Oral Pathology and Microbiology, JSS Academy of Higher Education and Research, Mysuru, India

^I^ Apollo Institute of Medical Sciences & Research Chittoor, Apollo Hospital, Chittoor, India

^J^ Department of Biology, University of Hail, Hail, Saudi Arabia

^K^ Vadu Rural Health Program, KEM Hospital Research Centre, Pune, India

^L^ Department of Oncology, Post Graduate Institute of Medical Education and Research, Chandigarh, India

^M^ College of Medicine, Shaqra University, Shaqra, Saudi Arabia

^N^ School of Medicine and Psychology, Australian National University, Canberra, ACT, Australia

^O^ Health Research Institute, University of Canberra, Canberra, NSW, Australia

^P^ Department of Computer Science & Information Systems, AlMaarefa University, Riyadh, Saudi Arabia

^Q^ Department of Biosciences, University of Hail, Hail City, Saudi Arabia

^R^ College of Nursing, Majmaah University, Al Majmaah, Saudi Arabia

^S^ College of Medicine, Imam Mohammad Ibn Saud Islamic University, Riyadh, Saudi Arabia

^T^ Brody School of Medicine, East Carolina University, Greenville, NC, USA

^U^ Department of Statistics and Operations Research, Aligarh Muslim University, Aligarh, India

^V^ Department of Pharmacy, Mohammed Al-Mana College for Medical Sciences, Dammam, Saudi Arabia

^W^ Department of Biosciences, Jamia Millia Islamia, New Delhi, India

^X^ SRM School of Public Health, Chennai, India

^Y^ Division of Medical Research, Sri Ramaswamy Memorial Institute of Science and Technology, Chengalpattu, India

^Z^ Ashok & Rita Patel Institute of Physiotherapy, Charotar University of Science and Technology, Petlad, India

^AA^ Department of Public Health, The Apollo University, Chittoor, India

^AB^ Department of Biochemistry, University of Hail, Hail, Saudi Arabia

^AC^ Department of Head and Neck Oncology, HCG Cancer Hospital, Bengaluru, India

^AD^ Department of Biotechnology, Sri Ramaswamy Memorial Institute of Science and Technology, Kattankulathur, India

^AE^ Department of Pharmacy Practice, AlMaarefa University, Riyadh, Saudi Arabia

^AF^ Department of Basic Sciences, Riyadh ELM University, Riyadh, Saudi Arabia

^AG^ Department of Food Technology, Jamia Hamdard, New Delhi, India

^AH^ Department of Biostatistics, CSIR-Central Drug Research Institute, Lucknow, India

^AI^ Department of BIostatistics, Academy of Scientifc and Innovative Research (AcSIR), Ghaziabad, India

^AJ^ School of Public Health, JSS Academy of Higher Education and Research, Mysuru, India

^AK^ Biostatistics, Epidemiology, and Data Management (BEAD) Core, Johns Hopkins University, Baltimore, MD, USA

^AL^ Department of Population Medicine, Qatar University, Doha, Qatar

^AM^ Saveetha Medical College and Hospital, Saveetha University, Chennai, India

^AN^ Division of Biological Sciences, Tamil Nadu State Council for Science and Technology, Chennai, India

^AO^ Department of Clinical Sciences, Shaqra University, Shaqra, Saudi Arabia

^AP^ Division of Cardiology, University of Texas, Dallas, TX, USA

^AQ^ Department of Forensic Science, Government Institute of Forensic Science, Nagpur, Nagpur, India

^AR^ Department of Human Anatomy and Histology, I.M. Sechenov First Moscow State Medical University, Moscow, Russia

^AS^ Department of Biochemistry, National University of Science and Technology, Sohar, Oman

^AT^ Department of Biostatistics, ICMR-National Institute for Research in Reproductive and Child Health, Mumbai, India

^AU^ Community Medicine Department, Central University of Tamil Nadu, Madurai, India

^AV^ Department of Oral Pathology and Microbiology, Krishna Vishwa Vidyapeeth (Deemed to be University), Karad, India

^AW^ Health Systems Strengthening, Global Health Advocacy Incubator, Washington, DC, USA

^AX^ Centre for Policy Research and Data Analytics in Health and Environment, All India Institute of Medical Sciences, Gorakhpur, India

^AY^ Department of Community and Family Medicine, All India Institute of Medical Sciences, Rishikesh, India

^AZ^ Community Health Department, University of South Wales, South Wales, UK

^BA^ Amity Institute of Public Health, Amity University, Noida, India

^BB^ Department of Anatomy, All India Institute of Medical Sciences, Jodhpur, India

^BC^ National Institute for Implementation Research on Non-Communicable Diseases, Indian Council of Medical Research, Jodhpur, India

^BD^ Global Health Neurology Lab, NSW Brain Clot Bank, Sydney, NSW, Australia

^BE^ Division of Cerebrovascular Medicine and Neurology, National Cerebral and Cardiovascular Center, Suita, Japan

^BF^ Department of Zoology, Aligarh Muslim University, Aligarh, India

^BG^ Family Medicine Department, Texas Tech University, El Paso, TX, USA

^BH^ Translational and Clinical Research Institute, Newcastle University, Newcastle upon Tyne, UK

^BI^ Department of Community and Family Medicine, All India Institute of Medical Sciences, Deoghar, India

^BJ^ Department of Medical Lab Technology, Chandigarh University, Mohali, India

^BK^ Laboratory of Translational Medicine and Nanotherapeutics, Central University of Punjab, Bathinda, India

^BL^ Centre for the Study of Regional Development, Jawahar Lal Nehru University, New Delhi, India

^BM^ Department of Community and Family Medicine, All India Institute of Medical Sciences, Ramanathapuram, India

^BN^ Department of Pathology, Apollo Institute of Medical Sciences and Research Chittoor, Chittoor, India

^BO^ Department of Community Medicine, Apollo Institute of Medical Sciences and Research, Chittoor, India

^BP^ JSS Dental College & Hospital, Jagadguru Sri Shivarathreeswara Academy of Health Education and Research, Mysore, India

^BQ^ Non-communicable Diseases Division, National Institute of Epidemiology, Chennai, India

^BR^ Clinical Nutrition Department, Jazan University, Jazan, Saudi Arabia

^BS^ Symbiosis School of Sports Sciences, Symbiosis International University, Pune, India

^BT^ Department of Public Health and Health Sciences, Tennessee State University (TSU), Nashville, TN, USA

^BU^ Department of Community Medicine, Datta Meghe Institute of Medical Sciences, Sawangi, India

^BV^ Department of Biology, Imam Mohammad Ibn Saud Islamic University, Riyadh, Saudi Arabia

^BW^ Department of Public Health, Indian Institute of Public Health, Hyderabad, India

^BX^ Department of Physiology, University of Tennessee, Memphis, TN, USA

^BY^ Department of Oral Medicine and Radiology, King George's Medical University, Lucknow, India

^BZ^ National Institute of Translational Virology and AIDS Research, Indian Council of Medical Research, Pune, India

^CA^ SRM School of Public Health, Sri Ramaswamy Memorial Institute of Science and Technology, Chennai, India

^CB^ Department of Biosciences, Saveetha University, Chennai, India

^CC^ Department of Pulmonary Medicine, Christian Medical College and Hospital (CMC), Vellore, India

^CD^ Department of Public Health and Primary Care, University of Cambridge, Cambridge, UK

^CE^ Cardiovascular Metabolic Translational Research Program, National University of Singapore, Singapore, Singapore

^CF^ Division of Medical Research, Sri Ramaswamy Memorial Institute of Science and Technology, Kattankulathur, India

^CG^ Department of Community Medicine, Employees' State Insurance Model Hospital, Chennai, India

^CH^ International Center for Materials Sciences and Technology, Western Caspian University, Baku, Azerbaijan

^CI^ Jadara Research Center, Jadara University, Irbid, Jordan

^CJ^ Division of Gynecology Oncology, University of Alabama at Birmingham, Birmingham, AL, USA

^CK^ Chettinad Hospital & Research Institute, Chettinad Academy of Research and Education, Chennai, India

^CL^ JSS Medical College, Jagadguru Sri Shivarathreeswara Academy of Health Education and Research, Mysuru, India

^CM^ Centre for Atmospheric Sciences, Indian Institute of Technology Delhi, New Delhi, India

^CN^ School of Health, Policy and Management, Korea University, Seoul, South Korea

^CO^ Sheffield Teaching Hospitals NHS Foundation Trust, Sheffield, UK

^CP^ Division of Pathology, ICAR-Indian Veterinary Research Institute, Bareilly, India

^CQ^ Research and Development Cell, Dr. D. Y. Patil Vidyapeeth, Pune (Deemed to be University), Pune, India

^CR^ Health Information Management, Manipal Academy of Higher Education, Manipal, India

^CS^ Manipal College of Health Professions, Manipal Academy of Higher Education, Udupi, India

^CT^ India

^CU^ Independent Consultant, Bridgewater, NJ, USA

^CV^ Department of Cardiology, Hackettstown Medical Center, Hackettstown, NJ, USA

^CW^ Newton Medical Center, Sparta, NJ, USA

^CX^ NIMS Institute of Public Health and Governance, NIMS University Rajasthan, Jaipur, India

^CY^ Department of Pharmacology, All India Institute of Medical Sciences, Rajkot, India

^CZ^ College of Medicine, Ajman University, Ajman, United Arab Emirates

^DA^ Department of Dermatology, Venereology and Leprology, Apollo Institute of Medical Sciences and Research Chittoor, Chittoor, India

^DB^ Department of Pharmaceutical Sciences, Dibrugarh University, Dibrugarh, India

^DC^ Deparatment of Pharmacology, Saveetha Dental College and Hospitals, Saveetha University, Chennai, India

^DD^ College of Medicine, AlMaarefa University, Riyadh, Saudi Arabia

^DE^ Department of Clinical Laboratory Sciences, Jouf University, Sakaka, Saudi Arabia

^DF^ Medical Center, Cedar Sinnai Medical Center, Los Angeles, CA, USA

^DG^ School of American Education, Institute of Health & Management, Australia, Melbourne, VIC, Australia

^DH^ School of Engineering, Swinburne University of Technology, Melbourne, VIC, Australia

^DI^ Laboratory Sciences Division, National Institute of Epidemiology, Chennai, India

^DJ^ Department of Emergency Medicine, All India Institute of Medical Sciences (AIIMS), Madurai, Madurai, India

^DK^ Department of Pharmacology, Manipal Academy of Higher Education, Manipal, India

^DL^ Central University of Punjab, Central University of Punjab, Bathinda, India

^DM^ Amity Institute of Pharmacy, Amity University Uttar Pradesh, Noida, India

^DN^ Department of Medicine, Rutgers University, New Brunswick, NJ, USA

^DO^ Department of Zoology, KKS Women's College, Balasore, India

^DP^ Department of Medicine, All India Institute of Medical Sciences, Bathinda, India

^DQ^ Department of Pharmaceutical Sciences and Drug Research, Punjabi University, Patiala, Patiala, India

^DR^ Department of Health Systems and Policy Research, Indian Institute of Public Health, Gandhinagar, India

^DS^ College of Health Sciences, University of Sharjah, Sharjah, United Arab Emirates

^DT^ Department of Cardiology, Icahn School of Medicine at Mount Sinai, New York, NY, USA

^DU^ Department of Pediatrics, All India Institute of Medical Sciences, New Delhi, India

^DV^ Department of Nephrology, Max Super Speciality Hospital, New Delhi, India

^DW^ Centre for Toxicovigilance & Drug Safety, Manipal Academy of Higher Education, Manipal, India

^DX^ Department of Anaesthesia, Maulana Azad Medical College, New Delhi, India

^DY^ Department of Microbiology and Parasitology, Umm Al-Qura University, Makkah, Saudi Arabia

^DZ^ Epidemiology Programme, London School of Hygiene & Tropical Medicine, London, UK

^EA^ Department of Community Medicine, Post Graduate Institute of Medical Education and Research, Chandigarh, India

^EB^ Centre for Community Medicine, All India Institute of Medical Sciences, New Delhi, India

^EC^ Department of Computer and Information Science, Majmaah University, Al Majmaah, Saudi Arabia

^ED^ Faculty Of Dentistry, Jamia Millia Islamia, New Delhi, India

^EE^ Department of Pharmaceutical Technology, Adamas University, Kolkata, India

^EF^ Centre for Interdisciplinary Research in Basic Sciences, Jamia Millia Islamia, New Delhi, India

^EG^ School of Engineering and Technology, Duy Tan University, Da Nang, Viet Nam

^EH^ Kasturba Medical College, Mangalore, Manipal Academy of Higher Education, Manipal, India

^EI^ Department of AI, Galgotias University, Greater Noida, India

^EJ^ Pharmacology and Toxicology, University of Hail, Hail, Saudi Arabia

^EK^ Department of Clinical Pharmacy, Prince Sattam bin Abdulaziz University, Al Kharj, Saudi Arabia

^EL^ Center for Health Research, Northern Border University, Arar, Saudi Arabia

^EM^ Department of Health Research, ICMR National Institute for Research in Tuberculosis, Chennai, India

^EN^ Department of Health information Management, Buraydah Private Colleges, Al-Qassim, Saudi Arabia

^EO^ Saudi Arabia

^EP^ International Institute of Migration and Development, Trivandrum, Trivandrum, India

^EQ^ Department of Microbiology, University of Maiduguri, Maiduguri, Nigeria

^ER^ Department of Biotechnology, Sharda University, Greater Noida, India

^ES^ College of Nursing, All India Institute of Medical Sciences, Bhubaneswar, India

^ET^ Department of Health Informatics, Qassim University, Buraydah, Saudi Arabia

^EU^ SRM Medical College Hospital and Research Centre, Sri Ramaswamy Memorial Institute of Science and Technology, Kattankulathur, India

^EV^ Department of Community and Family Medicine, All India Institute of Medical Sciences, Rajkot, India

^EW^ Department of Biochemistry, Government Medical College, Mysuru, India

^EX^ Department of Endocrinology, Diabetes and Metabolism, Christian Medical College and Hospital (CMC), Vellore, India

^EY^ Department of Medicine, University of Melbourne, Melbourne, VIC, Australia

^EZ^ School of Medicine, Johns Hopkins University, Baltimore, MD, USA

^FA^ NGSM Institute of Pharmaceutical Sciences, Nitte University, Mangalore, India

^FB^ Department of Community Medicine, Jubilee Mission Medical College & Research Institute, Thrissur, Thrissur, India

^FC^ Department of Community Medicine, Manipal Academy of Higher Education, Mangalore, India

^FD^ Institute of National Importance, All India Institute of Medical Sciences, Rajkot, India

^FE^ Department of Pathology, Saveetha University, Chennai, India

^FF^ Department of Oral and Maxillofacial Pathology and Microbiology, Krishna Vishwa Vidyapeeth Deemed to be University, Karad, India

^FG^ Department of Endocrinology, Bharti Hospital Karnal, Karnal, India

^FH^ University Centre for Research and Development, Chandigarh University, Mohali, India

^FI^ Department of Pharmaceutical Chemistry, Prince Sattam bin Abdulaziz University, Al Kharj, Saudi Arabia

^FJ^ Prasanna School of Public Health, Manipal Academy of Higher Education, Manipal, India

^FK^ Care and Public Health Research Institute (CAPHRI), Maastricht University, Maastricht, Netherlands

^FL^ Department of Community Medicine, ESIC Medical College and Hospital Chennai, Chennai, India

^FM^ Analytical and Applied Economics, Utkal University, Bhubaneswar, India

^FN^ School of Management, The Apollo University, Chittoor, India

^FO^ Department of Physical Therapy and Health Rehabilitation, Majmaah University, Al Majmaah, Saudi Arabia

^FP^ Amity Stem Cell Institute (ASCI), Amity University Haryana, Gurugram, India

^FQ^ Department of Gastroenterology, State University of New York, Syracuse, NY, USA

^FR^ Department of Radiology, Columbia University Medical Center, New York, NY, USA

^FS^ Indian Institute of Public Health-Delhi, Public Health Foundation of India, New Delhi, India

^FT^ Department of Surgery and Cancer, Imperial College London, London, UK

^FU^ Department of Clinical Research and Epidemiology, Institute of Liver and Biliary Sciences, New Delhi, India

^FV^ Epidemiology Program, Jazan University, Jazan, Saudi Arabia

^FW^ Department of Basic Health Sciences, Qassim University, Buraydah, Saudi Arabia

^FX^ Department of Biotechnology and Microbiology, Khandelwal College of Management, Science and Technology, Bareilly, India

^FY^ Department of Chemistry, University of Hail, Hail, Saudi Arabia

^FZ^ Medical and Diagnostic Research Center, University of Hail, Hail, Saudi Arabia

^GA^ Department of Basic Medical Sciences, AlMaarefa University, Riyadh, Saudi Arabia

^GB^ College of Medicine, University of Hail, Hail, Saudi Arabia

^GC^ Institute of Research and Development, Duy Tan University, Da Nang, Viet Nam

^GD^ School of Engineering & Technology, Duy Tan University, Da Nang, Viet Nam

^GE^ Department of Public Health, New Mexico State University, Las Cruces, NM, USA

^GF^ Ashok & Rita Patel Institute of Physiotherapy, Charotar University of Science and Technology, Changa, India

^GG^ Department of Community Medicine, Manipal Academy of Higher Education, Manipal, India

^GH^ School of Pharmaceutical Sciences, CGC University, Mohali, Mohali, India

^GI^ Department of Public Health Dentistry, Krishna Vishwa Vidyapeeth (Deemed to be University), Karad, India

^GJ^ Kasturba Medical College, Manipal, Manipal Academy of Higher Education, Udupi, India

^GK^ Department of Anthropology, Panjab University, Chandigarh, India

^GL^ Department of Physiology, Sree Balaji Medical College and Hospital, Chennai, India

^GM^ Department of Oral Biology, Saveetha University, Chennai, India

^GN^ Centre for Biotechnology, Siksha 'O' Anusandhan Deemed to be University, Bhubaneswar, India

^GO^ Rajendra Institute of Medical Sciences, Ministry of Health and Family Welfare, Ranchi, India

^GP^ Kasturba Medical College, Manipal, Manipal Academy of Higher Education, Manipal, India

^GQ^ Amity Institute of Health Allied Sciences, Amity University Noida, Uttar Pradesh, Noida, India

^GR^ Department of Community Medicine, Rajendra Institute of Medical Sciences, Ranchi, India

^GS^ Department of Pediatrics, Post Graduate Institute of Medical Education and Research, Chandigarh, India

^GT^ Department of Mathematics, Amity University Haryana, Gurugram, India

^GU^ Lady Hardinge Medical College, Lady Hardinge Medical College, New Delhi, India

^GV^ Department of Physical Medicine and Rehabilitation, University of Utah Health, Salt Lake City, UT, USA

^GW^ Wits Advanced Drug Delivery Platform Research Unit, University of the Witwatersrand, Johannesburg, South Africa

^GX^ Wits Infectious Diseases and Oncology Research Institute, University of the Witwatersrand, Johannesburg, South Africa

^GY^ Department of Paediatrics, Sri Ramaswamy Memorial Institute of Science and Technology, Chennai, India

^GZ^ Department of Health Management, University of Hail, Hail, Saudi Arabia

^HA^ Faculty of Education, Shree Guru Gobind Singh Tricentenary University, Gurugram, India

^HB^ Department of Economics, Central University of Haryana, Jant-Pali, India

^HC^ Department of Anaesthesiology, Rajendra Institute of Medical Sciences, Ranchi, India

^HD^ IITM Pravartak Technologies Foundation, IITM Pravartak Technologies Foundation, Chennai, India

^HE^ Department of Physiotherapy, Manipal Academy of Higher Education, Manipal, India

^HF^ Department of Obstetrics and Gynecology, Sri Ramaswamy Memorial Institute of Science and Technology, Chengalpattu, India

^HG^ Nursing & Midwifery Research Department (NMRD), Hamad Medical Corporation, Doha, Qatar

^HH^ Division of Cardiology, University of Texas, Galveston, TX, USA

^HI^ Department of Microbiology, Central University of Punjab, Bathinda, India

^HJ^ Division of Evidence Synthesis, Foundation for People-centric Health Systems, New Delhi, India

^HK^ Division of Lifestyle Medicine, Centre for Health: The Specialty Practice, New Delhi, India

^HL^ Department of Chemistry, Dayalbagh Educational Institute, Agra, India

^HM^ Indian Council of Medical Research, New Delhi, India

^HN^ Department of Otorhinolaryngology, Father Muller Medical College, Mangalore, India

^HO^ Department of Pharmaceutical Regulatory Affairs and Management, Manipal Academy of Higher Education, Manipal, India

^HP^ Ashok & Rita Patel Institute of Physiotherapy, Charotar University of Science and Technology, Anand, India

^HQ^ Department of Community and Family Medicine, All India Institute of Medical Sciences, Bathinda, India

^HR^ Department of Analytical and Applied Economics, Utkal University, Bhubaneswar, India

^HS^ Department of Biochemistry, All India Institute of Medical Sciences, Gorakhpur, India

^HT^ Institute of Health, Jimma University, Jimma, Ethiopia

^HU^ Division of Research and Development, Lovely Professional University, Phagwara, India

^HV^ Internal Medicine Department, MedStar Health, Washington, DC, USA

^HW^ Corporate Nursing and Midwifery Research Department, Hamad Medical Corporation, Doha, Qatar

^HX^ Department of Medicine, Tirunelveli Medical College, Tirunelveli, India

^HY^ Research Institute for Medical and Health Science, University of Sharjah, Sharjah, United Arab Emirates

^HZ^ College of Medicine and Health Sciences, National University of Science and Technology, Oman, Sohar, Oman

^IA^ Department of Community Medicine, Sri Balaji Vidyapeeth (Deemed to be University), Pondicherry, India

^IB^ Centre for Clinical Pharmacology, Sri Ramaswamy Memorial Institute of Science and Technology, Chennai, India

^IC^ Department of Community Medicine, Geetanjali Medical College and Hospital in Udaipur India, Udaipur, India

^ID^ Turning Point, Eastern Health Clinical School, Monash University, Melbourne, VIC, Australia

^IE^ Department of Medical Microbiology and Immunology, Trinity Medical Sciences University, St. Vincent, Saint Vincent and the Grenadines

^IF^ Kasturba Medical College, Manipal Academy of Higher Education, Mangalore, India

^IG^ Department of Pathology, Imam Abdulrahman Bin Faisal University, Dammam, Saudi Arabia

^IH^ Multidisciplinary Department of Medical-Surgical and Dental Specialties, University of Campania Luigi Vanvitelli, Naples, Italy

^II^ Saveetha Dental College and Hospitals, Saveetha University, Chennai, India

^IJ^ Department of Public Health Dentistry, Saveetha Institute of Medical and Technical Sciences (SIMATS), Chennai, India

^IK^ Global Institute of Public Health, Ananthapuri Hospitals and Research Institute, Trivandrum, India

^IL^ Department of Community Medicine, ESIC Medical College and Hospital, Andheri, Mumbai, Mumbai, India

^IM^ Thumbay College of Management and AI in Healthcare, Gulf Medical University, Ajman, United Arab Emirates

^IN^ Research and Development Department, Panacea Institute of Interdisciplinary Research and Education, Varanasi, India

^IO^ Faculty of Medicine and Health, University of Sydney, Sydney, NSW, Australia

^IP^ School of Clinical Medicine, University of New South Wales, Sydney, NSW, Australia

^IQ^ Department of Forensic Medicine and Toxicology, All India Institute of Medical Sciences, Patna, India

^IR^ Department of Biophysics, All India Institute of Medical Sciences, New Delhi, India

^IS^ Centre For Interdisciplinary Research In Basic Sciences (CIRBSc), Jamia Millia Islamia, New Delhi, India

^IT^ Department of Medicine, Government Medical College Kozhikode, Kozhikode, India

^IU^ Department of Biochemistry, Saveetha University, Chennai, India

^IV^ School of Health Sciences, University of Petroleum and Energy Studies, Dehradun, India

^IW^ Centre for Biotechnology, Siksha 'O' Anusandhan (Deemed to be University), Bhubaneswar, India

^IX^ College of Pharmacy Department of Pharmaceutics, University of Hail, Hail, Saudi Arabia

^IY^ Department of Physiology, All India Institute of Medical Sciences, Deoghar, India

^IZ^ College of Nursing, All India Institute of Medical Sciences, Deoghar, India

^JA^ Knowledge Management Department, Prahlad Omkarwati Foundation (POF), Mumbai, India

^JB^ Independent Consultant, New Delhi, India

^JC^ Department of Anatomy, Manipal Academy of Higher Education, Mangalore, India

^JD^ Centre for Early Childhood Caries Research, Sri Ramachandra University, Chennai, India

^JE^ Santosh University, New Delhi, India

^JF^ Department of Research Methods, Orthopaedic Research Group, Coimbatore, India

^JG^ Central Research Laboratory, Vinayaka Mission’s Research Foundation (Deemed to be University), Puducherry, India

^JH^ School of Public Health Sciences and Technology, Malla Reddy Vishwavidyapeeth, Hyderabad, India

^JI^ ICMR-National Institute for Research in Tuberculosis, Indian Council of Medical Research, Chennai, India

^JJ^ Academy of Scientific and Innovative Research (AcSIR), Ghaziabad, India

^JK^ Vivekanandha College of Arts and Science for Women, Vivekanandha Educational Institution, Tiruchengode, India

^JL^ College of Medicine and Public Health, Flinders University, Adelaide, SA, Australia

^JM^ Department of Computer Science and IT, Torrens University, Adelaide, SA, Australia

^JN^ Department Health Services Research, University of Alabama at Birmingham, Birmingham, AL, USA

^JO^ Department of Oral and Craniomaxillofacial Surgery, University of Basel, Basel, Switzerland

^JP^ Department of Pediatrics Oral Health and Orthodontics, University of Basel, Basel, Switzerland

^JQ^ Dental Research Cell, Dr. D. Y. Patil University, Pune, India

^JR^ Suraj Eye Institute, Nagpur, India

^JS^ Department of Respiratory Medicine, Jagadguru Sri Shivarathreeswara Academy of Health Education and Research, Mysore, India

^JT^ Department of Forensic Medicine and Toxicology, Manipal Academy of Higher Education, Mangalore, India

^JU^ Ashok & Rita Patel Institute of Physiotherapy, Charotar University of Science and Technology, Nadiad, India

^JV^ Community Medicine and Family Medicine, All India Institute of Medical Sciences, Kalyani, India

^JW^ Institute of Physiotherapy, Srinivas University, Mangalore, India

^JX^ Community Medicine, All India Institute of Medical Sciences, Kalyani, India

^JY^ School of Sciences, Noida International University, Greater Noida, India

^JZ^ Department of Health Systems and Implementation Research, International Institute of Health Management Research, New Delhi, India

^KA^ Institute for Population and Social Research, Mahidol University, Nakhornpathom, Thailand

^KB^ Division of Health Policy and Management, University of Minnesota, Minneapolis, MN, USA

^KC^ Department of Psychiatry, All India Institute of Medical Sciences, Bhubaneswar, India

^KD^ Forensic Medicine and Toxicology, All India Institute of Medical Sciences, Hyderabad, India

^KE^ Department of Cardiology, University of Kansas Medical Center, Kansas City, KS, USA

^KF^ Department of Cardiology, US Department of Veterans Affairs (VA), Kansas City, MO, USA

^KG^ Department of Physical Therapy, Right Motion Physical Therapy PT PC., Woodbury, NY, USA

^KH^ Department of Computer Science and Bioscience, Marwadi University, Rajkot, India

^KI^ Department of Cardiovascular Medicine, University of Tennessee, Nashville, TN, USA

^KJ^ Department of Research and Training, Population Council Institute, New Delhi, India

^KK^ Zoology Section, Regional Institute of Education (RIE), Ajmer, India

^KL^ Ingenus Pharmaceuticals, LLC, Long Island University, Brooklyn, Fairfield, NJ, USA

^KM^ Department of Physiology, All India Institute of Medical Sciences, Nagpur, India

^KN^ Department of Haematology and Oncology, Shenzhen Children's Hospital, Shenzhen, China

^KO^ College of Dental Medicine, Roseman University of Health Sciences, South Jordan, UT, USA

^KP^ Department of Anesthesiology and Pain Management, University of Texas, Dallas, TX, USA

^KQ^ Department of Community Medicine, RAK Medical and Health Sciences University, Ras Al Khaimah, United Arab Emirates

^KR^ Department of Human Anatomy, All India Institute of Medical Sciences, Bathinda, India

^KS^ Department of Genetics, Yale University, New Haven, CT, USA

^KT^ Department of Community Medicine and Family Medicine, All India Institute of Medical Sciences, Hyderabad, India

^KU^ School of Public Health, Sri Ramaswamy Memorial Institute of Science and Technology, Kattankulathur, India

^KV^ Department of Data Management and Analysis, The INCLEN Trust International, New Delhi, India

^KW^ Department of Biochemistry, JSS Academy of Higher Education and Research, Mysuru, India

^KX^ Amity Institute of Forensic Sciences, Amity University, Noida, India

^KY^ Community Medicine, Maulana Azad Medical College, New Delhi, India

^KZ^ Texas Tech University Health Science Center, Texas Tech University, El Paso, TX, USA

^LA^ Maternal and Child Health Research Centre, University of Bedfordshire, Luton, UK

^LB^ Department of Biostatistics, Epidemiology, and Informatics, University of Pennsylvania, Philadelphia, PA, USA

^LC^ College of Nursing and Health Sciences, Jazan University, Jazan, Saudi Arabia

^LD^ Clinical Laboratory Sciences, King Khalid University, Abha, Saudi Arabia

^LE^ Department of Biomaterials, Saveetha University, Chennai, India

^LF^ Department of Medical Oncology, Cancer Institute (W.I.A), Chennai, India

^LG^ Department of Mathematics, Jazan University, Jazan, Saudi Arabia

^LH^ Department of Epidemiology, National Institute of Mental Health and Neurosciences, Bengaluru, India

^LI^ College of Medicine and Health Sciences, National University of Science and Technology, Sohar, Oman

^LJ^ Department of Pharmaceutical Analysis, National Institute of Pharmaceutical Education and Research, Hajipur, Hajipur, India

^LK^ Dr. Rajendra Prasad Government Medical College, Tanda, Kangra, India

^LL^ Division of Clinical Pharmacology, Manipal Academy of Higher Education, Manipal, India

^LM^ Trauma and Emergency Medicine Department, All India Institute of Medical Sciences (AIIMS), Madurai, Madurai, India

^LN^ Department of Cardiovascular Medicine, Cleveland Clinic, Cleveland, OH, USA

^LO^ Department of Public Health, Jazan University, Jazan, Saudi Arabia

^LP^ Centre for Chronic Disease Control, New Delhi, India

^LQ^ Department of Anatomy, Govt. Siddhartha Medical College, Vijayawada, India

^LR^ Department of Radiology, Stanford University, Stanford, CA, USA

^LS^ National Institute for Research in Tuberculosis, Indian Council of Medical Research, Chennai, India

^LT^ All India Institute of Medical Sciences, Bilaspur, India

^LU^ Department of Medical Imaging Technology, Manipal Academy of Higher Education, Manipal, India

^LV^ Department of Community Medicine, ESIC Medical College & PGIMSR & Model Hospital, Rajajinagar, Bengaluru, India

^LW^ Kasturba Medical College, Mangalore, Manipal Academy of Higher Education, Mangalore, India

^LX^ Department of Medicine, All India Institute of Medical Sciences, Bhubaneswar, India

^LY^ Amity Institute of Public Health and Hospital Administration, Amity University, Noida, India

^LZ^ Centre for Digital Health and Precision Medicine, The Apollo University, Chittoor, India

^MA^ RAK College of Nursing, RAK Medical and Health Sciences University, Ras Al Khaimah, United Arab Emirates

^MB^ Department of Basic Medical Sciences, Jazan University, Jazan, Saudi Arabia

^MC^ Department of Internal Medicine, Armed Forces Medical College, Pune, India

^MD^ Yenepoya Research Centre, Yenepoya University, Mangalore, India

^ME^ Faculty of Allied Health Sciences, Santosh (Deemed to be University), Delhi, India

^MF^ Department of Pharmaceutics, University of Hail, Hail, Saudi Arabia

^MG^ Department of Community Medicine, Government Medical College, Chandigarh, India

^MH^ RUSA Centre of Excellence in Public Policy and Governance, Utkal University, Bhubaneswar, India

^MI^ Saveetha College of Physiotherapy, Saveetha University, Chennai, India

^MJ^ Department of Oral and Maxillofacial Surgery, Jagadguru Sri Shivarathreeswara Academy of Health Education and Research, Mysore, India

^MK^ Department of Psychiatry, All India Institute of Medical Sciences, New Delhi, India

^ML^ College of Applied and Health Sciences, A' Sharqiyah University, Ibra, Oman

^MM^ National Institute for Research in Bacterial Infections (ICMR NIRBI), Indian Council of Medical Research, Kolkata, India

^MN^ Department of Medical Research, New Delhi, India

^MO^ Center for Global Health Research, Saveetha University, Chennai, India

^MP^ Biotechnology Research Center, Mashhad University of Medical Sciences, Mashhad, Iran

^MQ^ Department of Radiation Oncology, All India Institute of Medical Sciences, New Delhi, India

^MR^ Department of Family and Generations, International Institute for Population Sciences, Mumbai, India

^MS^ Canadian Red Cross, Red Cross, Ottawa, ON, Canada

^MT^ Department of Health Research, ICMR-Regional Medical Research Centre, Bhubaneswar, Bhubaneswar, India

^MU^ Department of Forensic Biology, Government Institute of Forensic Science Chhatrapati Sambhajinagar, Chhatrapati Sambhajinagar, India

^MV^ Department of Pediatrics, SRM Medical College Hospital And Research Centre, Kattankulathur, India

^MW^ Department of Microbiology, Saveetha University, Chennai, India

^MX^ Department of Pediatrics, University Hospitals Rainbow Babies & Children's Hospital, Cleveland, OH, USA

^MY^ Department of Pediatrics, Case Western Reserve University, Cleveland, OH, USA

^MZ^ JSS Medical College, Jagadguru Sri Shivarathreeswara Academy of Health Education and Research, Mysore, India

^NA^ Independent Consultant, Thiruvananthapuram, India

^NB^ CVM Nursing College, The Charutar Vidya Mandal (CVM) University, Anand, India

^NC^ Department of Pharmacology, All India Institute of Medical Sciences, Jodhpur, India

^ND^ Indira Gandhi Medical College and Research Institute, Puducherry, India

^NE^ Department of Oral Pathology and Microbiology, Dr. D. Y. Patil Vidyapeeth, Pune (Deemed to be University), Pune, India

^NF^ Department of Epidemiology, National Institute for Research in Tuberculosis, Chennai, India

^NG^ UGC Centre of Advanced Study in Psychology, Utkal University, Bhubaneswar, India

^NH^ Udyam-Global Association for Sustainable Development, Bhubaneswar, India

^NI^ Department of Pharmacology, RAK Medical and Health Sciences University, Ras Al Khaimah, United Arab Emirates

^NJ^ Department of Food Processing and Nutrition, Karnataka State Akkamahadevi Women University, Vijayapura, India

^NK^ Department of Preventive and Social Medicine, Jawaharlal Institute of Postgraduate Medical Education and Research, Puducherry, India

^NL^ Faculty of Dentistry, University of Puthisastra, Phnom Penh, Cambodia

^NM^ Department of Biotechnology, Sri Ramachandra University, Chennai, India

^NN^ Department of Biomedical Sciences, Gulf Medical University, Ajman, United Arab Emirates

^NO^ Emergency Department, Manian Medical Centre, Erode, India

^NP^ Department of Pharmacy Practice, MS Ramaiah University of Applied Sciences, Bangalore, India

^NQ^ Charotar University of Science and Technology (CHARUSAT), Ashok and Rita Patel Institute of Physiotherapy, Changa, India

^NR^ Rita A. Patel Institute of Physiotherapy, The Charutar Vidya Mandal (CVM) University, Anand, India

^NS^ Department of Public Health, University of Hail, Hail, Saudi Arabia

^NT^ Biochemistry Department, University of Hail, Hail, Saudi Arabia

^NU^ Division of Epidemiology & Biostatistics, National Institute of Epidemiology, Chennai, India

^NV^ Department of Plantation Spices Medicinal and Aromatic Crops, Bidhan Chandra Krishi Viswavidyalaya Agricultural University in India, Mohanpur, India

^NW^ School of Agriculture, Food and Ecosystem Sciences, University of Melbourne, Burnley, VIC, Australia

^NX^ Department of Physiotherapy, Amity University Uttar Pradesh, Noida, Noida, India

^NY^ Government Medical College, Chennai, India

^NZ^ Amity Institute of Biotechnology, Amity University Rajasthan, Jaipur, India

^OA^ Department of Forensic Science, Shree Guru Gobind Singh Tricentenary University, Gurugram, India

^OB^ Department of Biotechnology, Graphic Era (Deemed to be University), Dehradun, India

^OC^ Department of Biotechnology, Noida Institute of Engineering & Technology, Greater Noida, India

^OD^ Graphic Era (Deemed to be University), Department of Chemistry, Dehradun, India

^OE^ Department of Social and Behavioral Health, University of Nevada Las Vegas, Las Vegas, NV, USA

^OF^ Department of University Institute of Biotechnology, Chandigarh University, Gharuan, India

^OG^ Department of Human Genetics and Molecular Medicine, Central University of Punjab, Bathinda, India

^OH^ Department of Forensic Science, Panjab University, Chandigarh, India

^OI^ Department of Obstetrics and Gynaecology, Manipal Academy of Higher Education, Mangalore, India

^OJ^ Alva's Institute of Medical Sciences & Research Centre, Rajiv Gandhi University of Health Sciences, Moodubidire, India

^OK^ Manipal College of Dental Sciences, Mangalore, Manipal Academy of Higher Education, Manipal, India

^OL^ Department of Gastroenterology and Hepatology, Manipal Academy of Higher Education, Udupi, India

^OM^ KS Hegde Medical Academy, Nitte University, Mangalore, India

^ON^ Department of General Medicine, Manipal Academy of Higher Education, Manipal, India

^OO^ Department of Physiology, Manipal Academy of Higher Education, Manipal, India

^OP^ Department of Biotechnology and Microbiology, Noida International University, Greater Noida, India

^OQ^ University of Hail, Hail, Saudi Arabia

^OR^ Department of Laboratory Medicine, All India Institute of Medical Sciences, New Delhi, India

^OS^ Department of Biochemistry, Central University of Punjab, Bathinda, India

^OT^ University Centre for Research and Development (UCRD), Chandigarh University, Mohali, India

^OU^ Department of Pharmacology, Government Medical College and Hospital, Chandigarh, India

^OV^ School of Pharmaceutical Sciences, IFTM University, Moradabad, India

^OW^ School of Medicine, Baylor College of Medicine, Houston, TX, USA

^OX^ Department of Medicine Service, US Department of Veterans Affairs (VA), Houston, TX, USA

^OY^ Department of Psychiatry, All India Institute of Medical Sciences, Bathinda, India

^OZ^ Research Department, Hamad Medical Corporation, Doha, Qatar

^PA^ Department of Community Medicine, Department of Health, Delhi, India

^PB^ Department of Radiodiagnosis, All India Institute of Medical Sciences, Bathinda, India

^PC^ Department of Human Genetics, Punjabi University, Patiala, Patiala, India

^PD^ Institute of Medical Sciences, Banaras Hindu University, Varanasi, India

^PE^ Department of Computer Science & Technology, Central University of Punjab, Bathinda, India

^PF^ Department of Biotechnology, Microbiology and Forensic Science, Noida International University, Greater Noida, India

^PG^ Mehta Family School of Biosciences and Biomedical Engineering, Indian Institute of Technology Indore, Indore, India

^PH^ Department of Community Medicine, Veer Chandra Singh Garhwali Government Institute of Medical Science and Research, Srinagar Garhwal, India

^PI^ Internal Medicine Department, King George's Medical University, Lucknow, India

^PJ^ Department of Botany, Central University of Punjab, Bathinda, India

^PK^ Amity Institute of Public Health and Hospital Administration, Amity University Noida, Delhi, India

^PL^ Department of Preventive Cardiology, Houston Methodist Hospital, Houston, TX, USA

^PM^ Department of Management, Policy and Community Health, University of Texas, Houston, TX, USA

^PN^ Department of Public Health and Community Medicine, International Medical University, Kuala Lumpur, Malaysia

^PO^ Department of Pharmacology and Therapeutics, Arabian Gulf University, Manama, Bahrain

^PP^ Department of Clinical Research, National Institute for Research in Tuberculosis, Chennai, India

^PQ^ College of Health and Public Service, University of North Texas, Denton, TX, USA

^PR^ Research and Development Cell, Lovely Professional University, Phagwara, India

^PS^ Department of Medical Sciences, Sunway University, Subang Jaya, Malaysia

^PT^ Department of General Medicine, Sri Ramaswamy Memorial Institute of Science and Technology, Chengalpattu, India

^PU^ Integrative Epidemiology Unit, University of Bristol, Bristol, UK

^PV^ Pathology, University of Hail, Hail, Saudi Arabia

^PW^ Department of Environmental, Agricultural and Occupational Health, University of Nebraska Medical Center, Omaha, NE, USA

^PX^ Internal Medicine, Baptist Hospitals Of Southeast Texas, Beaumont, TX, USA

^PY^ Faculty of Medicine, University of Cyberjaya, Selangor, Malaysia

^PZ^ Amrita Vishwa Vidyapeetham, Amrita Institute of Medical Sciences, Kochi, India

^QA^ Department of Pharmaceutical Sciences and Natural Products, Central University of Punjab, Bathinda, Bathinda, India

^QB^ Department of Obstetrics and Gynecology, St. John's National Academy of Health Sciences, Miami, FL, USA

^QC^ Department of Applied Bioscience, Konkuk University, Seoul, South Korea

^QD^ Department of Neonatology, Saveetha University, Chennai, India

^QE^ Department of Gastroenterology, St. Luke's Hospital, Patanamthitta, India

^QF^ Department of Physiology, All India Institute of Medical Sciences, New Delhi, India

^QG^ Department of Systems Neuroscience, Tohoku University, Sendai, Japan

^QH^ ESIC Medical College PGIMSR and Model Hospital, Bengaluru, India

^QI^ ESIC Alumni Association, Bengaluru, India

^QJ^ Independent Consultant, St. Augustine, Trinidad and Tobago

^QK^ North Central Regional Health Authority, Champ Fleurs, Trinidad and Tobago

^QL^ Kasturba Medical College Mangalore, Manipal Academy of Higher Education, Manipal, India

^QM^ Section of Advanced Heart Failure and Transplant, Northwell Health / North Shore University Hospital, Manhasset, NY, USA

^QN^ College of Nursing, All India Institute of Medical Sciences, Nagpur, India

^QO^ College of Health and Sport Sciences, University of Bahrain, Zallaq, Bahrain

^QP^ Department of Community Medicine, Government Medical College Yadadri, Yadadri, India

^QQ^ Department of Epidemiology and Public Health, Sankhyatraya Science and Research Association, New Delhi, India

^QR^ Department of Community Medicine, Apollo Institute of Medical Sciences and Research, Hyderabad, India

^QS^ Department of Biochemistry, Apollo Institute of Medical Sciences and Research Chittoor, Chittoor, India

^QT^ Centre of Molecular Medicine and Diagnostics, Saveetha University, Chennai, India

^QU^ Communicable Diseases, Indian Council of Medical Research, New Delhi, India

^QV^ School of Epidemiology, Indian Institute of Health Management Research University, Jaipur, India

^QW^ Department of Community Medicine and Family Medicine, All India Institute of Medical Sciences, Gorakhpur, India

^QX^ Centre for Biosciences and Biotechnology, Saveetha University, Chennai, India

^QY^ Siksha 'O' Anusandhan (Deemed to be University), Bhubaneswar, India

^QZ^ Department of Pediatrics, Cornell University, Ithaca, NY, USA

^RA^ Cardiac Electrophysiology, St Bernard’s Medical Center, Jonesboro, AR, USA

^RB^ Forensic Medicine and Toxicology, Manipal Academy of Higher Education, Manipal, India

^RC^ Department of Pharmaceutical Chemistry, King Saud University, Riyadh, Saudi Arabia

^RD^ Department of Biochemistry, All India Institute of Medical Sciences, Jodhpur, India

^RE^ Department of Environmental Health and Epidemiology, National Institute for Research in Environmental Health, Bhopal, India

^RF^ Department of Community Medicine, KLE Academy of Higher Education and Research, Belagavi, India

^RG^ Department of Basic Science, University of Hail, Hail, Saudi Arabia

^RH^ Department of Biochemistry, King Saud University, Riyadh, Saudi Arabia

^RI^ Department of Anesthesiology & Pain Medicine, University of Washington, Seattle, WA, USA
